# Integrated care management for patients following acute stroke: a systematic review

**DOI:** 10.1093/qjmed/hcaf029

**Published:** 2025-01-24

**Authors:** Ian Eustace, Benjamin J R Buckley, Isik Kaya, Katie L Hoad, Madeleine France-Ratcliffe, Andrew M Hill, Gregory Y H Lip, Ian D Jones, Karen Higginbotham

**Affiliations:** School of Nursing and Advanced Practice, Liverpool John Moores University, Liverpool, UK; Liverpool Centre for Cardiovascular Science, University of Liverpool, Liverpool John Moores University and Liverpool Heart and Chest Hospital, Liverpool, UK; Liverpool Centre for Cardiovascular Science, University of Liverpool, Liverpool John Moores University and Liverpool Heart and Chest Hospital, Liverpool, UK; Cardiovascular Health Sciences, Research Institute for Sport and Exercise Sciences, Liverpool John Moores University, Liverpool, UK; West Hertfordshire Teaching Hospitals NHS Trust, Watford, UK; Cardiovascular Health Sciences, Research Institute for Sport and Exercise Sciences, Liverpool John Moores University, Liverpool, UK; Cardiovascular Health Sciences, Research Institute for Sport and Exercise Sciences, Liverpool John Moores University, Liverpool, UK; Mersey and West Lancashire Teaching Hospitals NHS Trust, Liverpool, UK; Liverpool Centre for Cardiovascular Science, University of Liverpool, Liverpool John Moores University and Liverpool Heart and Chest Hospital, Liverpool, UK; Danish Center for Health Services Research, Department of Clinical Medicine, Aalborg University, Aalborg, Denmark; School of Nursing and Advanced Practice, Liverpool John Moores University, Liverpool, UK; Liverpool Centre for Cardiovascular Science, University of Liverpool, Liverpool John Moores University and Liverpool Heart and Chest Hospital, Liverpool, UK; School of Nursing and Advanced Practice, Liverpool John Moores University, Liverpool, UK; Liverpool Centre for Cardiovascular Science, University of Liverpool, Liverpool John Moores University and Liverpool Heart and Chest Hospital, Liverpool, UK

## Abstract

Contemporary stroke care is moving towards more holistic and patient-centred integrated approaches, however, there is need to develop high quality evidence for interventions that benefit patients as part of this approach. This study aims to identify the types of integrated care management strategies that exist for people with stroke, to determine whether stroke management pathways impact patient outcomes and to identify elements of integrated stroke care that were effective at improving outcomes. The study is a systematic review with meta-analysis. The review was conducted using Medline, CINAHL, Web of Science and the Cochrane Database of randomized controlled trials from January 2012 to January 2024. Studies that evaluated interventions as part of integrated care against a control or standard treatment group were included. Primary outcomes included mortality, recurrent stroke and major bleeding. Secondary outcomes included quality of life, unplanned readmission, anxiety and depression, lifestyle and cardiovascular risk factors, and adherence to intervention. In total, 99 studies were included and 63 were meta-analysed. Patients receiving integrated stroke care had significant reductions in recurrent stroke (RR 0.79, 95% CI: 0.63–1.00, *P* = 0.05, *I*^2^ = 39%), significant improvements in quality of life (SMD = 0.41, 95% CI: 0.26–0.56, *P* < 0.00001, *I*^2^ = 91%) and reduced incidence of depression (RR 0.95, 95% CI: 0.92–0.99, *P* = 0.007, *I*^2^ = 22%). There were no significant differences in mortality or major bleeding. The findings of this study show that integrated care post-stroke is associated with better quality of life and reduced depression and recurrent stroke.

## Introduction

Effective stroke care needs an organizational structure that facilitates best treatments at the right time.^1^ Contemporary stroke care commonly involves a multidisciplinary team of healthcare professionals.^2^ Integrated care in stroke has been defined as a project network technique involving interdisciplinary interventions to improve communication and co-ordination between disciplines, providing a time frame for patient care, with regular monitoring of patient progression.^3^ However, there is no unifying definition or common conceptual understanding of integrated care, and the perspectives that construct the concept can be shaped by views and expectations of various health system stakeholders.^4^

The centralization of stroke services in parts of the UK and the formation of hyperacute stroke units (HASUs) offering continual access to stroke specialists, investigations and interventions^5^ can be considered to represent integrated care in acute stroke. The treatment of stroke patients in HASUs has resulted in reductions in mortality and length of hospital stay compared with traditional stroke units.^6^^,^^7^

Recently, there has been a move towards a more integrated or holistic management pathway for patients following acute stroke, which might prevent recurrent stroke and could also improve patient functional status, symptoms and comorbidities^2^^,^^8^ however many challenges remain. These include a lack of consensus on the optimal care pathway for patients,^9^^,^^10^ high heterogeneity among stroke treatment centres^11^ and the continuing need to develop high quality evidence for prevention of secondary vascular events and risk factor management after stroke.^12–14^

With no clear definition of integrated care in stroke and no consensus on optimal patient management in the acute and post-acute settings, some clarification on which integrated care strategies are most beneficial to patients is needed. In this study, we aimed to identify the types of integrated care management strategies that exist for people with stroke, to determine whether stroke management pathways impact patient outcomes and to identify elements of integrated care that improved patient outcomes.

## Methods

This systematic review was conducted in accordance with the guidelines of Preferred Reporting Items for Systematic Reviews and Meta-analyses (PRISMA).

### Inclusion and exclusion criteria

Studies of integrated care in stroke patients were included in the review based on NHS/WHO definitions (Supplementary Material S1). Stroke was defined using the American Heart Association/American Stroke Association (AHA/ASA) guidelines.^12^ Patients with any acute stroke or transient ischaemic attack (TIA) of any age, and any pathological type of stroke (ischaemic, venous, intracerebral haemorrhage) were included. All randomized controlled trials (RCTs), uncontrolled comparative trials, observational cohort studies, mixed methods and quantitative case studies were considered for inclusion whereas studies involving patients for which stroke was not the primary event or where a definitive diagnosis of stroke could not be confirmed were excluded.

### Search strategy

The search strategy used medical subject headings (MeSH) terms and synonyms for “stroke” and “integrated care”. These terms were combined with Boolean operators, truncations and wildcards. Studies with evidence of a multidisciplinary approach to care with or without a patient-centred focus were included. MEDLINE, CINAHL, Web of Science and the Cochrane Central Register of Controlled Trials were searched from 1 January 2012 to 1 January 2024 for relevant studies (Supplementary Material S1).

### Data extraction

Data extraction was conducted independently by I.E., K.L.H., M.F-R. and I.K. using a bespoke data extraction tool and included: (a) authors, publication year, country of origin, reference; (b) study design with inclusion/exclusion criteria; (c) aims and objectives; (d) demographic data (including *n*=, age, sex, ethnicity, disease characteristics, co-morbidities); (e) description of intervention and/or comparator; (f) outcomes (effectiveness and safety); (g) results; (h) conclusions and (i) risk of bias assessment.

### Risk of bias assessment

Risk of bias was independently assessed in duplicate by I.E. and I.K., K.L.H. or M.F-R. and discrepancies were discussed and resolved with a third reviewer (B.R.J.B). The Cochrane Risk of Bias v.2 (RoB2) tool^15^ and the Risk Of Bias In Non-randomized Studies—of Interventions (ROBINS-I)^16^ were used for randomized and non-randomized trials, respectively.

### Data synthesis

Results of the systematic review were grouped by outcome (primary: mortality, recurrent stroke, major bleeding; secondary: quality of life, unplanned readmission, depression/anxiety, treatment adherence). Meta-analyses were conducted for comparable studies where sufficient data were present. Primary and secondary outcome effect measures with 95% confidence intervals (CI) were pooled using RevMan software version 5.4.^17^ Random effects models were used, allowing for between-study variability by weighting studies using a combination of intra- and inter-study variance. Results were presented visually using Forest plots. Heterogeneity was assessed visually and using the *I*^2^ statistic with 25%, 50% and 75% considered moderate, substantial and considerable heterogeneity, respectively.

To measure overall treatment effects, the Der Simonian & Laird method for both binary (e.g. mortality) and continuous (e.g. quality of life) outcomes was used. If continuous data were not homogeneous, an estimate of the standardized mean difference (SMD) with 95% CI was calculated. If quantitative data were too few or too heterogeneous, then a narrative synthesis approach was undertaken.

### Analysis of subgroups or subsets

Mortality and other outcomes (where applicable) were analysed according to length of follow-up, via sub-group analyses to stratify for different follow-up time points. Quality of life and depression/anxiety data were grouped and analysed according to type of measurement tool (e.g. VAS, EQ-5D, HADS). Where feasible, SMDs were used to meta-analyse similar scales. Meta-analyses of randomized controlled trials (RCTs) and non-randomized trials (NRCTs) were conducted separately.

## Results

**Figure 1.**
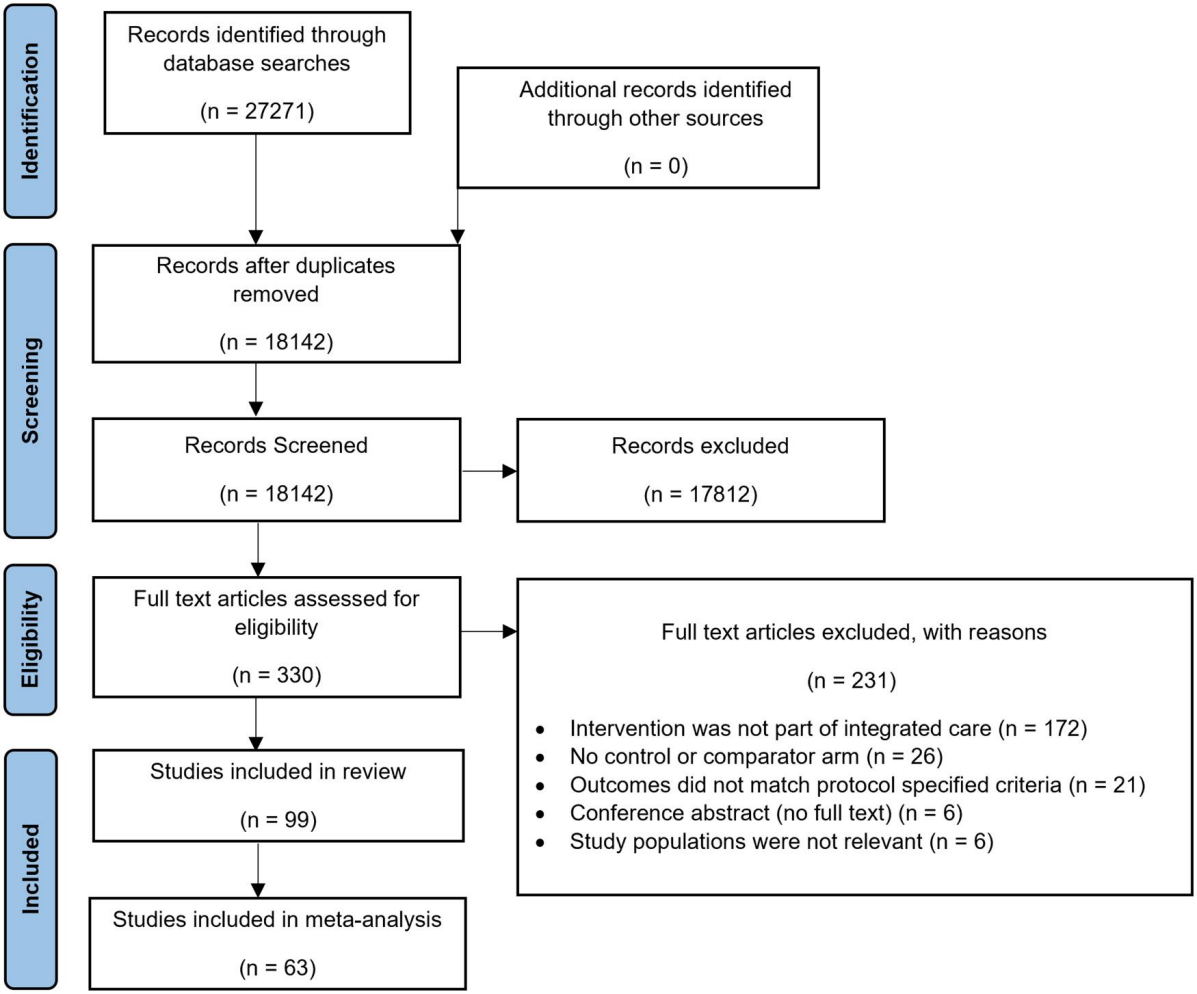
PRISMA chart for systematic review of integrated care in stroke.

An initial search yielded 27 271 records. After removal of duplicates, 330 papers were assessed for eligibility against the inclusion/exclusion criteria. Of these, 99 (30%) were included in the systematic review (Figure 1) and 63 (19%) were included in meta-analysis.

### Characteristics of the included studies

The studies included in the review were published between January 2012 and January 2024. Of the 99 studies, the majority (64) were RCTs (Supplementary Material S2). The total number of participants included in the review was 88 435, of which 40 475 received an integrated care intervention or pathway comprising multiple interventions. Mean/median patient age ranged from 53 years^18–21^ to 83 years^22^ and the proportion of females ranged from 23%^23^ to 70%^24^.

Of the 64 RCTS, 10 were considered at low risk of bias, 33 were moderate risk and 21 studies were at high risk of bias due to small numbers, study design, confounding factors or missing data. Of 35 NRCTs, 20 were considered at moderate risk of bias and 15 studies were at high risk of bias (Supplementary Material S3).

Of the 99 individual studies, 28 included an exercise intervention, 28 included an educational component, 17 included a psychological intervention and 16 assessed lifestyle and cardiovascular risk factors. Forty-two (42) studies included multiple interventions or complete pathways and 19 studies were considered patient centred. Of the intervention types, exercise, lifestyle and mental health interventions positively impacted on the majority of protocol-specified primary and secondary outcomes across studies however only exercise positively impacted all outcomes.^18^^,^^25–36^

Overall, the results of the studies were inconclusive, with 34% showing no significant benefit of the intervention over the control or comparator group and there was substantial heterogeneity between studies.

## Meta-analysis

The findings of the meta-analysis are summarized in Table 1.

**Table 1:** Summary of findings table for systematic review of integrated care

Outcomes	Relative effect (95% CI)	Number of participants (studies)	Comments
Primary			
Mortality	RCTs: RR 0.79 (0.59–1.06) NRCTs: RR 0.82 (0.45–1.49)	21 533 (13) 31 223 (9)	There were fewer death events in the integrated care groups compared with control but no significant overall difference in mortality in RCTs or NRCTs. The only significant difference in death events was observed in a subgroup analysis of long-term mortality in NRCTs (>1 year, 9404 patients, three studies, RR = 0.58, *P* = 0.0007)
Recurrent stroke	RCTs: RR 0.79 (0.63–1.00) NRCTs: RR 1.08 (0.64–1.82)	10 915 (9) 1604 (3)	There were significantly fewer recurrent strokes associated with RCTs of integrated care compared with control (*P* = 0.05) however there were no differences in NRCTs
Major bleeding	RR 1.35 (0.56–3.22)	8210 (4)	There were more major bleeding events in the integrated care groups compared with control but the difference was not statistically significant (*P* = 0.50)
Secondary			
Quality of life	SMD: 0.41 (0.26–0.56)	10 653 (21)	Integrated care interventions significantly improved quality of life (*P* < 0.00001), with the greatest difference in QoL scoring observed with the SF-36 general health questionnaire and the SSQOL scoring tools
Unplanned readmission	RCTs: RR 0.88 (0.73–1.05) NRCTs: RR 0.76 (0.37–1.57)	7236 (9) 6370 (5)	There were fewer unplanned readmission events associated with integrated care interventions however the differences were not significant in either RCTs (*P* = 0.15) or NRCTs (*P* = 0.46)
Anxiety and depression	Depression: RR 0.95 (0.92–0.99) Anxiety (HADS-A): RR 0.75 (0.57–0.99)	36 044 (7) 606 (3)	The proportion of patients with depression was significantly lower with integrated care compared with control (*P* = 0.007). An analysis of anxiety using the HADS-A assessment tool showed that the proportion of patients with anxiety was significantly lower with integrated care (*P* = 0.04)
Lifestyle and cardiovascular risk factors	SMD −0.17 (−0.25 to −0.09)	4538 (9)	Overall, integrated care was associated in a significant improvement in cardiovascular risk factors (*P* < 0.0001), with significant reductions in SBP and LDL-cholesterol (both *P* ≤ 0.03) however there were no differences between groups for total cholesterol, BMI or the proportion of patients stopping smoking
Adherence to intervention	RR 1.31 (0.77–2.23)	3646 (4)	Integrated care was associated with higher rates of adherence compared with control however the difference was not statistically significant (*P* = 0.32)
Exploratory			
Favourable outcome (mRS ≤ 2)	RR 1.02 (0.98–1.07)	4751 (4)	There was no difference in the proportion of patients with favourable outcome between integrated care and control (*P* = 0.35)
Recurrent risk of cardiovascular disease: Myocardial infarction Vascular death	RR 0.60 (0.34–1.05) RR 0.58 (0.21–1.60)	7733 (3) 4221 (2)	There were fewer events of myocardial infarction and vascular death associated with integrated care however these differences were not statistically significant (MI, *P* = 0.07; vascular death, *P* = 0.30)

Patient or population: people with stroke.

Setting: during and after care.

Intervention: Integrated care.

Comparison: Control; end of intervention.

BMI, body mass index; CI, confidence interval; HADS-A, hospital anxiety and depression scale-anxiety component; LDL, low density lipoprotein; MI, myocardial infarction; mRS, modified Rankin Scale; NRCT, nonrandomized controlled trial; RCT, randomized controlled trial, RR, risk ratio; SBP, systolic blood pressure; SMD, standardized mean difference.

### Primary outcomes

#### Mortality

**Figure 2.**
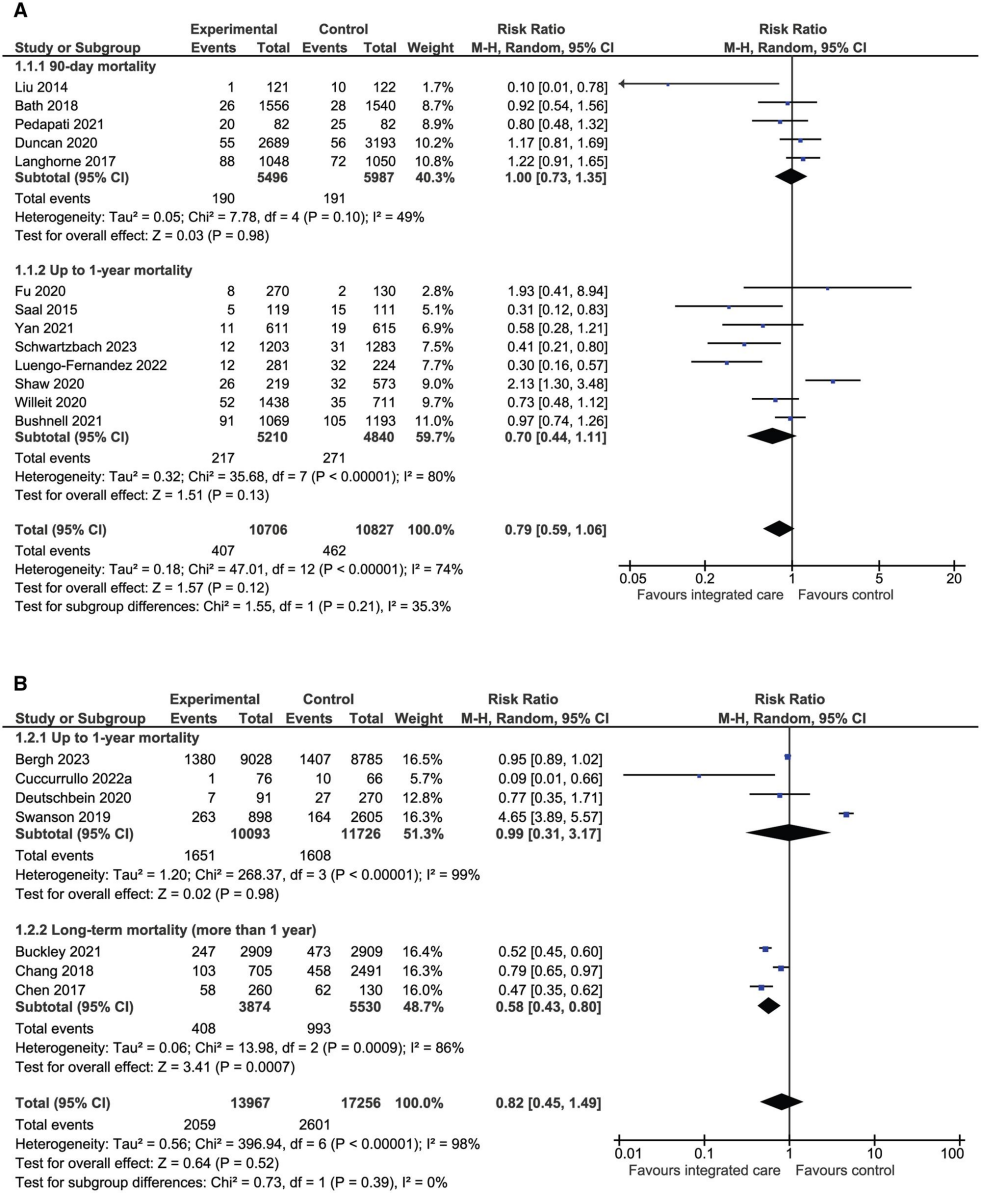
Comparison of integrated care with control for mortality (A, randomized controlled trials; B, nonrandomized trials).

A total of 13 (*n* = 21 533) RCTs compared integrated care with control for mortality (Figure 2A). Integrated care was not associated with reduced mortality (relative risk [RR] 0.79, 95% CI: 0.59–1.06, *I*^2^ = 74%). Sub-group analysis of NRCTs indicated a significant reduction in long-term (>1 year) mortality in favour of integrated care (RR 0.58, 95% CI: 0.43–0.80, *P* = 0.0007, *I*^2^ = 86%) however there were only three studies (*n* = 9404) included in this analysis (Figure 2B).

#### Recurrent stroke

**Figure 3.**
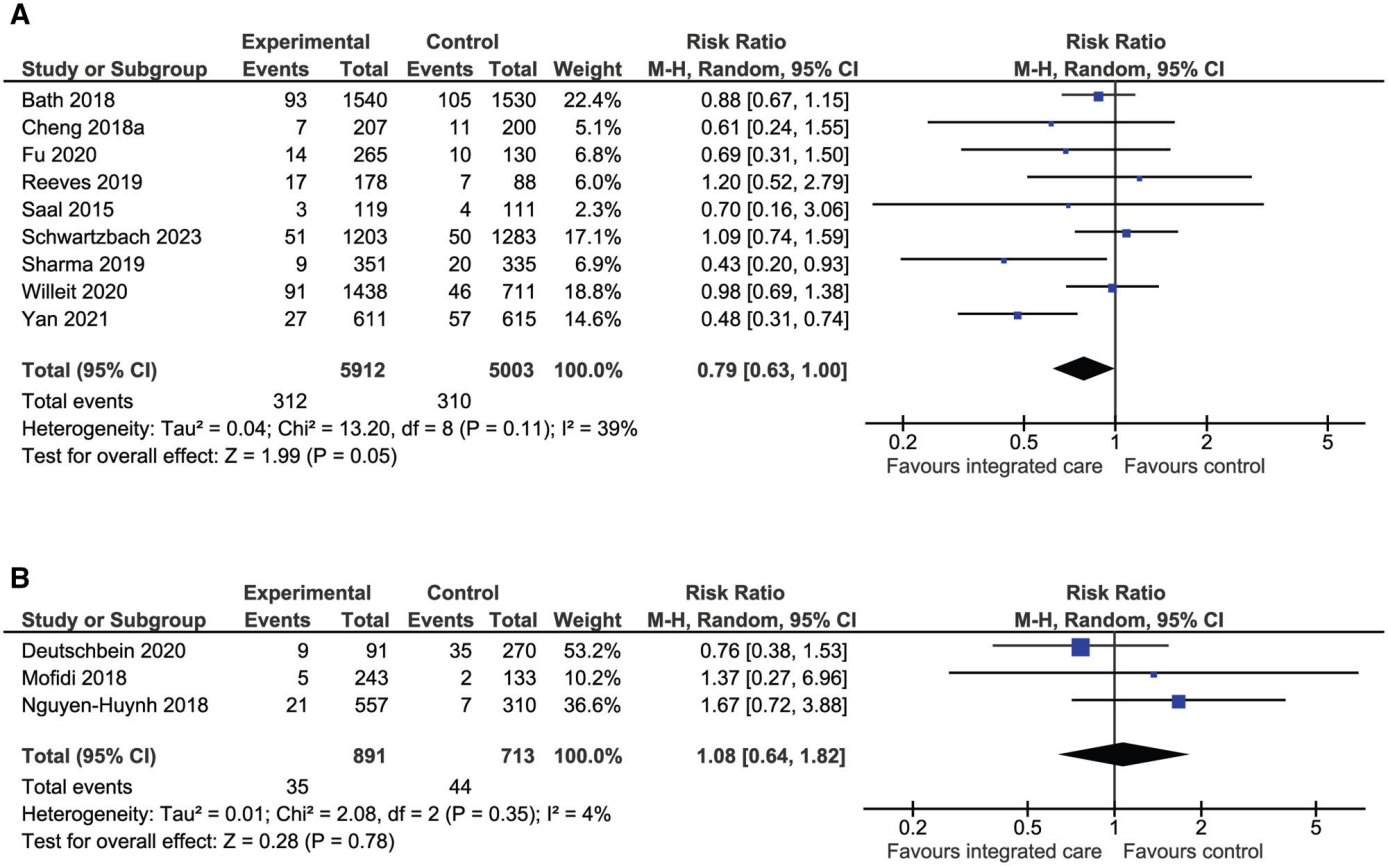
Comparison of integrated care with control for recurrent stroke (A, randomized controlled trials; B, nonrandomized controlled trials).

A total of nine (*n* = 10 915) RCTs showed a statistically significant reduction in recurrent stroke in favour of integrated care (RR 0.79, 95% CI: 0.63–1.00, *P* = 0.05, *I*^2^ = 39%, Figure 3A) however there was no difference in recurrent stroke between groups in NRCTs (RR 1.08, 95% CI: 0.64–1.82, *P* = 0.78, *I*^2^ = 4%, Figure 3B).

#### Major bleeding

**Figure 4.**
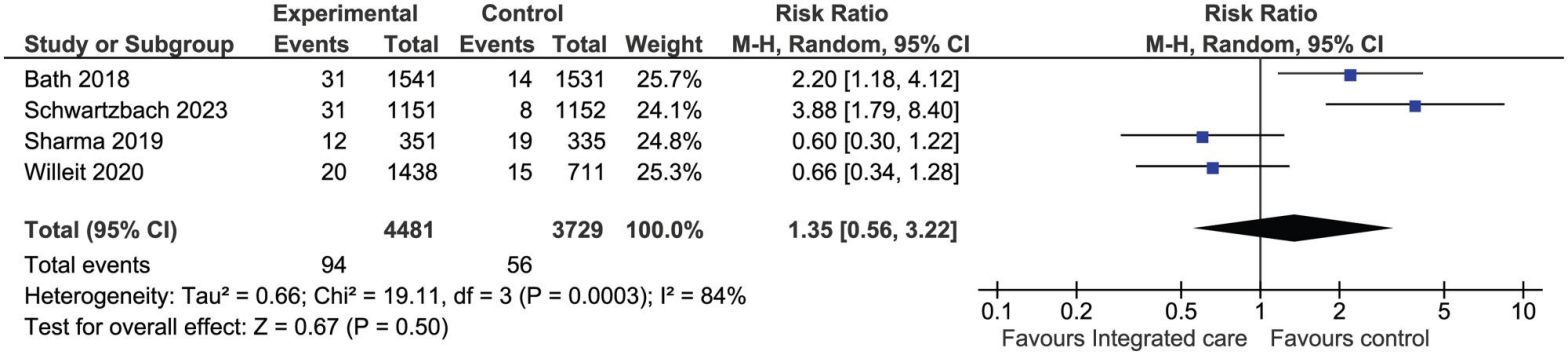
Comparison of integrated care with control for major bleeding (randomized controlled trials).

A total of four RCTs (*n* = 8210) compared integrated care with control for major bleeding (Figure 4). There were more bleeding events associated with integrated care versus control (RR 1.35, 95% CI: 0.56–3.22, *I*^2^ = 84%), however the difference was not statistically significant (*P* = 0.50).

### Secondary outcomes

#### Quality of life

**Figure 5.**
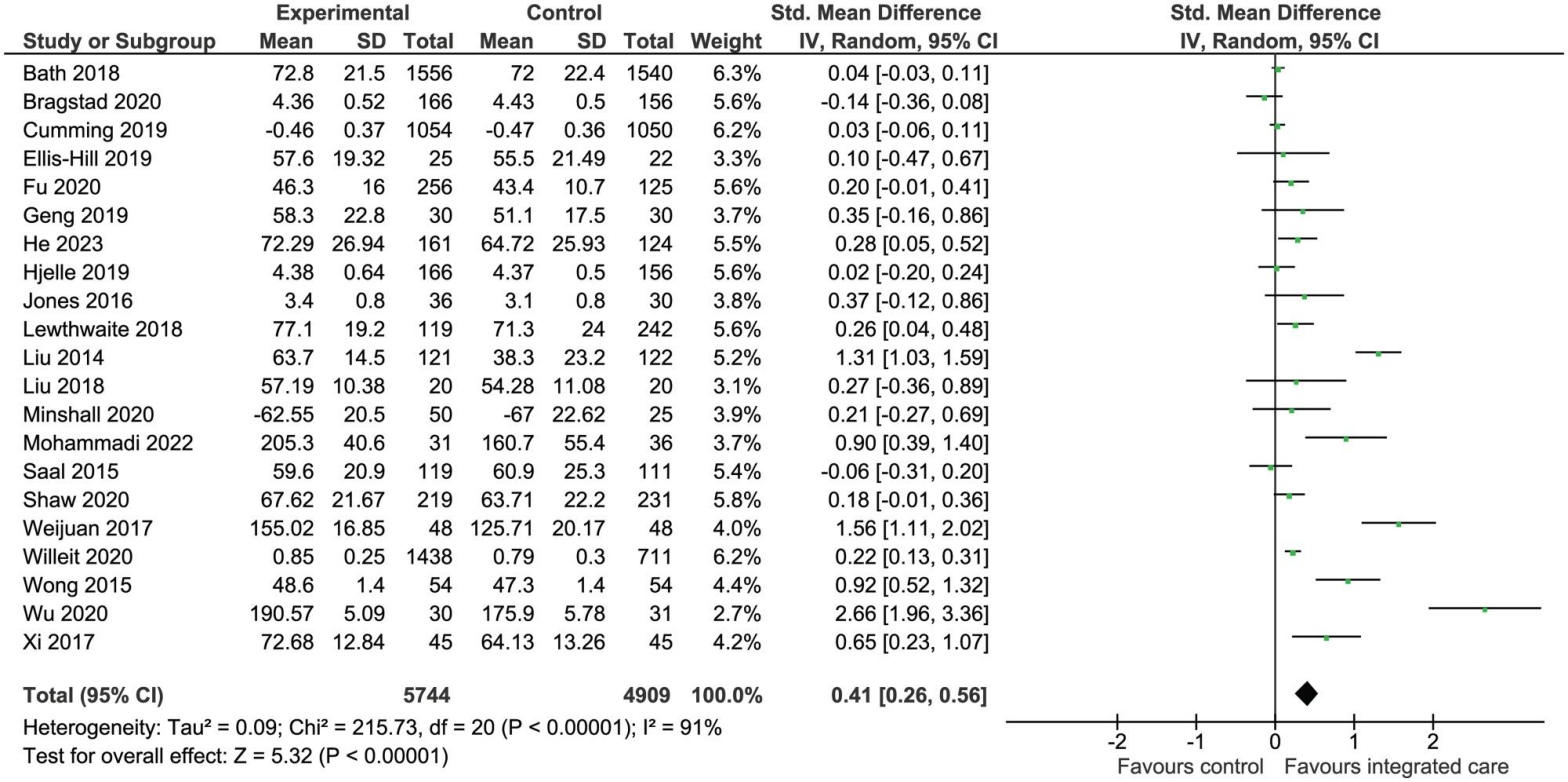
Comparison of integrated care with control for quality of life (randomized controlled trials).

Meta-analysis was conducted on a total of 21 studies (*n* = 10 653) using SMD to combine different quality of life (QoL) scales (Figure 5). Overall, there were significant improvements in QoL in favour of integrated care (SMD = 0.41, 95% CI: 0.26–0.56, *P* < 0.00001, *I*^2^ = 91%). Subgroup meta-analysis showed that the greatest differences in QoL were observed with SF-36 and SSQOL (Supplementary Material S4).

#### Unplanned readmission

**Figure 6.**
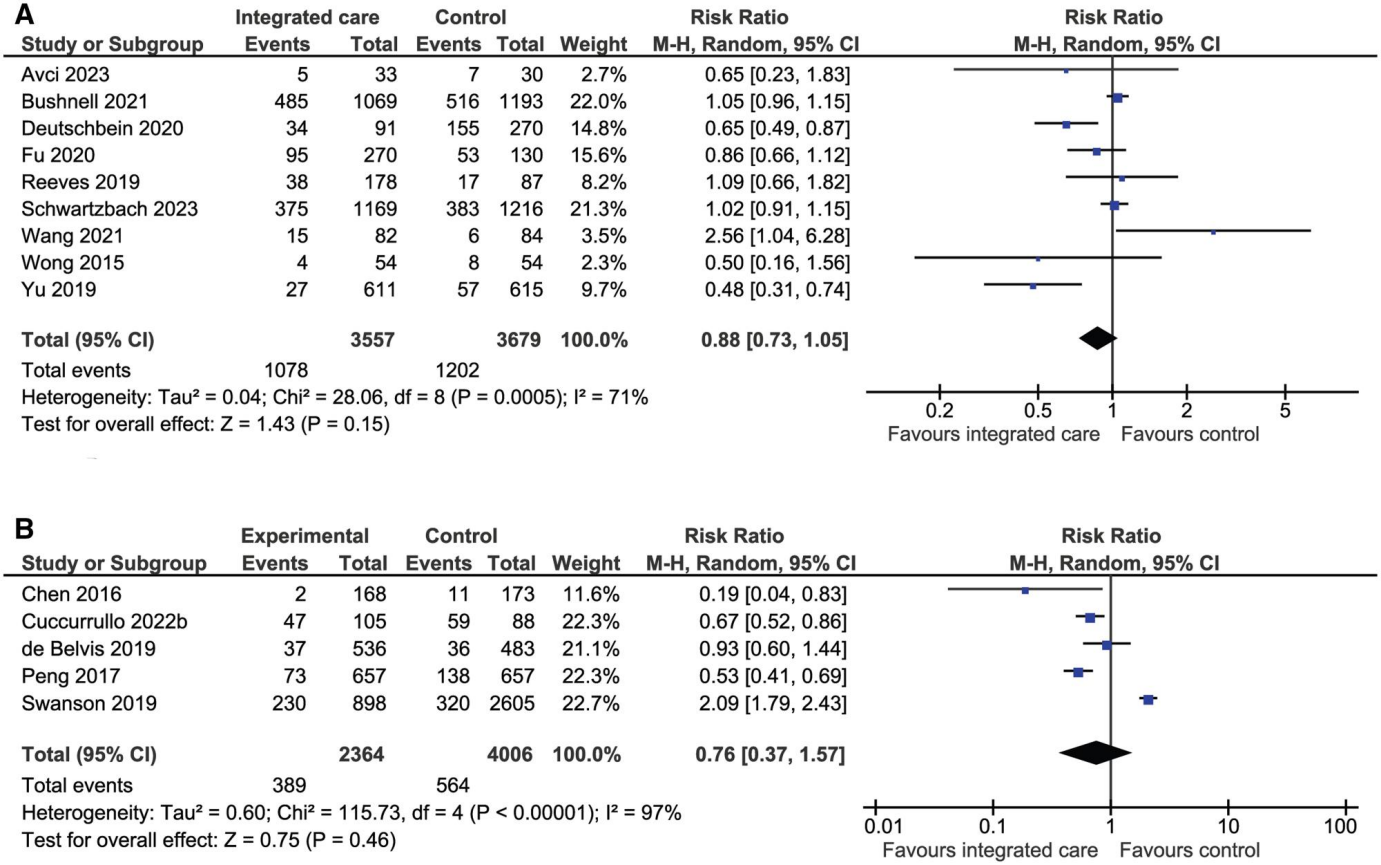
Comparison of integrated care with control for unplanned readmission (A, randomized controlled trials; B, nonrandomized controlled trials).

There was no significant difference in unplanned (based on the assumption that the majority of readmissions were due to complications or events such as subsequent stroke or myocardial infarction [MI] that could not be predicted) readmission rates in nine (*n* = 7236) RCTs (RR 0.88, 95% CI: 0.73–1.05, *P* = 0.15, *I*^2^ = 71%, Figure 6A) or in five (*n* = 6370) NRCTs (RR 0.76, 95% CI: 0.37–1.57, *P* = 0.46, *I*^2^ = 97%, Figure 6B).

#### Anxiety and depression

**Figure 7.**
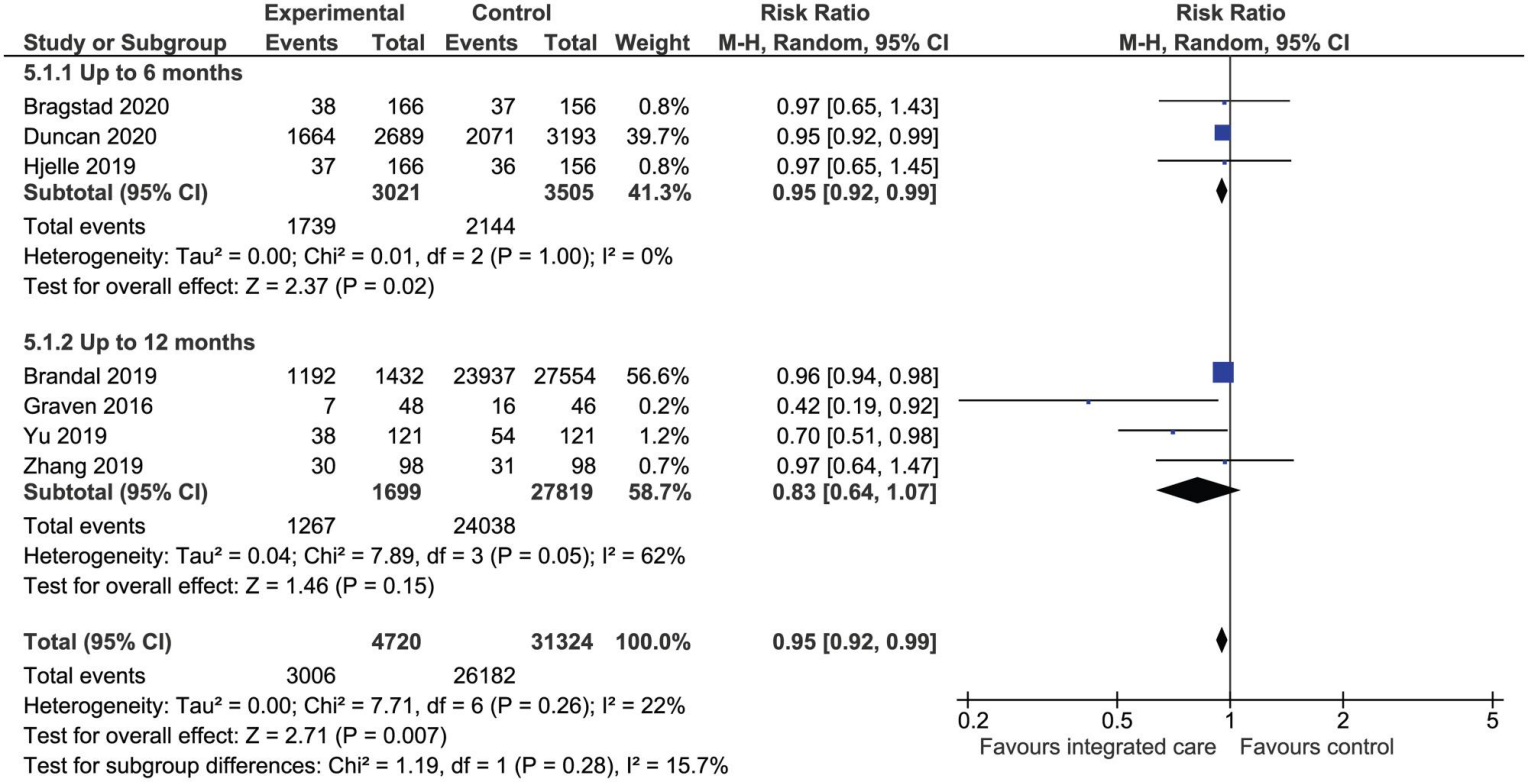
Comparison of integrated care with control for proportion of patients with depression by follow-up time.

Meta-analysis was conducted on seven (*n* = 36 044) RCTs reporting proportions of patients with depression using Yale-Brown, GDS, PROMS, PHQ2 and HADS-D scales (Figure 7). Integrated care was associated with a significant reduction in the proportion of patients with depression (RR 0.95, 95% CI: 0.92–0.99, *P* = 0.007, *I*^2^ = 22%, Figure 7) regardless of follow-up time (up to 6 months or up to 12 months). Meta-analysis also showed that anxiety and depression scores were significantly lower with integrated care regardless of scale or follow-up time (Supplementary Material S4).

#### Lifestyle and cardiovascular risk factors

Overall, integrated care was associated with a significant improvement in cardiovascular risk (Table 1). Further information can be found in Supplementary Material S4.

#### Adherence to intervention

Overall, adherence rates were higher with integrated care but the difference was not statistically significant (*P* = 0.32, Table 1). Further information can be found in Supplementary Material S4.

#### Exploratory analysis: functional status

**Figure 8.**
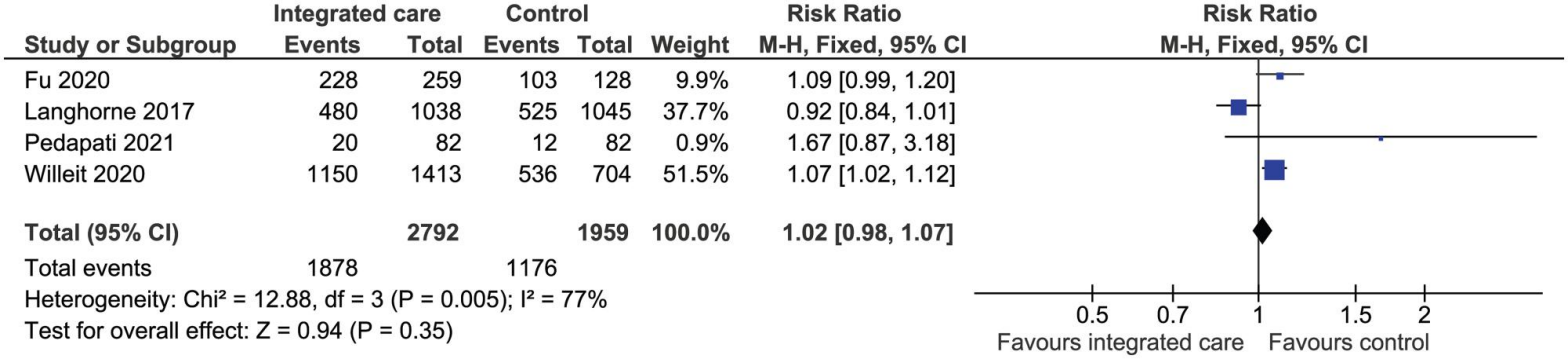
Comparison of integrated care with control for proportion of patients with favourable outcome (mRS ≤ 2).

An exploratory meta-analysis was conducted on four RCTs (*n* = 4751) that included data on functional status according to the modified Rankin Scale (mRS). This analysis showed no significant difference in favourable outcome (defined as mRS ≤ 2) between integrated care and control (RR 1.02, 95% CI: 0.98–1.07, *P* = 0.35, *I*^2^ = 77%, Figure 8).

#### Exploratory analysis: recurrent risk of cardiovascular disease

Meta-analysis was conducted for MI (three studies, *n* = 7733) and vascular death events (two studies, *n* = 4221). Although there were fewer events associated with integrated care, these differences were not statistically significant (Table 1). Further information can be found in Supplementary Material S4.

## Discussion

To our knowledge, this is the first comprehensive systematic review with meta-analyses of integrated care in stroke showing that integrated care was associated with improvements in QoL, reductions in recurrent stroke, anxiety and depression and positive benefits on some cardiovascular risk factors but did not impact mortality, major bleeding or readmission rates. Other systematic reviews have evaluated interventions such as organized inpatient stroke care,^37^ exercise,^38–41^ early supported discharge^42–44^ and interventions for cognitive rehabilitation,^45^ depression^46^ and anxiety^47^ however none of these reviews were conducted in the setting of integrated care.

Whilst some studies demonstrated a benefit in outcomes with integrated care interventions, other studies using similar interventions did not. These observations highlight a lack of consensus over which components should form part of an integrated stroke pathway and how they should be implemented. Indeed, individual studies demonstrated that interventions such as exercise, rehabilitation, risk factor management and mental health initiatives were most effective at improving outcomes in people with stroke. These included studies of cardiovascular exercise and high-intensity rehabilitation that were associated with significantly lower mortality rates.^25^^,^^35^^,^^48^ The EXPRESS study of rapid assessment and treatment initiation with high-dose aspirin or clopidogrel and intensive and regular follow-up demonstrated a reduction in stroke risk and disability (mRS ≥ 2) even after 10 years.^49^ Similarly, the COMPASS study of combined anticoagulant and antiplatelet therapy significantly reduced the risk of ischaemic/unknown stroke in 1032 patients with prior stroke (HR, 0.33, 95% CI: 0.14–0.77, *P* = 0.01).^50^ The STROKE-CARD study, a comprehensive disease management program delivered by a multidisciplinary team that included risk factor management and patient education, reported reduced cardiovascular risk and improved QoL.^51^ Studies employing interventions such as early supported discharge, regular community follow-up, education, cognitive and motivational training, and goal setting reported significant reductions in the proportions of participants with depression or anxiety and improvements in QoL,^30^^,^^36^^,^^52–57^ with individualized care pathways and mhealth technology providing substantial improvements in these outcomes.^20^^,^^55^^,^^58–60^

Conversely, there are large and well-designed randomized trials of comprehensive integrated care interventions that did not demonstrate any substantial benefit over standard care. The structured ambulatory post-stroke care program (SANO) study, a 1-year patient-centred integrated care intervention including regular follow-up, lifestyle advice, goal setting and motivational interviewing, demonstrated positive benefits in controlling CV risk factors such as blood pressure and LDL cholesterol, but did not reduce the rate of major cardiovascular events.^61^ In the Triple Antiplatelets for Reducing Dependency after Ischaemic Stroke (TARDIS) study, significantly more bleeding events were reported overall in the TARDIS group (19.8%) compared with the control group (9.1%, *P* < 0.001), prompting the study to be stopped early.^62^ The Very Early Rehabilitation Trial for stroke study (AVERT) showed significantly fewer participants in the AVERT group had favourable outcome and there was no difference in mortality or QoL.^63–65^

Overall, the results of this systematic review are inconsistent, with 34% of reviewed studies showing no benefit over comparator treatment and substantial heterogeneity between studies.

The centralization of stroke services and the creation of HASUs has improved outcomes for patients compared with traditional stroke units, reflected in reduced mortality rates and hospital length of stay^6^^,^^7^ however there is currently no standard HASU care pathway and outcomes vary widely as a result.^66^^,^^67^ Much of the published data on stroke care management is in the acute setting,^68^ where integrated care plans such as early supported discharge^43^^,^^44^^,^^53^^,^^69^^,^^70^ are often clearly defined however less is known about patient care following discharge back into the community and studies show that care from this point onwards is often discontinuous,^71–73^ which can result in unmet needs and increased potential for hospitalization and institutionalization.^72^ A recent Sentinel Stroke National Audit Programme (SSNAP) annual report identified a decline in the proportion of patients receiving a 6-month review and highlighted the need for an adequately resourced multidisciplinary team of healthcare professionals required to deliver the recommended amount of rehabilitation of patients according to their need, especially in the community setting.^74^

### Primary outcomes

Meta-analysis showed no difference overall in mortality between integrated care and control or standard care. Although subgroup analysis showed a significant reduction in long-term mortality in favour of integrated care, there were only three studies in this subgroup and the findings must be interpreted with caution. The overall findings are consistent with the published literature with systematic reviews of early supported discharge and exercise showing no significant differences in mortality rates compared with standard care.^43^^,^^44^^,^^75^

A statistically significant reduction in recurrent stroke in favour of integrated care was observed with RCTs but not with NRCTs. Few systematic reviews have directly assessed the impact of interventions on recurrent stroke as an outcome however the interventions in this analysis that were associated with reductions in recurrent stroke (lifestyle and cardiovascular risk factor management) have also been shown to reduce recurrent stroke rates in meta-analyses.^76–78^

Integrated care was associated with more major bleeding events compared with standard care however the difference was not statistically significant (*P* = 0.50). There are numerous meta-analyses comparing oral anticoagulants for stroke prevention in patients with atrial fibrillation (AF) and in general direct oral anticoagulants (DOAC) have a lower bleeding risk than Vitamin K agonists (VKA).^79–82^ Combination treatment with anticoagulant and antiplatelets is associated with increases in the risk of major bleeding in cardiovascular disease (CVD).^83^^,^^84^ In this review, the TARDIS and COMPASS studies reported increased major bleeding events with triple therapy and reduced events with low-dose rivaroxaban and aspirin treatment, respectively.^50^^,^^62^ Meta-analyses of major bleeding risk in stroke survivors are currently lacking in the published literature.

### Secondary outcomes

Overall, integrated care was associated with improvements in QoL and the magnitude of improvement was greatest with the SF-36 and SSQOL assessment tools however these findings must be interpreted in the presence of substantial (>75%) heterogeneity between studies. Meta-analysis also showed that integrated care was associated with statistically significant reductions in the proportion of patients with anxiety and depression, with a statistically significant benefit for the cardiovascular risk factors of systolic blood pressure (SBP) and LDL cholesterol.

Improvements in QoL are documented in the literature with interventions such as organized stroke care^37^ and exercise^75^^,^^85^^,^^86^ however the degree of improvement in QoL is difficult to define due to lack of information.^75^

### Limitations

There is currently no standard or unifying definition of integrated care and delivery is likely to be influenced by views and expectations of various stakeholders in the health system,^4^ an observation that has been confirmed in studies of hyperacute stroke care where different outcomes were reported due to differences in care models and priorities.^66^^,^^67^ For example, centralization of stroke services in London resulted in a significant reduction in mortality rates since almost all London patients were treated in a HASU and were more likely to receive evidence-based care whereas stroke patients in Manchester were far less likely to be treated in a HASU and receive evidence-based care, resulting in unchanged mortality rates.^66^ The WHO reports large variations in the definition of integrated care and for the purposes of this review, we used a definition of integrated care developed by the NHS and WHO (Supplementary Material S1) and included studies where there was evidence of an integrated care approach according to this definition however some studies could have been missed during the literature searches if they were not considered to be integrated care per definition. Due to the volume of published studies in stroke, the search was restricted to 12 years however it is possible that some important studies could have been missed if they fell outside of the search window. Most of the integrated care trials in this review included multiple interventions or comprised complete pathways and the effect of individual interventions could not be assessed.

Meta-analysis of outcomes indicates high heterogeneity between studies, in particular those evaluating QoL, which confounds interpretation of the findings. Due to the majority of studies having small patient numbers and the paucity of data available for some outcomes, the systematic review did not exclude studies considered to be at moderate or high risk of bias and these studies may have influenced the results of the meta-analyses.

## Conclusions

The findings of this systematic review demonstrate that integrated care improves quality of life, reduces recurrent stroke, anxiety and depression and is associated with a positive benefit on some cardiovascular risk factors but does not impact mortality or readmission rates. Further research is necessary to fully determine which elements of integrated care provide the most benefit to people with stroke.

## Supplementary Material

hcaf029_Supplementary_Data

## Supplementary material

*Conflict of interest:* G.Y.H.L. is a consultant and speaker for BMS/Pfizer, Boehringer Ingelheim, Daiichi-Sankyo, Anthos. No fees are received personally. He is a National Institute for Health and Care Research (NIHR) Senior Investigator and co-principal investigator of the AFFIRMO project on multimorbidity in AF, which has received funding from the European Union’s Horizon 2020 research and innovation programme under grant Agreement No. 899871. B.J.R.B. has received research grants from BMS/Pfizer. I.D.J. has received investigator-initiated funds from Bristol Myers Squibb and Astra Zeneca. No fees are received personally. A.M.H., K.H., I.E., I.K., K.L.H. and M.F-R. have no conflicts of interest to declare.
