# Supplementary material for: Integrated care management for patients following acute stroke: a systematic review
Source: QJM. 2025 Jan 24;118(5):317–28. doi: 10.1093/qjmed/hcaf029 (PMC12341757; doi:10.1093/qjmed/hcaf029)
Supplement: hcaf029_Supplementary_Data [file hcaf029_supplementary_data.zip › hcaf029_Supplementary_Data/Supplement S2.docx]

# Supplement S2. Characteristics of included studies

Table 1: Summary of study characteristics including study design, patients, interventions, outcome measures, results and conclusions

| **Study details** | **Design/Patients** | **Intervention** | **Outcomes** | **Results** | **Conclusions** |
| --- | --- | --- | --- | --- | --- |
| Abdul Aziz, 2020 (1) | Pragmatic cluster randomised controlled trial-within trial at public primary care health centres  Control n=65, iCaPPS n=86 | Screening for complications, QoL, depression | QoL (primary) EQ-5D-5L at 6 months  Cost/QALY (secondary) | 6-month EQ-5D-5L score changes:  Improvement: Intervention 36.1%, control 23.1%  No change: Intervention 39.5%, control 69.2%  Deterioration: Intervention 24.4%, control 7.7%  Cost/QALY  Intervention USD469.60 Control USD538.41 | The iCaPPS© is a very cost effective method for monitoring post stroke patients who are residing at home |
| Ahmadi et al, 2020 (INSPiRE-TMS) (2) | Prospective, randomised, open­label, blinded endpoint, event-­driven trial  Control n=1042, intervention n=1030 | Counselling, lifestyle changes, medication adherence | Composite of vascular events, ACS, vascular death (primary)  Secondary: each component of primary, readmission, recurrent stroke, major bleeding | Vascular mortality (ITT): Intervention n=30 (2.9%), control n= 32 (3.1%) HR 0·94 (0·57–1·54)  All-cause mortality (ITT): Intervention n=73 (7·1%), control n=85 (8·2%) HR 0·85 (0·62–1·17)  Stroke (ITT): 11.8% intervention, 11.4% control (HR 1·02, 95% CI: 0·79–1·32) | Provision of an intensified secondary prevention programme in patients with non-disabling stroke or transient ischaemic attack was associated with improved achievement of secondary prevention targets but did not lead to a significantly lower rate of major vascular events |
| Avci and Gozum, 2023 (3) | Parallel-group, assessor-blind, monocenter randomized controlled trial  Control n = 60 (30 patients and 30 caregivers), Intervention n = 66 in (33 patients and 33 caregivers) | Interviews and education (web-based training) for caregivers and patients | Readmission rates | Hospital return status of stroke patients:  Intervention Group (n = 33): Yes = 5 (15.2%), no = 28 (84.8%)  Control Group (n = 30): Yes = 7 (23.3%), no = 23 (76.7%) | Interventions based on the transitional care management (TCM) increased caregiver competence, prepared them for caregiving and e-health literacy, and prevented the progression of burnout. Transitional Care Model–based interventions also improved patient outcomes. Readmission and pressure ulcers were less common in stroke patients in the intervention group compared with the control group. |
| Bath et al., 2018 (TARDIS) (4) | International (UK, Denmark, Georgia, NZ) prospective randomised open-label blinded end-point superiority clinical trial  Guideline (control) n=1540, Intervention n=1556 | Intensive treatment group given combined aspirin (load 300mg, maintenance 50-150mg daily, typically 75mg), clopidogrel (load 300mg, maintenance 75mg daily), and dipyridamole (200mg twice daily modified release, given orally; or 100mg three or four times daily). Guideline treatment received combined aspirin and dipyridamole or clopidogrel alone, using the loading and maintenance doses; these treatments were given for 30 days, after which routine treatment was taken. | Primary: Incidence and severity of any recurrent stroke (ischaemic, haemorrhagic; assessed using the modified Rankin Scale) or TIA within 90 days by blinded telephone follow-up.  Secondary: bleeding and its severity, death, myocardial infarction, disability, mood, cognition, QoL | The incidence and severity of recurrent stroke or TIA did not differ between intensive and guideline therapy [adjusted common odds ratio (acOR) 0.90, 95% confidence interval (CI) 0.67 to 1.20; p = 0.47]. Major (encompassing fatal) bleeding increased with intensive as compared with guideline therapy (adjusted hazard ratio (aHR) 2.23, 95% CI 1.25 to 3.96; p = 0.006) and the difference only developed during the active treatment phase. Quality of life and mood did not differ between the treatment groups. | The TARDIS trial found that there was no significant reduction in the recurrence of stroke or TIA, or their severity, with intensive antiplatelet therapy based on three agents as compared with guideline therapy. However, triple antiplatelet therapy was associated with increased major bleeding. Overall, there was no effect on the net balance between harm and benefit confirming the overall neutral finding of the trial. |
| Bergh, 2023 (5) | Repeated measures analysis (pre-post) from a retrospective cohort of patients registered in the Norwegian Stroke Register (NSR).  N = 11009 included in study comparing functional outcome (5388 in 2017 and 5621 in 2019) | Standardised Care Pathway for stroke (SCP) based on National guidelines | Primary: change in mRS at 90 days  Secondary: Mortality at 90 days | No significant in change in mRS between the 2 years (OR=1.06, 95%CI 0.86, 1.30).  Overall, 1407 (16%) of 8785 patients had died within 90 days in 2017, and 1380 (15.3%) of 9028 in 2019, no significant difference between groups. | No significant improvement in function outcome after the introduction of an SCP. However, significantly more patients were discharged directly home from hospital, and more patients with stroke were treated in a dedicated stroke unit after the implementation (in 2019). |
| Bernhardt et al., 2015 (AVERT) (6) | Single-blind randomised controlled trial in 56 acute stroke units in 5 countries (Aus, NZ, Malaysia, Singapore, UK -Eng, Scot, Wales, NI)  Control n=1050, intervention n=1054 | Very early mobilisation (exercise and movement therapy) | Primary: favourable outcome (mRS)  Secondary: Change in mRS, mortality | Favourable outcome: intervention 480 (46%), control 525 (50%) (adjusted OR 0.73 [95% CI: 0.59-0.90]), p=0.004  Mortality: Intervention 88/1048 (8%), control 72/1050 (7%), OR 1.34 (95% CI: 0.93-1.93), p=0.113 | Significantly fewer participants in the AVERT group had favourable outcome and there was no difference in mortality |
| Bodechtel et al., 2016 (Stroke East Saxony [SOS] care project) (7) | Retrospective database case-control study  Control: n=45, Intervention n=45 | Intervention: Post-stroke care pathway managed by case manager  Control: usual care that was provided by primary care physicians after discharge | Functional independence, recurrent stroke, mortality, QoL, risk factors | The following target goals were more frequently achieved in SOS Care patients than in controls: blood pressure (100% vs. 46.2%, P < 0.001), cholesterol (100% vs. 74.4%, P < 0.001), and body mass index (67.4% vs. 46.2%, P = 0.052).  More patients in the intervention group were functionally independent at 12 months after the index event than patients receiving only usual care (75.6% vs. 48.9%; P = 0.009).  Quality of life measured by the EQ5D questionnaire was rated significantly higher in SOS Care patients than in controls (median index: 0.887 [IQR, 0.099] vs. 0.788 [0.0625]; P = 0.049). | Pilot data suggest that organized post-stroke care enhances achievement of secondary prevention goals |
| Bragstad et al., 2020 (8) | Multicenter, prospective, randomized, assessor-blinded, controlled trial with two parallel groups  Gave consent n=353, randomised n=322  Control n=156, intervention n=166 | Dialogue-based intervention to promote psychosocial wellbeing (psychological) | Primary: psychosocial wellbeing  Secondary: HRQoL, depression | Between-group differences at 12 months (mean, SE):  Physical domain 0.06 (−0.07, 0.20), P=0.36  Communication domain 0.06 (−0.05, 0.16), P=0.27  Psychosocial domain 0.07 (−0.11, 0.25), P=0.45 | The dialogue-based intervention implemented in this RCT did not lead to lower levels of emotional distress and anxiety at 12 months post-stroke. The intervention did not lead to higher levels of health-related quality of life or higher sense of coherence at 12 months post-stroke. Based on the outcome measures used in this study, there is insufficient evidence to support implementation of the intervention in its current form. |
| Brandal, 2019 (9) | Prospective, randomised, open­label, blinded endpoint, event-­driven trial  N=99833 (Control n=27554, intervention n=1432) | Early supported discharge (ESD) | Primary: Satisfaction with rehab after discharge  Secondary: Depression | Significantly improved satisfaction with rehab after discharge (OR: 1,78, 95% CI:1.17-2.49), independence in mobility (OR: 1.50, 95% CI: 1.17-1.92), toileting (OR: 1.30, 95% CI: 1.06-1.61) and dressing (OR: 1.23, 95% CI: 1.02-1.48), significantly less dysthymia/depression (OR: 0.68, 95% CI: 0.55-0.84) | Patients that received ESD after stroke were more satisfied with rehabilitation after discharge and experienced less depression than patients that received other health care and/or rehabilitation care |
| Brouns, 2021 (10) | Pre-post test, controlled, pragmatic clinical trial  Control n=258, intervention n=310 | Web-based e-rehabilitation comprising physical exercise and cognitive exercise (psychological) programmes (mhealth) | Primary: level of disability (SIS)  Secondary: QoL EQ-5D-5L at 3 and 6 months | Primary: Between T3 and T6, the change in scores for the SIS subscales Communication (control group/ intervention group –1.7/–0.3) and Physical strength (–5.7/3.3) were significantly greater in the total intervention group (all mean differences< minimally clinically important differences). No significant differences were found for other SIS subscales or secondary outcomes, or between T0–T3 and T0–T6.  Secondary: Within group EQ-5D-5L changes (mean, SD):  3 months Intervention 0.07 (0.2), control 0.07 (0.2), between group P=0.19  6 months Intervention 0.01 (0.2), control –0.01 (0.2), between group P=0.38  LMM (0-6 months) P-value 0.50 (95% CI: 0.0–0.1) | eRehabilitation alongside conventional stroke rehabilitation had a small positive effect on communication and physical strength on the longer term, compared to conventional rehabilitation only  No difference in QoL at 3 or 6 months |
| Brouwer et al., 2018 (11) | Multicenter randomized controlled trial with 2 groups: an intervention (“tune-up”) group and a control group having the same exposure to assessment  N=132 (Control n=52, Intervention n=51) | Patient-centred treatment sessions comprising strength/power, balance, cardiovascular endurance (exercise), motor coordination, and education about relevant community resources, as well as how to monitor their mobility and activities | Primary: community reintegration  Secondary: QoL (SF-36) at 12 months | At 12 months, both groups showed significant improvement in community reintegration (P<.05), a trend evident at all time points, with no difference between groups (mean difference, -0.5; 95% confidence interval, -1.8 to 2.7; P=0.68). Similarly, a main effect of time reflected improvement in mobility-related and quality of life outcomes for both groups (P≥.0.5), but no group differences (P≥0.30). | All participants in the tune-up group met or exceeded at least 1 mobility-related goal; however, the intervention did not differentially improve community reintegration. The improvements in mobility and quality of life over the 15-month postdischarge period may be secondary to high activity levels in both study groups and exposure to regular assessment. |
| Buckley et al., 2022 (12) | Retrospective observational study  N=836,923  Propensity matched cohorts:  Control n=2909, intervention n=2909 | Exercise-based cardiac rehabilitation | 2-year mortality, recurrent stroke, rehospitalisation | Mortality: 8.5% Intervention, 16.5% control, (OR 0.47, 95% CI 0.40-0.56)  Recurrent stroke: 39.2% intervention, 42,3% control (OR 0.88, 95% CI 0.79-0.98)  Rehospitalisation at 2-years was proportionally lower with 40.7% (n=1,185 of 2,909 patients) in the exercise-based cardiac rehabilitation cohort compared to 51.8% (n=1,507 of 2,909 patients) in the controls (OR 0.64, 0.58-0.71) | Exercise-based cardiac rehabilitation prescribed for people following a stroke associated with significantly lower odds of major adverse cardiovascular events at 2-years, compared to usual care |
| Bushnell et al., 2021 (13) | COMPASS was a cluster-randomized trial of the effectiveness of the COMPASS-TC intervention (INV) compared with usual care (UC)  Participating hospitals: control n=20, inv n=20 Patients: Control n=1193, inv n=1069 | Post-acute care pathway (full pathway) follow-up with the advanced practice provider and nurse coordinator in clinic within 7 to 14 days post discharge, an individualized electronic care plan, and referral to community services | 1-year mortality, 30- and 90-day unplanned readmissions, 1 year all-cause readmissions | Mortality: 8.5% intervention, 8.8% control (OR, 0.93; 95% CI, 0.68–1.26)  Rehospitalisation: 30d admissions: Intervention 105 (9.9%), control 103 (8.7%), OR 1,20 (95% CI: 0.83-1.74)  60d readmissions: Intervention 210 (19.9%), control 222 (18.9%), OR 1.10 (95% CI: 0.84-1.41)  1 year all-cause readmissions: Intervention 485 (45.4%), control 516 (43.3%), HR 1.06 (95% CI: 0.95-1.77)  1 year stroke readmission: Intervention 67 (6.3%), control 62 (5.2%), HR 1.26 (95% CI: 0o.0-1.77) | The risk of unplanned all-cause readmission was similar between INV versus UC at 30 (9.9% versus 8.7%) and 90 days (19.9% versus 18.9%), respectively. No significant differences between randomization groups were seen in 1-year all-cause readmissions, stroke-specific readmissions, or mortality |
| Cecchi et al., 2020 (14) | Prospective observational study  Control n=443; intervention n=84 | Evidence-based stroke rehabilitation integrated care pathway (ICP) | Mortality | Mortality: 2015-17: 4 (0.9%), 2018: 1 (1.2%), p=0.803 | Compared to prior practice, ICP implementation was associated to a significant improvement in some patient-oriented outcomes at discharge: recovery of trunk control, bladder catheter removal, and pressure sore resolution. However, there was no difference in mortality |
| Chang et al., 2018 (15) | Retrospective cohort study using National Health Insurance database  Control n=2491, intervention 2103 | High intensity rehabilitation (inpatient, outpatient or both) versus no rehab | All-cause mortality | Model 1  Control: 18.4%, low-intensity 17.9%, high intensity 14.6% (p=0.0062)  Model 2  Control: 18.4%, inpatient + outpatient 10.2%, inpatient only 25.2%, outpatient only 12.6% (p<0.0001) | Rehabilitation use was associated with reduction of readmissions/mortality risks following stroke or TIA. |
| Chang et al., 2022 (16) | Multi-centre cohort study  Control n=212, intervention n=122 | Post acute care (PAC) program | Quality of Life (EQ-5D) | EQ5D  Non-PAC: before 0.03±0.32, after 0.20±0.32, P-value <0.001  PAC: before 0.15±0.35, after 0.38±0.32, P-value <0.001 | Traditional inpatient post-stroke rehabilitation and PAC programs are both helpful for post-stroke functional recovery. Compared to traditional inpatient rehabilitation, the PAC program showed significantly more improvement in basic and instrumental activities of daily living, nutrition, quality of life, and cognition. |
| Chen et al., 2016 (17) | Quasi-experimental controlled study  Control n=173, intervention n=168 | Individualised transitional care model based at home | Adherence to medication, readmission | Medication compliance rates: intervention 161 (95.83%) vs. control 92 (53.18%), p = 0.004  Readmission: Intervention 2 (1.19%) vs. control 11 (6.36%), Chi^2^ = 5.30, p = 0.02) | The intervention was associated with significantly higher medication compliance and satisfaction with acute hospitalisation and lower readmission rates. |
| Chen, 2017 (18) | Retrospective cohort study  N=11,419  Propensity matched group n=390 (RR n=130, NtR n=130, NoR n=130) | Rehabilitation for hospitalised patients including mobilisation (exercise), speech therapy and dysphagia treatment | 5-year all-cause mortality | Rehab + rehab ward (RR) 17.7% Rehab no rehab ward (NtR) 26.9% (HR 2.2 95% CI 1.36-3.57; P<.01 vs RR) No rehab (NR) 47.7% (HR, 4.00; 95% CI, 2.55-6.27; P<.01 vs RR) | Among first-time stroke survivors, the 5-year mortality HR reported in this study indicates that subjects who underwent rehabilitation in the rehabilitation ward had 2.2 times lower mortality risk than those who received rehabilitation without transfer to the rehabilitation ward, and had 4 times lower mortality risk than those who did not receive rehabilitation |
| Cheng, 2018 (19) | Randomised controlled trial  N=201 (CRT n=84, control n=84) | Patient and family member education, cognitive training (psychological), rehabilitation training, regular follow-up | HADS anxiety/depression score  SAS anxiety score, SDS depression score | Both montreal cognitive assessment score change (Month12 [M12]-baseline; P = .001) and minimum mental state examination score change (M12-baseline) were higher in CRT group than that in control group (P = .004), and the percentage of cognitive impairment by montreal cognitive assessment score ≤26 was lower (P = .003) in CRT group compared to control group at month 12.  HADS anxiety score change at 12 months: -0.23 ± 0.20 (CRT) vs 0.62 ± 0.17 (control), p=0.002  SAS anxiety score change at 12 months: -0.23 ± 0.20 (CRT) vs 2.00 ± 0.77 (control), p=0.006  HADS depression score change at 12 months: -0.71 ± 0.23 (CRT) vs 0.44 ± 0.18 (control), p<0.001  SDS depression score change at 12 months: -2.10 ± 1.10 (CRT) vs 2.30 ± 0.88 (control), p=0.002 | CRT contributes to the recovery of cognitive impairment, and decreases anxiety and depression in poststroke patients |
| Cheng et al., 2018 (20) | Randomised controlled trial  N=407 (Control n=200, int n=207) | Medication modification relevant to stroke risk factors, tobacco cessation, physical activity, depression, and medication adherence | Change in SBP, LDL cholesterol, Physical activity, smoking cessation, 10-year risk of CV events | Differences in Changes From Baseline Between Control and Intervention Arms* (95% CI):  SBP −3.6 mm Hg (−9.3 to 2.2) p=0.55  LDL mg/dL, mean (SD) −10.2 mg/dL (−21.1 to −0.6) p=0.03  Smoking cessation 0.6 OR (0.3 to 1.3), p=0.58  Physical activity assessed as exercise ≥3 d per wk, n (%):0.9 OR (0.5 to 1.8), p=0.84  10 year risk of CV events: %, mean (SD) -0.8 (−3.1 to 1.6), p=0.98  *Repeated-measures mixed-effect models included baseline, 3-mo, and 12-mo values. | This intervention did not improve SBP control beyond that attained in usual care among vulnerable stroke survivors. A community-centered component could strengthen the intervention impact. |
| Chiu, 2021 (21) | Large-scale prospective cohort study  N=1786 (PAC n=273, non-PAC n=273) | Post-acute care (PAC): physical exercise (physical, occupational, or speech-swallowing therapy) in multiple sessions | Functional disability, cognitive status, functional status, QoL, functional balance | From 12 weeks after rehabilitation to 1 year after rehabilitation, the PAC group had higher total scores for BI, FOIS, IADL, BBS, and MMSE compared to the non-PAC group. That is, the PAC group had larger improvements in all functional status measures except EQ-5D. According to the DID values (with mean and standard errors) for the PAC group and non-PAC group, the PAC program had significant (p < 0.001) positive net effects on BI, FOIS, IADL, BBS, and MMSE but had a significant (p < 0.001) negative net effect on EQ-5D during the study period. | Although the incidence of stroke is high, PAC provided early after stroke can improve restoration of function. |
| Chu, 2020 (22) | Single-centre randomised controlled trial  N=61 (Control n=30, intervention n=31) | Training for caregivers (education), personalised training plan for patients for recovery of physical function (mobility, self-care, bowel/toilet control) | Primary: Physical function  Secondary: QoL EQ-5D at 6 months | Compared with that in the control group, BI increased more at 3 months and decreased less at 6 months in the intervention group, there was a signiﬁcant difference in mean BI scores across the three time points (F = 21.96, p = 0.0001), but no signiﬁcant between-group difference (F = 0.94, p = 0.3371). In the intervention group, BI scores at 3-and 6-months post-discharge were higher than that before discharge (t = 8.38, p = 0.0001; t = 4.14, p = 0.0003). In the control group, BI scores at 3 months were higher than that before discharge (t = 5.29, p = 0.0001), but no signiﬁcant difference at 6 months. At 6 months post-discharge, the intervention group and the control group had similar EQ-5D scores (p = 0.91), and similar CBI scores (3.67 vs 3.68, p = 0.98). | The study showed that the novel nurse-trained, family member-delivered rehabilitation model improved physical recovery indicated by BI scores without increasing caregiver burden, compared to usual care, for rural stroke patients in southwest China. |
| Cuccurrullo et al., 2022 (23) | Prospective matched cohort study  Control n=88, intervention n=105 | SRP program: Physician Visits, Outpatient Therapy, and Modified Cardiac Rehabilitation (management of blood pressure (BP), cholesterol control, reduction of blood sugar, getting active, eating better, losing weight, and smoking cessation) Lifestyle | Readmission rates | All cause readmission: control 59 (67%), intervention 47 (45%), difference in proportion 22%, HR 0.51 (0.31-0.83), p=0.006 | Acute care hospital readmissions were reduced in stroke survivors who participated in SRP. |
| Cuccurrullo et al., 2022 (24) | Prospective matched cohort study: feasibility study followed by matched group subanalysis  Control n=66, intervention n=76 | Enhanced SRP program including home visits, outpatient therapy and modifies cardiac rehabilitation | 1-year mortality | Mortality: Control 10/66 (15.2%), intervention 1/76 (1.3%), difference 13.9%, HR 0.11 (95% CI: 0.01-0.90), p=0.039 | Stroke survivors receiving a SRP integrating modified cardiac rehabilitation may potentially benefit from reductions in all-cause mortality and improvements in cardiovascular performance and function |
| Cuccurrullo, 2019 (25) | Prospective cohort study: feasibility study followed by matched group sub-analysis  N=783 (SRP feasibility study n=136, matched NP n=66, matched SRP n=76) | Physician Visits, Outpatient Therapy, and Modified Cardiac Rehabilitation (management of blood pressure (BP), cholesterol control, reduction of blood sugar, getting active, eating better, losing weight, and smoking cessation) Lifestyle | 1-year mortality | The feasibility study showed the SRP to have the following (a) excellent safety, (b) markedly low 1-yr poststroke mortality from hospital admission (1.47%) compared with national rate of 31%, (c) improved cardiovascular performance over 36 sessions (103% increase in metabolic equivalent of tasks times minutes), (d) improved function in Activity Measure of Post-Acute Care domains (P < 0.001), (e) positive re- views from SRP-participants/staff. Subgroup analysis showed the SRP to (a) positively impact mortality, nonparticipants had a 9.09 times higher hazard of mortality (P = 0.039), and (b) improve function in Activity Measure of Post-Acute Care domains (P < 0.001). | Stroke survivors receiving a SRP integrating modified cardiac rehabilitation may potentially benefit from reductions in all-cause mortality and improvements in cardiovascular performance and function |
| Cumming, 2019 (26) | International, multicenter (56 sites), pragmatic single-blind phase 3 randomized controlled trial, spanning 2006–2015  N=2104 (Intervention n=1054, control n=1050) | Very early mobilisation (physical activity within 24 hours) exercise | Primary: Good functional outcome  Secondary: QoL AQoL 4D at 12 months | No signiﬁcant diﬀerence in quality of life at 12 months between intervention (median 0.47, interquartile range [IQR] 0.07–0.81) and usual care (median 0.49, IQR 0.08–0.81) groups was identiﬁed (p = 0.86), nor were there any group diﬀerences across the 4 AQoL-4D domains. The same lack of group diﬀerence in quality of life was observed at 3 months. When cohort data were analyzed (both groups together), quality of life was strongly associated with acute length of stay, independence in activities of daily living, cognitive function, depressive symptoms, and anxiety symptoms (all p < 0.001). Quality of life in AVERT participants was substantially lower than population norms, and the gap increased with age. | Early and more frequent mobilisation after stroke does not have a marked inﬂuence on QOL over the subsequent year |
| de Belvis, 2019 (27) | Pre-post retrospective observational study  N=1,009  Control n=483, intervention n=526 | 5-stage clinical pathway: (1) initial framing; (2) evaluation/staging; (3) treatment; (4) rehabilitation; and (5) follow-up/monitoring (full pathway) | 30-day readmission and mortality | An increase context-sensitive in-patient numbers with more severe cerebrovascular events and an increase in patient transfers from the Stroke to Neurology Unit within three days (70 percent, p 0.25) were noted. Clinical pathway implementation led to an increase in patient flow from the Emergency Department to dedicated specialized wards such as the Stroke and Neurology Unit (23.7 percent, p < 0.001). Results revealed no statistically significant decrease in readmission rates within 30 days (5.7 percent, p=0.85) and no statistically significant differences in 30-day mortality. | Clinical pathway implementation showed an overall positive effect on patient management and service efficiency owing to the standardised application in time-dependent protocols and multidisciplinary/integrated care implementation, which improved all phases in acute ischemic stroke care |
| Deng 2020 (28) | A pilot randomized controlled trial  Control n=49, intervention n=49 | Multidisciplinary poststroke consultation team providing home visits, ongoing rehabilitation, medication reconciliation, self-management education | QoL | Mean values of Physical Components Summary and Mental Components Summary, integral components of Short-Form Health Survey-36, were significantly better in the intervention group at four and eight weeks (40.2 ± 6.3 and 42.9 ± 3.7 for the former; 43.9 ± 2.6 and 46.1 ± 1.8 for the later). The same trend was observed in Modified Barthel Index (87.1 ± 9.2 and 92.5 ± 6.7 at four and eight weeks, respectively). But the significant improvement in Caregiver Strain Index was only observed at four weeks. There were significant differences between groups in these scores. | The transitional care program has been proven to be feasible and improve health-related outcomes |
| Dello et al., 2021 (29) | Single-centre pre- and post-intervention study  Control n=238, intervention n=257 | Nurse-led management of temperature, glycaemia and swallowing (risk factors) | Mortality and functional status | The rates of 90-day death and dependency (mRS ≥2) were lower in the post-intervention period (51%) compared to the pre-intervention period (60%), adjusted OR 0.63, 95% CI: 0.41-0.97. | The intervention in the study was associated with significantly lower death and dependency compared with control |
| Deutschbein, 2020 (30) | Pragmatic non-randomised controlled trial  N=361 (intervention n=145, control n=270) | Individualised case management, with regular face-to-face visits as part of a comprehensive individualised programme (PT, OT, support required), regular monitoring and adjustment (full pathway) | Recurrent stroke, readmission, 1-year mortality | In the first year post-stroke, the incidence rate per 100 person-months of visiting a community neurologist was 6.41 (95% CI: 5.00–8.20) in the intervention group, com- pared to 3.32 (95% CI: 2.73–4.04) in the control group. In the intervention group, 9.9% suffered from another stroke within 12 months compared to 13% in the control group. Readmissions to a hospital were recorded for 37.4% in the intervention group and 57.4% in the control group. Participants had an incidence rate of 4.03 (95% CI: 2.58–6.28) readmissions per 100 person-months, standard care patients 9.56 (95% CI: 7.99–11.43). Patients with a higher number of comorbidities were admitted to a hospital more frequently. The number of readmissions was accompanied by substantially lower hospital costs in the intervention group (median 6 months costs: €0, IQR: €0–1910) compared to those in the control group (median 6 months costs: €938, IQR: €0–4375). This difference was also confirmed in the linear mixed model. The 12-month survival rate for the intervention group was 0.92 (95% CI: 0.87–0.98) compared to 0.90 (95% CI: 0.87–0.94) in the control group | This study showed the beneficial potential of care coordination for a vulnerable patient population: the utilization rate of important health services was increased, and the rate of hospital readmissions decreased as a result. Future research should focus on the risk of recurrent strokes and the long-term effects of improved care |
| Døhl, 2020 (31) | Multicentre, pragmatic, single-blinded, randomized controlled trial  N=380 (Intervention n=186, control n=194) | A follow-up program comprising monthly individualized coaching by a physiotherapist, physical activity and exercise schedule, training diary | Cost analysis, need variables (primary= MAS, secondary HRQoL) | The average grand total cost were 23,126 Euro in the intervention group, of this in average 1480 Euro were costs related to the intervention. In the control group the average grand total cost were 20,412 Euro. The hospital costs constituted nearly 41% of the total costs in the intervention group and 45% in the control group. The primary care costs constituted 41% of the total costs in the intervention group and 42% in the control group. The higher cost of physiotherapy in the intervention group is due to the cost of the intervention. In intervention group there were a smaller pro- portion of elderly above 80 years, but there were a higher proportion of females and those living alone. There were higher total health care costs in the intervention group than the control group, related to the intervention cost. For the primary care and hospital care there were no cost differences between the intervention and control group. For the GP’s there were indication (p = 0.09) of lower costs in the intervention group. In the specification using clinical outcome measures, MAS and Gait speed were both strongly associated with the use of health care services, whereas there was no such association for Barthel. MMSE was associated with the use of primary care costs. Gait speed, on the other hand was negatively associated with all types of health care services, except hospital care. Implying that increased Gait speed reduced the use of health care services, and thereby costs. | Long term, regular individualized coaching did not reduce health care costs compared to standard care. We found that MAS, Gait speed, HADS and mRS were significant predictors for future health care use. The generic EQ-5D-5 L performed equally well as the more detailed battery of outcome measures, suggesting that HRQoL measures may be a simple and efficient way of identifying patients in need of health care after stroke and targeting groups for interventions |
| Duncan, 2020 (32) | Pragmatic cluster-randomized trial  6024 patients, 41 hospitals (Hospitals n=40, patients intervention n=2689, control n=3193) | Education, secondary prevention, rehabilitation, recovery, referrals to community-based resources, and caregiver support services by telephone follow-up | Primary: Functional status  Secondary: 90-day mortality, depression | Of intervention hospitals, 58% had uninterrupted intervention delivery. Thirty-five percent of patients at intervention hospitals attended a COMPASS clinic visit. The primary outcome was measured for 59% of patients and was not significantly influenced by the intervention. Mean Stroke Impact Scale-16 (±SD) was 80.6±21.1 in TC versus 79.9±21.4 in usual care. Home blood pressure monitoring was self-reported by 72% of intervention patients versus 64% of usual care patients (adjusted odds ratio, 1.43 [95% CI, 1.21–1.70]). No other secondary outcomes differed. | Although designed according to the best available evidence with input from various stakeholders and consistent with Centers for Medicare and Medicaid Services TC policies, the COMPASS model of TC was not consistently incorporated into real-world health care. No significant effect of the intervention on functional status at 90 days post-discharge was found |
| Ellis-Hill, 2019 (33) | Two-centre, 24-month, parallel-arm RCT with qualitative and economic components  N=56 (Control n=25, intervention n=22) | Arts and health practitioner-led group sessions (psychosocial and wellbeing) | The outcomes were self-reported measures of well-being, mood, capability, health-related quality of life, self-esteem and self-concept (baseline and 5 months post randomisation) | Despite a low recruitment rate (14%; 95% CI 11% to 18%), 88% of the recruitment target was met, with 29 participants randomised to HoS and 27 to UC (57% male; mean (SD) age=70 (12.1) years; time since stroke=9 (6.1) months). Follow-up data were available for 47 of 56 (84%; 95% CI 72% to 91%). Completion rates for a study-specific resource use questionnaire were 79% and 68% (National Health Service and societal perspectives). Five people declined HoS post randomisation; of the remaining 24 who attended, 83% attended ≥6 sessions. Preliminary effect sizes for candidate primary outcomes were in the direction of benefit for the HoS arm. Participants found study processes acceptable. The intervention cost an estimated £456 per person and was well-received (no intervention- related serious adverse events were reported). | Findings from this first community- based study of an arts and health intervention for people poststroke suggest a definitive RCT is feasible. |
| Feng et al., 2023 (34) | 6-month follow-up of an open, prospectively comparative study  Control n=94, intervention n=95 | Full integrated care pathway for older stroke patients including nursing and caregiver support | Quality of life | Significant improvement in mental component of SF-36  SF-36 PCS baseline:  Control 31.7 ± 0.6, intervention 32.0 ± 0.8  After 3 months:  Control 39.5 ± 2.8, intervention 40.4 ± 3.1  After 6 months:  Control 41.7 ± 3.0, intervention 42.2 ± 1.6 (between group difference 9.63, p=0.105)  SF-36 MCS baseline:  Control 35.4 ± 3.2, intervention 35.2 ± 2.9  After 3 months:  Control 42.1 ± 3.8, intervention 41.7 ± 6.6  After 6 months:  Control 46.2 ± 2.9, intervention 51.5 ± 0.7 (between group difference 8.44, p<0.001) | Older people with stroke who received IHSC services, maintained higher levels of mental aspect of quality of life and experienced less disease burden during 6-month follow-up, compared with those who received usual integrated care. |
| Feng, 2021 (35) | Randomised controlled trial  N=120 (Control n=60, intervention n=60) | Trained multidisciplinary team providing an intervention plan based on the patient’s condition and treatment-related information, and arranging specific plans for family visits and telephone follow-ups, continued support and follow-up in the community (full pathway) | The changes of self-care ability, compliance behavior, self-efficacy, and adverse mood before and after intervention were compared between the two groups | The modified Barthel Index (MBI) score of the observation group after 3 months of intervention was higher than that of the control group (P<0.05). In the observation group, the changes in the proportion of medication, reasonable diet, moderate exercise, and regular return visits after 3 months were higher than those in the control group (P<0.05). The General Self-efficacy Scale (GSES) score of the observation group was higher than that of the control group after 3 months of intervention (P<0.05). Zung’s Self-rating Anxiety Scale (SAS) and Self-rating Depression Scale (SDS) scores in the observation group were low after 3 months of intervention than those in the control group (P<0.05). | HCISM applied to the home rehabilitation of the disabled elderly with stroke can improve life self-care ability and self-efficacy, improve medical compliance behavior, and reduce negative emotions, and is thus worthy of further promotion |
| Freburger et al., 2022 (COMPASS) (36) | Randomised Multi-location controlled trial  Control n=1193, intervention n=1069 | Telephone follow-up and clinic visit with individualised care plan | Readmission, mortality | Skilled nursing facility or inpatient rehabilitation facility:  Patients with one or more admissions, n (%): Intervention 150 (14.0); Control 142 (11.9)  Skilled nursing facility:  Patients with one or more admission, n (%): Inervention 133 (12.4); control 123 (10.3)  Inpatient rehabilitation facility  Patients with one or more admission, n (%): Intervention 28 (2.6); control 32 (2.7)  Mortality: Intervention 88/1069 (8.2%), control 105/1193 (8.8%) | No difference in readmission rates or mortality between intervention and control groups |
| Fu, 2020 (37) | Prospective, randomised, open trial of two active and one control interventions with blinded endpoint assessment  N=400 (Primary analysis: control n=125, T1 n=123, t2 n=133) | One-to-one qualitative assessment of patient views and needs, education and goal setting | Primary: QoL  Secondary: Mortality, recurrent stroke, rehospitalisation at 12 months | Twelve months following stroke, participants in either of the TC groups (i.e. TC1 TC2) scored 2.9 (95% confidence intervals (CI) 0.95 to 4.9, p 0.004) points higher (better) than control on the Short Form 36 Physical Component Summary. This difference remained significant when adjusted for pre-specified baseline variables. There was a dose effect with Short Form 36 Physical Component Summary scores increasing by 1.9 points (95% CI 0.8 to 3.1, p < 0.001) for each extra Take Charge session received. Exposure to the Take Charge intervention was associated with reduced odds of being dependent (modified Rankin Scale 3 to 5) at 12 months (TC1 + TC2 12% versus control 19.5%, odds ratio 0.55, 95% CI 0.31 to 0.99, p = 0.045). | Confirming the previous randomized controlled trial outcome, Take Charge—a low-cost, person- centered, self-directed rehabilitation intervention after stroke—improved health-related quality of life and independence. Two sessions, six weeks apart, were better than a single session. Improvements were also seen in basic and advanced activities of daily living and independence. |
| Gao, 2017 (38) | A single-blind, randomised, controlled trial that compared three intervention groups, with subgroups stratified by time after stroke  N=273 (Group A n=91, Group B n=91, group C n=92)  Analysis groups: Group A n=86, Group B n=85, Group C n=87 | Citalopram, psychological intervention for depression, cognitive behavioural education | Depression, melancholia, side-effects, activities of daily living, functional impairment | When stratification was not considered, the scores of Group B on the Melancholia Scale were lower than those of Group A (P = 0.02); when the four time-based subgroups were analysed, significant differences were observed between Groups A and B (PMES = 0.02, PHAMD17 = 0.02) in the group recruited six months after discharge and between Groups A and C (PMES = 0.01) in the last time period nine months after discharge | The effects of citalopram or cognitive behavioural therapy is similar to the effect of rehabilitation alone for early-onset post-ischaemic depression; rehabilitation and citalopram for delayed-onset post-ischaemic depression; and rehabilitation and cognitive behavioural therapy for late-onset post- ischaemic depression are more effective than rehabilitation alone |
| Geng, 2019 (39) | Randomised controlled trial  N=101 (Control n=30, intervention n=30) | Physical function, emotional function, lifestyle, medication adherence | Systolic blood pressure, BMI, cholesterol, physical activity, smoking and alcohol consumption at 6 months | The TC intervention improved the discharged elderly stroke patients’ health behaviors, activities of daily living, quality of life, and stroke knowledge. The intervention also controlled the patients’ systolic blood pressure, body mass index, total cholesterol, triglycerides, and low-density lipoprotein cholesterol. | This study provided evidence of concurrent self-reported and objective clinical indicators in discharged elderly stroke patients for the impact of the IBM-based 3-month TC intervention, which can be recommended for clinical practice |
| Graven et al., 2016 (40) | Prospective randomised controlled trial  Control n=46, intervention n=48 | Physiotherapist-led comprehensive multifactorial care pathway individualised to each participant | Depression (Geriatric depression score [GDS-15]) | At 12 months, the mean GDS-15 score was lower in the intervention group (3.6) compared to the control group (4.8). A lower proportion of participants in the intervention group (14.6%) had significant depressive symptoms (GDS-15 ≥6) at 12 months compared to the control group (34.8%). At 12 months. | Positive impact of individualised, goal-centered, comprehensive poststroke management for reducing depressive symptoms during the first year poststroke. |
| Greger, 2021 (41) | Single-centre retrospective chart review  Control n=4516, inv n=1274  Matched groups: Control n=171, inv n=171 | Pharmacist led medication review, counselling, risk factor management (lifestyle) | Primary: change in responsiveness to antiplatelet medication  Secondary: Lifestyle (including adherence) | At the first visit, a total of 168 instances of optimizing antiplatelet therapy or counseling patients on adherence were identified in the PFT group while 35 instances were identified in the non-PFT group. Over the course of 1 year, 24 patients in the PFT group were counseled on adherence while only five patients in the non-PFT group were counseled (P = .0008). Similarly, 47 drug-drug interactions were identified by pharmacists compared with 10 identified by providers (P < .0001). All intervention data collected resulted in statistically significantly more pharmacist interventions than provider interventions, except decreasing the dose of aspirin. PFT results were reviewed for patients in the PFT group (n = 171). Baseline antiplatelet response results revealed 27% (n = 46) of patients were responsive to their current antiplatelet therapy and 73% (n = 125) were nonresponsive (Figure 1). A total of 25% (n = 43) of PFT patients were unable to be tested during their first visit generally due to drug-drug interactions with their antiplatelet therapy, a drug-test interaction, technical difficulties, or medication non- adherence. | Pharmacist interventions optimized secondary stroke/TIA prophylaxis therapy, decreased drug-drug interactions, and increased adherence counseling. Patients who underwent PFT and pharmacist intervention transitioned from non- responsive to responsive to their antiplatelet therapy regimen |
| Gun Young, 2021 (42) | Open label non-randomised study with non-equivalent control group design  N=50 (Control n=23, inv n=20) | Integrated management programme assessing physical function (structured exercise), cognitive function, and depression | Depression (Beck Depression inventory at 4 and 8 weeks) | The interaction between group and time was significant, indicating that the experimental group showed improvement in gait speed, balance ability, cognitive function (linguistic ability, linguistic memory, reasoning), and a decrease in depression compared to the control group | These results indicate that the integrated management program developed herein was beneficial in restoring physical function, cognitive function, and depression in subacute stroke patients. |
| He et al., 2023 (43) | Randomized, historically controlled clinical trial  Control n=124, intervention n=161 | Rapid recovery implementation process to optimise patient recovery post-surgery | Functional status (mRS), QoL (EQ-5D) | mRS score at discharge: 2.52 (1.89) Control, 3.61 (5.74) intervention, p<0.001  mRS score 3 month after discharge: 2.38 (1.77) (control), 2.01 (1.95) (intervention), p = 0.097  EQ-5D-5 L:  Mobility 1.69 (0.78) (control),1.6 (0.82) (intervention), p=0.349  Self-care 1.56 (0.78) (control), 1.65 (0.82) (intervention), p=0.394  Usual activities 1.6 (0.76) (control), 1.61 (0.78) (intervention), p=0.897  Pain/discomfort 1.27 (0.48) (control),1.33 (0.58) (intervention), p=0.383  Anxiety/depression 1.39 (0.56) (control), 1.23 (0.43) (intervention), p=0.013  Visual analog scale 64.72 (25.93) (control), 72.29 (26.94) (intervention), p=0.017 | Statistically significant differences in mRS were seen at discharge but not at 3 months. Significant differences were found in anxiety/depression and overall quality of life, with the intervention group showing better outcomes. |
| Hjelle, 2019 (44) | Multicentre, prospective, randomized controlled trial  Enrolled n=353, randomised n=322 (Control n=156, inv n=166) | Dialogue-based intervention delivered in patient home by nurse/OT (psychological) | Primary: Psychosocial wellbeing.  Secondary: depression, QoL, sense of coherence | After controlling for the baseline values, no significant benefit was found in the intervention group over the control group (odds ratio (OR): 0.898: 95% confidence interval (95% CI): 0.54-1.50, p = 0.680) 6 months post-stroke | Psychosocial well-being improved during the first 6 months after stroke in both arms of the trial, but no statistically significant benefit of the dialogue-based intervention was found compared with usual care. |
| Huang et al., 2023 (ERNI) (45) | Randomised non-blinded controlled trial  Control n=45, intervention n=45 | Early rehabilitation and nursing intervention (ERNI), internet + continuous nursing program | QoL (SS-QoL) | QoL scores  Control before intervention 31.8±4.3, after 42.5±3.7, p<0.005  ERNI group before intervention 31.5±4.0, after 50.1±3.1, p<0.001  Between group difference after intervention, p<0.001 | ERNI was associated with significantly better QoL |
| Jones et al., 2016 (46) | Cluster randomised controlled trial  Control n=30, intervention n=36 | Bridges self-management program (SMP) comprising one-to-one rehabilitation sessions and stroke workbook | Hospital anxiety and depression scores, QoL (SAQOL), adherence to intervention | HADS-A scores:  Baseline: control 7.4±5.1, intervention 7.5±5.3  6 weeks: control 7.3±4.9, intervention 7.5±4.9  12 weeks: control 7.4±4.5, intervention 6.6±5.3  HADS-D scores:  Baseline: control 7.1±3.4, intervention 6.9±4.2  6 weeks: control 8.2±4.1, intervention7.1±4.5  12 weeks: control 8.1±4.1, intervention 7.1±4.3  SAQOL mean scores:  Baseline: control 3.2±0.8, intervention 3.4±0.8  6 weeks: control 3.1±1.0, intervention 3.3±0.8  12 weeks: control 3.1±0.8, intervention 3.4±0.8  Thirty-nine participants (98%) completed baseline measures and 36 participants completed week 12 outcome measures (90%) in intervention sites, compared with 35 (92%) completing baseline outcomes and 30 (79%) completing week 12 outcomes measures in control sites. | No significant differences in QoL or anxiety/depression but responsiveness to intervention was shown |
| Joubert et al., 2020 (ICARUSS) (47) | Double blind randomized controlled trial  Control n=137, intervention n=112 | ICARUSS (Integrated Care for the Reduction of Secondary Stroke) intervention involving personalizing stroke risk factors for the stroke survivor and ongoing support from a stroke specialist and primary care physician vs usual care. | Risk factors (blood pressure, cholesterol, BMI, alcohol) | At the 12-month evaluation, there was a significant decrease in systolic blood pressure (129.4 [14.7]) from baseline (134.6 [16.7]) in the intervention group of 5.2mmHg (p<0.01), control 133.7 (17.0) at baseline to 131.7 (16.3) at 12 months, p=0.29.  Fasting total cholesterol (mmol/l):  Intervention: Baseline 5.00 (1.17), 12 month 4.19 (0.99)  Control: Baseline 4.36 (1.14), 12 month 4.11 (0.90)  BMI:  Intervention: Baseline 27.78 (5.38), 12 month 27.97 (6.09), p=0.50  Control: Baseline 27.45 (4.94), 12 month 28.52 (4.77), p=0.02  Alcohol (no o drinks per week) Intervention: Baseline 8.4 (16.5), 12 month 4.8 (10.1), p=0.04  Control: Baseline 6.0 (10.2), 12 month 4.6 (7.2), p=0.26 | In stroke survivors, the ICARUSS model was superior to usual care with respect to best-practice recommendations for traditional risk factors as well as behavioral and functional outcomes. |
| Jung, 2021 (48) | Non-randomised single arm trial with historical control group  N=313 (Control n=96, int n=227)  Analysis: Control n=96, int n=88 | Exercise based intervention tailored to each patient | The primary outcome measurements used were Berg Balance Scale (BBS), Mini-Mental State Examination (MMSE), Modified Barthel Index (MBI), physical component summary (PCS), and the mental component summary (MCS) of the Short-Form Survey 36 (SF-36), and Beck Depression Inventory (BDI) according to the WHO’s International Classification of Functioning, Disability, and Health (ICF). | Differences in BBS, MBI, and PCS components in SF-36 were more statistically significant in the SAVE group than that in the control group (p < 0.05). Patients in the SAVE group showed more significant improvement in BBS, MBI, and PCS components in SF-36 as compared to that in the control group | This evidence-based SAVE intervention can optimize patient recovery after a subacute stroke while keeping the available resources in mind. |
| Kalav, 2022 (49) | Single centre, single-blinded randomised controlled experimental study  N=210 (Control n=34, int n=34) | Individualised patient education with follow-up | Primary: Self efficacy, QoL, patient satisfaction  Secondary: healthy eating habits, physical activity (lifestyle), BP (risk factors) | No significant differences were found between the two groups with respect to self-efficacy and QoL. Patient satisfaction was significantly higher in the IG. Positive feedback percentages of the patients in the IG regarding stroke knowledge level and consuming whole-grain food at the end of the 12 weeks increased significantly. This protocol had no significant effects on the secondary outcomes. | The CCM-based interventions may increase the satisfaction level of the patients and help to raise awareness of the risk of a secondary stroke. More studies are needed to examine the effects of these interventions |
| Kam Yuet Wong et al., 2022 (50) | Randomised controlled trial  N=2612 (Control n=58, int n=58) | Holistic MDT-based exercise training programme with psychological and social support | The primary outcome was EQ-5D-5L (Chinese). Secondary: SIS, ADL, self-efficacy | Both intervention and control groups showed improvement in EQ-5D-5L from baseline to post-intervention (0.66 versus 0.83, P < 0.001) and (0.66 versus 0.77, P < 0.001), respectively, and there was signiﬁcant group-by-time interaction in EuroQol-Visual Analogue Scale from baseline to post-intervention at 90 days and follow-up at 180 days with the intervention group experiencing better improvement. Similarly, signiﬁcant interaction eﬀects were also found in the Stroke Impact Symptom scale, self-eﬃcacy and modiﬁed Barthel Index | Home-based transitional care was eﬀective in improving QOL, symptoms, self-eﬃcacy and activities of daily living |
| Khramov, 2021 (51) | Prospective randomized controlled parallel-group study  N=122 (Control n=59, int n=63) | Exercise, massage, PT and OT, social adaptation, goal setting | QoL, depression | The functional, cognitive, psychological and emotional states and the level of social activity were monitored for 6 months. Group 2 demonstrated more pronounced improvement than group 1 after 6 months of follow-up, confirmed by higher Rivermead (p = 0.011) and SS-QOL (p < 0.05) scores and lower DASH and Beck scores (p = 0.015 and p < 0.001, respectively). In both groups, MMSE (cognitive function) scores slightly increased, but the differences between the groups were insignificant. | The proposed post-stroke rehabilitation program is effective in helping patients regain their independence and improve social adaptation. The scales and scores used in the study, especially DASH and SS-QOL, are sensitive to changes in the functional state of stroke survivors with moderate impairments |
| Koch, 2020 (52) | Prospective randomised study  N=131 (control n=45, int n=86) | Combined exercise and cognitive training (psychological) | The primary outcomes were feasibility and adherence to the 12-week intervention and safety. The principal secondary outcome was global cognition. Other secondary cognitive outcomes included multiple tests measuring working memory, processing speed, verbal/visual learning and memory, and executive function with the Hopkins Verbal Learning Test Revised, Hopkins Verbal Learning Test Revised Delayed Recall, Grooved Pegboard, Stroop Delis Kaplan Executive Function Test, WAIS-IV Coding Digit Symbol Substitution Test, Brief Visuospatial Memory Test-R, Brief Visuospatial Memory Test-R Delayed Recall Digit Span Backwards, and the CogState Brief Battery | The observed-over-expected visit ratio was significantly higher in the intervention than in the control group (0.74±0.30 versus 0.54±0.38; P=0.003). A total of 99 adverse events were reported by 59 participants, none of which were serious and related to the intervention. Greater gains in physical, cognitive, and mood outcomes were found in the CARET+CTI group than in the control group, but were not statistically significant after adjustments. The most common reason for withdrawal was lack of time/having to return to work, newly arising medical issues, and that the control intervention was not challenging enough | A CARET+CTI intervention, after stroke, is safe, feasible, and has satisfactory participant adherence over 12 weeks |
| Langhorne et al., 2017 (53) | Pragmatic, prospective, parallel-group, multicentre, international, RCT with blinded assessment of outcomes and an intention to treat analysis  N=2104 (Control n=1050, int n=1054) | Very early mobilisation (within 24h of stroke onset) Exercise | The primary outcome was survival without major disability [modified Rankin scale (mRS) score of 0–2] at 3 months after stroke. Secondary efficacy outcomes were an assumption-free ordinal shift across the range of the mRS, time (days) to walk 50 m unassisted and the proportion of patients achieving unassisted walking by 3 months. | Compared with UC, VEM patients mobilised 4.8 hours [95% confidence interval (CI) 4.1 to 5.7 hours; p < 0.0001] earlier, with an additional three (95% CI 3.0 to 3.5; p < 0.0001) mobilisation sessions per day. Fewer patients in the VEM group (n = 480, 46%) had a favourable outcome than in the UC group (n = 525, 50%) (adjusted odds ratio 0.73, 95% CI 0.59 to 0.90; p = 0.004). Results were consistent between Australasian and UK settings. There were no statistically significant differences in secondary outcomes at 3 months and QoL at 12 months. Dose–response analysis found a consistent pattern of an improved odds of efficacy and safety outcomes in association with increased daily frequency of out-of-bed sessions but a reduced odds with an increased amount of mobilisation (minutes per day). | Patients in the VEM group were mobilised earlier and with a higher dose of therapy than those in the UC group, which was already early. This VEM protocol was associated with reduced odds of favourable outcome at 3 months cautioning against very early high-dose mobilisation. At 12 months, health-related QoL was similar regardless of group. Shorter, more frequent mobilisation early after stroke may be associated with a more favourable outcome |
| Lee, 2018) (54) | Open-label parallel group study  N=31 (Control n=17, int n=14) | Horticulture-based exercise therapy | Upper limb function, grip strength, pinch force, fine motor skills, balance, ADL, depression, rehabilitation stress, rehabilitation motivation, fall efficacy. | Stroke patients in the HT group showed significantly improved upper limb function, hand force, balance, fall efficacy, activities of daily living, and decreased depression (P < 0.05). By contrast, no significant change was noted in the control group. In addition, 85.7% of the stroke patients in the HT group reported being very satisfied or satisfied with the HT program | In conclusion, the HT program based on task-oriented training improved the patients’ physical and psychological function after stroke rehabilitation. These study results suggest that implementing an HT program in a rehabilitation hospital will effectively contribute to functional recovery after stroke |
| Lewthwaite, 2018 (55) | Parallel group, three-arm, single blind, superiority randomized controlled trial of a theoretically-defensible, upper extremity rehabilitation program  N=361 (UCC n=122, DEUCC n=120, ASAP n=119) | Accelerated Skill Acquisition Program (ASAP) comprising skill acquisition through task-specific practice, impairment mitigation to increase capacity, and motivational enhancements to build self- confidence (exercise, psychological) | The log-transformed WMFT time score at the 1 year endpoint is the primary outcome measure for the trial and the basis of a priori sample size and sensitivity estimates. The SIS hand domain and full SIS constitute the secondary outcome measures. | Across all groups, most improvement occurred during the treatment phase, followed by change more slowly during follow-up. Compared with DEUCC and UCC, ASAP group gains were greater during treatment for Stroke Impact Scale Hand, Strength, Mobility, Physical Function, and Participation scores, self-efficacy, perceived health, reintegration, patient-centeredness, and quality of life outcomes. ASAP participants reported higher Motor Activity Log–28 Quality of Movement than UCC posttreatment and perceived greater study- related improvements in quality of life. By end of study, all groups reached similar levels with only limited group differences | Customized task-oriented training can be implemented to accelerate gains across a full spectrum of patient- reported outcomes. While group differences for most outcomes disappeared at 1 year, ASAP participants achieved these outcomes on average 8 months earlier |
| Li et al., 2023 (56) | Randomised controlled trial  Control n=80, intervention n=80 | Physiotherapy group rehabilitation (We chat) updates in outpatient setting every 2 weeks where a combination of doctors, nurses and rehabilitation therapists continued follow up | QoL (SS-QoL) | SS-QoL scores:  Baseline: Control 117.59±17.99, intervention 119.94±22.78  1 month: Control 120.10±18.11, intervention 130.83±22.30  3 months: Control 121.89±16.89, intervention 137.46±21.70  Between group p=0.001 (1 month), p<0.001 (3 months) | Significant SS-QOL score differences between intervention group and control |
| Lin, 2022 (57) | Randomised controlled trial  Control n=83, intervention n=83 | Intervention group had a multi-disciplinary clinical nursing pathway (CNP) formed and followed until 11 days post-stroke | Anxiety and depression (45-point scale) | Anxiety scores (A1):  Baseline: Control (G1) 20.98±2.63, intervention 20.68±2.57  After intervention: Control 18.63±2.10, intervention 15.08±2.00 (p<0.05)  Depression scores (A2): Baseline: Control 33.08±3.52, intervention 33.62±4.03  After intervention: Control 29.73±3.92, intervention 22.47±2.64 (p<0.05) | Anxiety and depression were significantly reduced in the intervention group compared with control |
| Lin, 2020 (58) | Randomised controlled trial  N= 152 (control n=114, int n=38)  Analysis: Control n=107, int n=38 | Supervised virtual reality training in addition to early rehabilitation exercise | Outcome data including muscle strength, mood state (depression, anxiety), and functional status were collected at baseline (within 4 hr following admission) and on the day of discharge (7 to 21 days). | Participants in the EG reported increased muscle strength of upper and lower limbs in both affected and unaffected sides, decreased depression and anxiety, and increased functional status at discharge. When the group–time interaction was examined, the EG had greater increased upper limb muscle strength of the unaffected side (ß = 0.34, p < .001) and decreased depression and anxiety scores (ß = −2.31, p = .011; ß = −1.63, p = .047) at discharge compared with the CG. However, there was no difference in the functional status change scores from baseline between EG and CG | A poststroke program that includes both early rehabilitation and VR training has greater benefit in relation to mood state and muscle strength at discharge than early rehabilitation alone. Therefore, an early physical rehabilitation program that includes VR training for acute stroke inpatients should be considered for implementation in clinical settings |
| Liu et al., 2014 (59) | Multi centre randomized controlled trial  Control n=122, intervention n=121 | Early intervention: Standard care but at 48 hours following ICH onset. | Mortality, HrQoL, anxiety | Mortality:  3 months: Control 10/122, intervention 1/121  6 months: Control 12/122, intervention 3/121  SF-36 Mean differences between components at 3 months varied were -2.5 to 5.8 between groups, 6.4-25.5 at 6 months  Zung’s self rating anxiety scale:  3 months: control 51.8 (5.8), intervention 51.8 (4.9), mean difference = 0  6 months: control 55.2 (9.3), intervention 48.9 (4.8), mean difference -6.4 | Commencement of rehabilitation within 48 hours of ICH significantly reduced hospital length of stay and improved long-term survival and morbidity outcomes when compared with standard practices |
| Liu, 2018 (60) | Randomised trial  N=40 (control n=20, int n=20) | Nurse-led personalised education and follow-up | Functional exercise compliance, health status | No significant between-group differences were observed in functional exercise compliance or health status before the intervention was conducted. One month after the intervention, functional exercise compliance and health status were significantly improved in the intervention group, with significant between-group differences in all but one dimension: social functioning. Three months after the intervention, functional exercise compliance and four dimensions of health status – physical functioning (PF), role limitations due to physical health problems (RP), general health perceptions (GH), and mental health (MH) – were significantly improved in the intervention group compared with the control group. Six months after the intervention, only the active advice-seeking of functional exercise compliance and the GH of health status were significantly improved in the intervention group compared with the control group. | A nurse-led transitional care intervention effectively improved the early functional exercise compliance and health status of stroke patients. However, the intervention effect decreased with time after the intervention, and the long-term effect was not ideal |
| Lo et al., 2023 (61) | Randomised controlled trial  Control n=169, intervention n=166 | Virtual multidisciplinary stroke clinic including monthly follow-up calls and educational videos | Primary: self-efficacy  Secondary: depression (GDS) | Depression level (GDS): Baseline (T0): control 8.4 (6.9), intervention 10.2 (6.7)  3 months (T1): control 7.1 (6.7), intervention 8.2 (6.9)  6 months (T2): control 7.8 (7.6), intervention 7.3 (7.5), effect size 0.36 (95% CI: 0.13-0.60) | The intervention reduced depression but not significantly |
| López-Liria et al., 2016 (62) | Prospective cohort study  Control n=67, intervention n=78 | Home-based rehabilitation including physiotherapy versus hospital based rehabilitation (standard care) | QoL (SF-36) | PCS-36 Pre-test: intervention 26.76 (5.07), control 26.15 (5.35), p=0.482  Post-test: intervention 31.95 (8.01), control 32.17 (8.18), p=0.870  MCS-36 Pre-test: Intervention 29.67 (7.48), control 33.41 (13.22), p=0.034  Post-test 35.44: Intervention (10.70), control 37.30 (13.17), p=0.349 | No statistically significant differences between groups |
| Lu et al., 2023 (63) | Randomised controlled trial  Control n=41, intervention n=40 | Internet-based home care model (multidisciplinary) | Readmission, QoL (SS-QoL), anxiety and depression (HADS), adherence to intervention | Rehospitalisation = 16 in control group vs 8 in intervention p=0.043  After 3 months of nursing, there was no significant difference in SS-QOL scores between the intervention group and the control group (P>0.05). After 6 months and 12 months of nursing, the SS-QOL score of the intervention group was statistically higher than that of the control group (P<0.05)  There was no significant difference in HADS scores between the two groups after 3 months of nursing (P>0.05). After 6 months and 12 months of nursing, the HADS score of the intervention group was significantly lower than that of the control group (both P<0.05)  The scores of standardized drug use, rehabilitation training and reasonable diet in the intervention group were higher than those in the control group (all P<0.05) | The intervention was associated with significantly lower readmission rates. Better QoL, lower depression and anxiety rates were observed in the intervention group but only at 6 and 12 months. Adherence to intervention was higher in the intervention group. |
| Luengo-Fernandez et al., 2022 (EXPRESS) (64) | Prospective population-based before (phase 1: April 2002–September 2004; n=310) versus after (phase 2: October 2004–March 2007; n=281) study  EXPRESS: Phase 1 n=310, Phase 2 n=281; Control: Phase 1 n=167, Phase 2 n=224 | EXPRESS Phase 1: Clinic referral (1-2 days), brain imaging and ECG, ultrasound/echo, primary care treatment protocol comprising antiplatelet, statin, BP lowering. Phase 2: As Phase 1 but treatment initiated immediately instead of by primary care, high-dose aspirin or clopidogrel, CT scan during clinic. Follow-up 1, 6, 12, 60, and 120 months after the index event in both Phases. Control: standard care (not referred to study clinic) | Mortality, functional statust (mRS), recurrent stroke, quality of life (EQ-5D) | Death, n (%):  1 month: Control Phase 1 14/167 (8), Phase 1 7/310 (2), Control Phase 2 15/224 (7%), Phase 2 4/281 (1)  6 months: Control Phase 1 16/167 (10), Phase 1 14/310 (5), Control Phase 2 23/224 (10%), Phase 2 9/281 (3)  1 year: Control Phase 1 26/167 (16), Phase 1 24/310 (8), Control Phase 2 32/224 (14), Phase 2 12/281 (4)  5 years: Control Phase 1 77/167 (46), Phase 1 89/310 (29), Phase 2 control 89/224 (40), Phase 2 62/281 (22)  10 years: Control Phase 1 109/167 (65), Phase 1 152/310 (49), control Phase 2 132/224 (59), Phase 2 123/281 (44)  Disability (mRS>2), n (%):  1 month: Control Phase 1 47/167 (33), Phase 1 55/310 (18), control Phase 2 58/224 (32), Phase 2 34/281 (13)  6 months: Control Phase 1 47/167 (34), Phase 1 71/310 (24), control Phase 2 50/224 (29), Phase 2 34/281 (13)*  1 year: Control Phase 1 43/167 (33), Phase 1 60/310 (22), Control Phase 2 47/224 (29), Phase 2 36/281 (14)**  5 years: Control Phase 1 27/167 (34), Phase 1 51/310 (25), Control Phase 2 37/224 (32), Phase 2 50/281 (29)  10 years: Control Phase 1 9/167 (21), Phase 1 44/310 (36). Control Phase 2 22/224 (35), Phase 2 30/281 (27)  Statistically significant at: *p<0.001 & ** p<0.050 when compared to phase 1  Recurrent stroke, n (%)  90 days: Phase 1 32/310 (10%), Phase 2 6/281 (2%)  10 years: Control Phase 1 39/167 (23%), Phase 1 82/310 (26%), Control Phase 2 55/224 (25), Phase 2 55/281 (20%) | Urgent assessment and treatment of patients with transient ischemic attack or minor stroke resulted in a long-term reduction in recurrent strokes and improved outcomes, with little atrophy of the early benefit over time, representing good value for money even with a 10-year time horizon.  A reduction in stroke risk in phase 2 was still evident at 10 years (55/23.3% versus 82/31.6%; hazard ratio=0.68 [95% CI, 0.48–0.95]; P=0.024), as was the impact on risk of disabling or fatal stroke (17/7.7% versus 32/13.1%; hazard ratio=0.54 [0.30–0.97]; P=0.036). |
| MacKay-Lyons et al., 2022 (PREVENT) (65) | Three-site, single-blinded, randomized controlled trial  Control n=90, intervention n=94 | Participants randomized to the PREVENT group engaged in a multi-modal, case-managed program of exercise and education. | CV risk factors including systolic BP, cholesterol, anxiety and depression (HADS) | Resting SBP mmHg:  Baseline: PREVENT 131.8±15.7, Control 131.7±18.4  Post-treatment: PREVENT 125.9±16.7, Control 129.3±18.1  6-months: PREVENT 126.8±15.1, control 128.2±16.8  12 months: PREVENT 130.6±17.5, control 128.9±18.3  Change from baseline between groups -3.5 (-8.8, 1.8)  Resting DBP mmHg  Baseline: PREVENT 76.6±11.1, control 74.9±9.8  Post-treatment: PREVENT 72.2±12.0, control 73.7±14.6  6 months: PREVENT 71.8±8.1, control 73.8±9.0  12 months: PREVENT 73.0±9.7, control 72.5±9.9  Change from baseline between groups -3.2 (-6.3, -.2), p<0.05  LDL-C Mmol/L:  Baseline: PREVENT 2.24±0.8, control 2.24±0.9  Post-treatment: PREVENT 1.94±0.8, control 2.23±0.8  6 months: PREVENT 1.96±0.6, control 2.21±0.8  12 months: PREVENT 2.01±0.5, control 2.10±0.7  Change from baseline between groups -.31 (-.42, -.20), p<0.05  HADS-A 0-21:  Baseline: PREVENT 5.2±4.1, control 5.5±4.0  Post-treatment: PREVENT 5.1±4.1, control 4.4±3.8  6 months: PREVENT 4.6±3.8, control 4.5±3.9  12 months: PREVENT 3.9±3.8, control 4.4±3.6  Change from baseline between groups: -.9 (-2.1, 0,3)  HADS-D 0-21:  Baseline: PREVENT 3.4±2.8, control 3.5±3.2  Post-treatment: PREVENT 3.1±2.9, control 3.4±3.1  6 months: PREVENT 2.9±2.6, control 3.3±2.9  12 months: PREVENT 2.3±2.2, control 3.0±2.6  Change from baseline between groups: -.2 (-1.1, .7) | Significant between-group differences at post-intervention favored PREVENT group over UC: DBPrest (mean difference [MD]: -3.2 mmHg, 95% confidence interval [CI]: -6.3, -.2, P = .04) and LDL-C (MD: -.31 mmol/L, 95% CI: -.42, -.20, P = .02). Trends of improvement in PREVENT group were noted in several variables between baseline and 6-month follow-up but not sustained at 12-month follow-up.  Impact of PREVENT on vascular risk factor reduction was more modest than anticipated, possibly because several outcome variables approximated normative values at baseline and training intensity may have been sub-optimal. |
| Man, 2018 (66) | Nonrandomized comparative group study  CSC n=134 (patients n=159,000), PSC n=1047 (patients n=563,941) | Comparison of stroke care in in-hospital outcomes between primary stroke centers (PSC) and comprehensive stroke centers (CSC) (pathway comparisons) | In-hospital mortality, smoking cessation | Both CSCs and PSCs had good conformity to 7 performance measures and the summary defect-free care measure. Among emergency department admissions, CSCs had higher intravenous tPA (tissue-type plasminogen activator) and endovascular thrombectomy rates than PSCs (14.3% versus 10.3%, 4.1% versus 1.0%, respectively). Door to intravenous tPA time was shorter at CSCs (median, 52 versus 61 minutes; adjusted risk ratio, 0.92; 95% confidence interval, 0.89–0.95). More patients at CSCs had door to intravenous tPA time ≤60 minutes (79.7% versus 65.1%; adjusted odds ratio, 1.48; 95% confidence interval, 1.25–1.75). For transferred patients, CSCs and PSCs had comparable overall performance in defect-free care, except higher endovascular thrombectomy therapy rates. The overall in-hospital mortality was higher at CSCs in both emergency department admissions (4.6% versus 3.8%; adjusted odds ratio, 1.14; 95% confidence interval, 1.01–1.29) and transferred patients (7.7% versus 6.8%; adjusted odds ratio, 1.17; 95% confidence interval, 1.05–1.32). In-hospital outcomes were comparable between CSCs and PSCs in patients who received intravenous tPA or endovascular thrombectomy | CSCs and PSCs achieved similar overall care quality for patients with acute ischemic stroke. CSCs exceeded PSCs in timely acute reperfusion therapy for emergency department admissions, whereas PSCs had lower risk-adjusted in-hospital mortality. This information may be important for acute stroke triage and targeted quality improvement |
| Markle-Reid, 2020 (67) | Prospective one-group pre-test/post-test pragmatic study  N=45 (participated n=30) | Multidisciplinary transitional care intervention delivering training and support to older stroke patients at home with regular consultation and follow-up (pathway) | The primary objective was to determine the feasibility of implementing the TC intervention. The secondary objectives were to (i) explore the preliminary effectiveness of the intervention based on changes in patient-reported health outcomes, and the costs of use of health and social services, from baseline to 6-month follow-up; (ii) determine the feasibility of the study methods; and (iii) determine the most appropriate primary outcome measure for a future RCT. | The intervention was feasible and acceptable to both older adults and providers. From baseline to 6 months, there was no statistically significant difference in health outcomes. However, there was a significant reduction in the total per person use and costs of health services. | This study established the feasibility of conducting a larger randomized controlled trial of this intervention. |
| Minshall, 2020 (68) | Randomised controlled trial  N=458 (Control: stroke patients n=39, carers n=40. 12-month assessment patients n=25, carers n=23  Int: stroke patients n=50, carers n=44. 12-month assessment patients n=27, carers n=18) | Program of personalized psychosocial support – Stroke Care Optimal Health Program (SCOHP) comprising education, self-management and reflective exercises | Primary outcomes were QoL and self-efficacy. Secondary outcomes were anxiety and depression, coping, illness perceptions, work and social adjustment, carer strain and carer satisfaction | Of the 173 participants recruited (89 stroke survivors; 84 carers), a total of 137 participants – 73 stroke survivors (intervention n = 42; usual care n = 31) and 64 carers (intervention n = 35; usual care n = 29) – underwent analysis up to 12 months. No statistically significant differences were found in the primary outcomes between groups over time, though a significant improvement in carer satisfaction was found at 6 months in the intervention group compared to the usual care group. | No significant differences between groups for QoL |
| Mofidi et al., 2018 (69) | Retrospective registry study  Control n=133, intervention 243 | Integrated stroke care pathway for patients with carotid artery stenosis undergoing carotid endarterectomy (CEA). Within 60 minutes: CT scan, stroke team evaluation, thrombolysis is needed; within 24 hours carotid duplex, MR angiogram, diffusion weighted MRI; within 48 hours vascular surgeon review; within 2 weeks carotid endarterectomy, all cases discussed in MDT | Symptom to surgery time, major bleeding, mortality, cost effectiveness, risk of recurrent stroke | Median symptom to surgery time was 11 (0-66) days for the former and 15 (3-90) days for the latter (p < .001). There was no significant difference in peri-operative stroke death rate between integrated (5/243, 2.1%) and standard (2/133, 1.5%) pathways (chi-square = 0.14, p = .73).  Major bleeding: Intervention 6/243 (2.5%), control 3/133 (2.3%)  Recurrent stroke: Intervention 5/243 (2.1%), control 2/133 (1.5%)  CEA through the integrated pathway improved quality adjusted life expectancy by an additional 0.13 (0.64 QALYs [integrated pathway] to 0.51 QALYs [standard pathway]) and was associated with an incremental lifetime cost benefit of £2203.4. | An integrated stroke system of care is cost-effective and associated with significant improvements in quality adjusted life years. |
| Mohammadi, 2022 (70) | Single-blind RCT  N=80 (Control n=36, int n=31) | Personalised care plan providing education, diet, activity, anxiety level, medication with follow-up | QoL (SSQOL) at 3 and 6 months, activities of daily living (ADL) | The results show that the mean scores of the QOL significantly increased after the intervention in the intervention group (before = 130.80; 3 months = 172.19; 6 months = 205.29) compared to the control group (before = 150; 3 months = 144.86; 6 months = 160.66). Also, the mean scores of the ADL significantly increased after the intervention in the intervention group (before = 1.96; 3 months = 3.64; 6 months = 4.87) compared to the control group (p < .05). The effect size is equal to 0.501 and 0.245 for QOL and ADL, respectively. | The findings show that the care program based on a PCM recovered the QOL and ADL of stroke participants more than other interventions. |
| Nakibuuka et al., 2016 (71) | Non-randomised controlled  Control n=125, intervention n=126 | Stroke care bundle including rapid screening, scans, antiplatelet therapy and physiological monitoring | Mortality at 7, 30 days, in-hospital mortality | Mortality within 7 days was higher in the intervention group compared to controls (RR 13.1, 95% CI 3.3–52.9). There was no difference in 30-day mortality between the two groups (RR 1.2, 95% CI 0.5–2.6). There was better 30-day survival in patients with severe stroke in the intervention group compared to controls (P = 0.018). The median survival time was 30 days (IQR 29–30 days) in the control group and 30 days (IQR 7–30 days) in the intervention group. In the intervention group, 41patients (32.3%) died in hospital compared to 23 (18.1%) in controls (P < 0.001). | While implementing elements of a stroke-focused ICP in a Ugandan national referral hospital appeared to have little overall benefit in mortality and functioning, patients with severe stroke may benefit on selected outcomes. |
| Nayeri et al., 2014 (72) | Posttest-only randomised controlled trial study  Control n=30, intervention n=30 | Family centred care program including education for patients and caregivers | Adherence to intervention, readmission, stroke recurrence | Readmission: Control 8/30 (26.6%), intervention 3/30 (10%)  Stroke recurrence: Control 3/30 (10%, intervention 2/30 (6.6%), p<0.64  Adherence to the therapeutic regimen in 3 areas of rehabilitation, dietary and medicine regime [n (%) & Mean +/- SD]:  ARR:  Good (84–66): Ex Group - 29 (96.66%) & 72.73 ± 4.08. Con Group- 0 (0%) & 51.96 ± 5.04  Average: (65–47): Ex group - 1 (3.34%) ; Con Group - 24 (80%)  Weak: (18–11): Ex group - 0 (0%); Con group - 2 (6.27) t = 17.51; p <0.000  ADR:  Good (33–27): Ex group - 28 (93.33%) & 29.63 ± 2.32; Con group - 0 (0%) & 22.43 ± 2.29  Average (26–19): Ex group - 2 (6.27%); Con Group -28 (93.33%)  Weak (18–11): Ex group - 0 (0%); Con group - 2 (6.27)  t = 12.5; p <0.000  AMR:  Good (18–15): Ex group - 30 (100%) & 17.13 ± 0.89; Con group - 24 (80%) & 15.26 ± 1.04  Average (14–11): Ex group - 0 (0%); Con group - 6 (20%)  Weak (10–6): Ex group - 0 (0%); Con group - 0 (0%) t = 7.40; p<0.000 | Face-to-Face individual education has effective enhanced adherence to ATRs among patients and caregivers. This approach taken for the experimental group has improved both physical and mental health status of patients and reduced complicaitons, and it is recommended to be routine to enhance care quality. |
| Nguyen-Huynh, 2018 (73) | Sequential pre-post intervention group study  N=867 (Control n=310, int n=557) | Telestroke program for thrombolysis through a dedicated tele-neurologist to advise on alteplase delivery (pathway) | Primary: DTN time for alteplase  Secondary: Symptomatic ICH | This study included 310 patients treated with alteplase in the pre–EXpediting the PRrocess of Evaluating and Stopping Stroke period and 557 patients treated with alteplase in the EXpediting the PRrocess of Evaluating and Stopping Stroke period. After implementation, alteplase administrations increased to 62/mo from 34/mo at baseline (P<0.001). Median DTN time decreased to 34 minutes after implementation from 53.5 minutes prior (P<0.001), and DTN time of <60 minutes was achieved in 87.1% versus 61.0% (P<0.001) of patients. DTN times <30 minutes were much more common in the Stroke EXpediting the PRrocess of Evaluating and Stopping Stroke period (40.8% versus 4.2% before implementation). There was no significant difference in symptomatic intracranial hemorrhage rates in the 2 periods (3.8% versus 2.2% before implementation; P=0.29). | Introduction of a standardized modified Helsinki protocol across 21 hospitals using telestroke management was associated with increased alteplase administrations, significantly shorter DTN times, and no increase in adverse outcomes |
| Olaiya et al., 2017 (74) | Pragmatic multicentre, cluster- randomised, controlled trial, with blinded assessment of outcomes and intention-to-treat analysis  N=2516 (Control n=266, int n=267) | Individualised management programme comprising a chronic disease management (CDM) plan and education | Framingham risk score (modified for secondary outcomes). FRS includes cholesterol levels, systolic BP, use of antihypertensives, smoking status and uncontrolled diabetes | From January 2010 to November 2013, 156 general practices (280 patients) were randomly assigned to usual care (control) and 159 (283 patients) to the intervention. The median age was 70.1 years; 65% were male. Overall, >80% of participants were prescribed recommended secondary prevention therapies at baseline. The primary eﬃcacy analysis comprised 533 participants, with 30 either dying or lost to follow-up. In adjusted analyses, no signiﬁcant between-group diﬀerence was found in the cardiovascular risk score at 12 months (0.04, 95% conﬁdence interval -1.7, 1.8). | The eﬀectiveness of an organized secondary prevention programme for stroke may be limited in patients from high-performing hospitals with regular post-discharge follow-up and communication with general practices. |
| Pedapati et al., 2021 (75) | Prospective single-centre, cluster-randomised, open-label, blinded endpoint superiority trial  N=164 (Int n=82, control n=82) | Education and exercise plan tailored to the home needs of patients | Primary: In-hospital mortality  Disability, 3-month mortality (secondary) | Among 164 patients recruited, 82 received intervention, and standard care each. The mean (Standard deviation) Glasgow coma scale of patients was 11.01 (3.4), and National Institute of Health Stroke Scale was 19.17 (8.54). The incidence of complications (72 in the intervention versus 81 in the control group; p=0.56) was not different. Ten patients (12.2%) in the intervention group and 16 (19.5%) in the control group (p=0.20) died in-hospital. Twenty patients (27.8%) in the intervention and twelve (18.2%) in the control group attained modiﬁed Rankin Scale 0-2 at three months (p=0.12). The mortality at three months (20 [24.4%] in the intervention versus 25 [30.5%] in the control group) was not different (p=0.38). The intervention group had fewer complications (42 versus 68 in the control group; p=0.01) during the initial ten days of hospital stay, but adjusted analysis revealed no difference. | A structured educational intervention did not reduce the incidence of hospital-acquired complications, mortality, or morbidity. However, there was a trend towards fewer complications in the initial days of hospital stay. Extended hospital stay, caregiver fatigue, and dilution of the intervention over time might be reasons for the apparent lack of effect |
| Peng, 2017 (76) | Propensity score-matched case-control study using the National Health Insurance data  Original cohort: control n=3159, int n=1480 Matched Cohort: control n=657, int n=657 | PAC program measuring activities of daily living, nutrition, exercise, mobility and language | 90-day mortality, readmission | After propensity score matching, baseline characteristics, stroke severity, and status of healthcare utilization before index stroke admission were similar between cases and controls. After PAC services, the case group obtained signiﬁcant improvement in all functional domains and may have reduced subsequent disability. Among all functional assessments, balance was the most signiﬁcantly improved domain and was suggestive for the reduction of subsequent falls risk and related injuries. Compared with controls, patients receiving PAC services had signiﬁcantly lower 90-day hospital re-admissions [11.1% vs 21.0%, adjusted odds ratio (aOR) 0.47 with 95% conﬁdence interval (CI) 0.34-0.64], stroke-related re- admissions (2.1% vs 8.8%, aOR 0.22, 95% CI 0.12-0.41), and emergency department visits (13.5% vs 24.0%, aOR 0.49, 95% CI 0.37-0.65), but the 90-day mortality rate remained similar between groups (1.4% case group vs 2.0% control group, aOR 0.68, 95% CI 0.29-1.62 | PAC signiﬁcantly improved the recovery of stroke patients in all functional domains through the program, with universal interorganizational staff training, periodic functional assessment, and high- intensity rehabilitation. Further longitudinal research is needed to evaluate the long-term survival beneﬁts and healthcare utilization |
| Rafsten, 2019 (77) | Randomised controlled trial with blinded assessors  N=140 (Control n=71, int n=69) | Very early supported discharge comprising person-centred goal setting for achievement of identified aims (pathway) | The primary outcome as stated in the protocol was anxiety. The secondary outcome was the patients’ degree of overall disability | No significant differences were found between the groups regarding anxiety at three or 12 months post-stroke (p = 0.811). The overall disability was significantly lower in the VESD group 3 months post-stroke (p= 0.004), compared to the control group. However, there was no significant difference between the groups 1 year post-stroke | The VESD does not affects the level of anxiety compared to ordinary rehabilitation. The VESD leads to a faster improvement of overall disability compared to ordinary rehabilitation. |
| Rasmussen et al., 2016 (78) | Interventional, randomised, safety/efficacy open-label trial  Control n=30, intervention n=31 | Multidisciplinary home-based pathway including exercise and goal setting | Primary: mRS at 90 days  Secondary: DALYs, Motor Function, Cognitive Function, QOL, BMI, treatment-associated economy | mRS:  Modified Rankin Scale: Intervention: 2 (2–3); Control: 3 (2–4)  Modified Rankin Scale improvement: Intervention: 1 (1–2); Control: 1 (0–1)  Body Mass Index (BMI): Intervention 23 (21–25); Control: 24 (22–25)  Body Mass Index (BMI) improvement: Intervention: 0 (-0.5–0) Control: −1 (-2–0)  EuroQol-5D™ score: Intervention: 0.77 (0.66–0.79), Control:0.66 (0.56–0.72)  EuroQol-5D™ score improvement: Intervention: 0.19 (0.05–0.51); Control: 0.27 (0–0.49) | Early home-based rehabilitation reduced disability and increased quality of life. Compared to standard care, home-based stroke rehabilitation was more cost-effective. |
| Reeves, 2019 (79) | Open-label (unblinded) 3-group parallel-design clinical trial  N=320 (Group 1 (UC): Global-10 n=77, PAM n=76, Group 2 (SWCM) Global-10 n=82, PAM n=79, Group 3 (SWCM + MISTT website) Global-10 n=81, PAM n=78)  UC, usual care; MISTT, Michigan stroke transitions trial; PAM, pain activation measure; SWCM, social worker–led case management | Home-based social worker–led case management (SWCM) program with or without a website, comprising biopsychosocial assessment followed by development of a personalised plan to address unmet needs (pathway, psychological) | Primary: Anxiety  Secondary: Disability | There were statistically significant changes in Patient-Reported Outcomes Measurement Information System Physical Health (P=0.003) and Patient Activation Measure (P=0.042), but not Patient-Reported Outcomes Measurement Information System Mental Health (P=0.56). The mean change in Patient-Reported Outcomes Measurement Information System Physical Health scores for group-3 (SWCM+MISTT Website) was significantly higher than both group-2 (SWCM; difference, +2.4; 95% CI, 0.46–4.34; P=0.02) and group-1 (usual care; difference, +3.4; 95% CI, 1.41–5.33; P<0.001). The mean change in Patient Activation Measure scores for group-3 was significantly higher than group-2 (+6.7; 95% CI, 1.26–12.08; P=0.02) and marginally higher than group-1 (+5.0; 95% CI, −0.47 to 10.52; P=0.07). | An intervention that combined SWCM with access to online stroke-related information produced greater gains in patient-reported physical health and activation compared with usual care or case management alone. There was no intervention effect on mental health |
| Rodgers, 2019 (80) | Parallel-group observer-blind multicenter individually randomized controlled trial  N=573 (Control n=231, int n=219) | Early supported discharge reviews (EXTRAS) including mobility, self-care, mood (psychological), cognitive function (pathway) | The primary outcome was performance in extended activities of daily living. Secondary outcomes were health status, mood and experience of services. | Mean 24 month Nottingham EADL Scale scores were EXTRAS (n=219) 40.0 (SD 18.1) and usual care (n=231) 37.2 (SD 18.5) giving an adjusted mean difference of 1.8 (95% CI, –0.7 to 4.2). 1155/1338 (86%) of expected EXTRAS reviews were undertaken. Over 24 months, the mean cost of resource utilization was lower in the intervention group: –£311 (–$450 [95% CI, −£3292 to £2787; −$4764 to $4033]). EXTRAS provided more Quality Adjusted Life Years (0.07 [95% CI, 0.01 to 0.12]). At current conventional thresholds of willingness to pay (£20 000 [$28 940] per Quality Adjusted Life Years), there was a 90% chance that EXTRAS could be considered cost-effective. | EXTRAS did not significantly improve stroke survivors’ performance in extended activities of daily living. However, given the impact on costs and Quality Adjusted Life Years, EXTRAS may be an affordable addition to improve stroke care. |
| Saal et al., 2015 (81) | Randomised controlled clinical trial  Control n=111, intervention n=119 | Post-discharge support including follow-up call and visit, education and training sessions | HRQoL BREF, depression (GDS), mortality, recurrent stroke | WHOQOL-BREF  Physical: Intervention: Baseline - 68.5 (17.4); 12 months - 65.8 (18.5); Control: Baseline - 64.4 (20.0); 12 months 63.0 (21.8) Mean difference (95% CI) -0.7 (-4.7; 3.3)  Psychological: Intervention: Baseline - 73.4 (14.9); 12 months 72.2 (17.8); Control: Baseline - 73.1 (16.8); 12 months -72.3 (18.9) Mean difference (95% CI) -1.5 (-5.4; 2.3)  Social: Intervention: Baseline - 73.7 (15.0); 12 month - 73.3 (19.0); Control: Baseline - 75.2 (15.0); 12 months 75.8 (17.3) Mean difference (95% CI) -1.8 (-6.7; 3.1)  Environment: Intervention: Baseline - 77.6 (12.0); 12 month - 78.9 (14.7); Control: Baseline- 81.3 (12.4); 12 month - 81.1 (14.5) Mean difference (95% CI) -0.7 (-4.4; 3.2)  Global: Intervention: Baseline - 61.8 (17.5);l 12 month - 59.6 (20.9); Control: Baseline - 61.7 (19.6); 12 month - 60.9 (25.3) Mean difference (95% CI) -1.8 (-7.1; 3.6)  GDS: Intervention: Baseline - 2.7 (2.2); 12 month - 3.2 (3.2), Control: baseline - 3.6 (3.3); 12 month - 3.6 (3.9) Mean difference (95% CI) 0.4 (-0.4; 1.1)  Reinfarct Intervention: 3 (2.7); Control: 4 (3.8); RR (95% CI) 0.71 (0.16; 3.10); RD (95% CI) -0.01 (-0.09; 0.07)  Mortality Intervention: 5 (3.9); Control: 15 (11.6); RR (95% CI): 0.34 (0.13; 0.90); RD (95% CI) - 0.06 (-0.13; 0.00) | No statistically significant differences were found between groups on HRQOL. No significant differences were observed between the intervention and control groups in depression and somatisation. No significant differences between groups in the number of recurrent strokes. The intervention group had a significantly lower mortality rate (3.9%) compared to the control group (11.6%) with a relative risk of 0.34 (95% CI, 0.13 to 0.90) and an adjusted hazard ratio of 0.32 (95% CI, 0.12 to 0.88). |
| Schwartzbach et al., 2023 (SANO) (82) | Prospective, open-label, cluster-randomised controlled trial  Control n=1283, intervention n=1203 | 1-year patient-centred integrated care intervention including regular follow-up, lifestyle, goal setting and motivational interviewing (SANO) | Recurrent stroke, MI, mortality, risk factors, major bleeding | First recurrent stroke: Control: 50 (3·9%); Intervention: 51 (4·2%); OR 1·00 (95% CI 0·58–1·73); aOR 1·03 (95% CI0·53–1·99)  Myocardial infarction: Control 7 (0·5%); Intervention: 4 (0·3%); OR: 0·61 (95% CI 0·18–2·08)  All-cause death: Control: 31 (2·4%); Intervention: 12 (1·0%); OR 0·42 (95% CI 0·20–0·86) ; aOR 0·61 (95% CI 0·26–1·46)  Readmission to hospital within 1 year of index stroke: Control: 383/1216 (31·5%); Intervention: 375/1169 (32·1%): OR 1·01 (95% CI 0·74–1·38); aOR: 0·99 (95% CI 0·73–1·37)  Blood pressure (including self-reported by interview) <140/90 mm Hg: Control: 657/936 (70·2%); Intervention: 809/1103 (73·3%); OR 1·18 (95% CI 0·79–1·76), aOR 1·08 (95% CI 0·73–1·59)  Blood pressure (physical examination only) <140/90 mm Hg† control: 468/679 (68·9%); intervention: 709/944 (75·1%); OR 1·34 (95% CI 0·86–2·09); aOR 1·34 (95% CI 0·88–2·04)  Diabetes control‡ HbA1c ≤7% (53 mmol/mol) Control: 123/218 (56·4%); Intervention: 144/218 (66·1%); OR: 1·10 (95% CI 1·02–2·21); aOR: 1·45 (95% CI 0·94–2·26)  Adherence to antithrombotic therapy targets§ Control: 1003 (88·3); Intervention: 1029 (89·4); OR 1·10 (95% CI 0·78–1·54); aOR 1·02 (95% CI 0·72–1·47)  Hyperlipidaemia control¶ Achievement of LDL cholesterol targetsControl: 444/729 (60·9%); Intervention: 526/782 (67·3%), OR 1·36 (95% CI 1·00–1·85); aOR: 1·65 (95% CI 1·22–2·22)  Smoking - Quit smoking - Control: 51/198 (25·8%); Intervention: 146/295 (49·5%); OR 3·13 (95% CI 1·72–5·70); aOR: 2·82 (95% CI 1·58–5·04)  Bleeding: Control 8/1152 (0·7%), intervention 31/1151 (2·6%)  Diagnosis of depression 13 (1·1%) 51 (4·3%) | This study demonstrated that the SANO programme had positive effects on the control of some cardiovascular risk factors in patients with stroke, but this did not translate into a reduction in the rate of major cardiovascular events 1 year after the first ischaemic stroke. Since a 1-year follow-up period might be too short to demonstrate a positive effect on recurrent cardiovascular events, longer-term effects need to be considered. |
| Sharma et al., 2019 (COMPASS) (83) | COMPASS was a randomized, double-blind, double-dummy trial  Prior stroke: rivaroxaban + aspirin n=351, rivaroxaban alone n=346, aspirin alone n=335. Total n=1032 | Participants had stable coronary artery or peripheral artery disease and were randomly assigned to receive aspirin 100 mg once daily (n=9126), rivaroxaban 5 mg twice daily (n=9117), or rivaroxaban 2.5 mg twice daily plus aspirin (n=9152). | Primary: composite of CV death, MI and stroke | Recurrent stroke: R+A (n=351) 9/351, R (n=346) 18/346, A (n=335) 20/335  R+A vs A HR 0.42 (0.19-0.92) p=0.03  R vs A: HR 0.88 (0.47-1.67) p=0.70  Major bleeding  R+A 12/351, R 26/346, A 19/335  R+A vs A HR 3.79 (1.07-13.4), p=0.04  R vs A: HR 3.84 (1.08-13.6), p=0.04  CV death, stroke or MI  R+A 23/351, R 42/46, A 38/355  R+A vs A HR 0.57 (0.34-0.96), p=0.04  R vs A: HR 1.07 (0.69-1.66), p=0.77  mRS at 7 days or discharge  R+A (n=83) 2.4±2.0, R (n=115) 2.6±2.1, A (n=141) 2.2±1.  R+A vs A mean difference (95% CI) 0.2 (–0.4 to 0.7), p=0.66  R vs A mean difference (95% CI) 0.3 (–0.1 to 0.8), p=0.29 | In summary, low-dose rivaroxaban plus aspirin is an important new option for efficacious antithrombotic therapy for primary and especially secondary prevention of stroke in patients with atherosclerosis. The absolute risk reduction for secondary prevention is substantial and makes a compelling case favoring the use of 2.5 mg rivaroxaban twice daily plus aspirin in these patients. |
| Shaw, 2020 (EXTRAS) (84) | Parallel-group observer-blind multicenter individually randomized controlled trial  N=573 (Control n=231, int n=219) | Early supported discharge reviews (EXTRAS) including mobility, self-care, mood (psychological), cognitive function (pathway) | The primary outcome was performance in extended activities of daily living. Secondary outcomes were health status, mood and experience of services. | Mean 24-month Nottingham Extended Activities of Daily Living Scale scores were 40.0 (standard deviation 18.1) for EXTRAS (n = 219) and 37.2 (standard deviation 18.5) for usual care (n = 231), giving an adjusted mean difference of 1.8 (95% confidence interval –0.7 to 4.2). The mean intervention group Hospital Anxiety and Depression Scale scores were not significantly different at 12 and 24 months. The intervention did not improve patient health status or carer strain. EXTRAS patients and carers reported greater satisfaction with some aspects of care. The mean cost of resource utilisation was lower in the intervention group: –£311 (95% confidence interval –£3292 to £2787), with a 68% chance of EXTRAS being cost-saving. EXTRAS was associated with 0.07 (95% confidence interval 0.01 to 0.12) additional quality-adjusted life-years. At current conventional thresholds of willingness to pay for a quality-adjusted life-year, there is a 90% chance that EXTRAS is cost-effective | EXTRAS did not improve stroke survivors’ performance in extended activities of daily living. The lack of effect on the primary outcome is likely to be a result of the concept of the intervention rather than the fidelity of the intervention. However, EXTRAS provided more quality-adjusted life-years and was associated with lower costs. In addition, patients and carers in the intervention group were more satisfied with aspects of their overall care. EXTRAS did not improve patients’ health status or carers’ quality of life or stress. A post hoc finding was that the intervention group had fewer cases of anxiety and depression than those who received usual care.  Given the impact on costs and quality-adjusted life-years, there is a high chance that EXTRAS could be considered cost-effective at conventional thresholds of the NHS/UK society’s willingness to pay for a quality-adjusted life-year (currently £20,000 per quality-adjusted life-year), despite there being no difference in the primary outcome. |
| Swanson, 2019 (85) | Retrospective cohort study  Control: n-=2605, rehab only n=689, home nursing only n=535, home nursing and rehab n=898 | Standalone home nursing or rehabilitation, or a combination of both, with or without GP follow-up visits (pathway) | 90-day and 1-year mortality, readmission | There were no significant differences in readmission rates for early GP follow-up. Patients receiving home nursing and/or rehabilitation had higher unadjusted 90- and 365-day readmission rates than those without services (HR from 1.87 to 2.63 depending on analysis, p < 0.001), but the 90-day differences disappeared after risk adjustment, except for patients receiving only rehabilitation. There were no significant differences in mortality rates according to GP follow-up after risk adjustment. Patients receiving rehabilitation had higher mortality than those without services, even after adjustment (HR from 2.20 to 2.69, p < 0.001), whereas the mortality of patients receiving only home nursing did not differ from those without services. | Results indicate that the observed differences in unadjusted readmission and mortality rates according to GP follow-up and home nursing were largely due to differences in health status at admission,  likely unrelated to the stroke. On the other hand, mortality for patients receiving ambulatory rehabilitation was twice as high compared to those without, even after adjustment and irrespective of also receiving home nursing. Hence, assessing the needs of these patients during discharge planning and providing careful follow-up after discharge seems important. |
| Teuschl et al., 2017 (86) | Prospective, Randomized, Open-Label, Blinded Endpoint multicenter, two-arm parallel group clinical trial  N=202 (Control n=87, int n=80) | BP measurement, lifestyle advice, exercise, cognitive training on a regular basis | Primary: cognitive decline  Secondary: depression, lifestyle (inc smoking cessation) | During the first 12 months, adherence to healthy lifestyle and adequately controlled physiological parameters (measured by summary scores) improved significantly in the intervention group compared to controls (p < 0.01). The consumption of reduced-fat milk (p = 0.031), reduced-fat spreads (p = 0.007), and fish (p = 0.021) increased in the intervention group from baseline to 12 months but not in controls. After 24 months, the group difference was significant for the lifestyle summary score but no longer for the combined laboratory lifestyle score | These results demonstrate that intensified individualized multidomain lifestyle interventions in stroke patients are effective in promoting healthy lifestyle in stroke care |
| Towfighi, 2021 (87) | Randomised controlled trial  N=487 (Control n=246, int n=241) | Advanced practice physician (APC) and community health worker (CHW) visits, culturally and linguistically tailored educational materials; vascular risk factor goal tools; and Chronic Disease Self-Management Program (CDSMP) workshops, medication adherence, self-management (pathway) | Primary: Change in SBP  Secondary: Lifestyle (smoking, diet, physical activity) | Mean (SD) systolic BP improved from 143 (17) mm Hg at baseline to 133 (20) mm Hg at 12 months in the intervention group and from 146 (19) mm Hg at baseline to 137 (22) mm Hg at 12 months in the usual care group, with no significant differences in the change between groups. Compared with the control group, participants in the intervention group had greater improvements in self-reported salt intake (difference, 15.4 [95% CI, 4.4 to 26.0]; P = .004) and serum CRP level (difference in log CRP, −0.4 [95% CI, −0.7 to −0.1] mg/dL; P = .003); there were no differences in other secondary outcomes. Although 216 participants (89.6%) in the intervention group received some of the 3 core components, only 35 participants (14.5%) received the intended full dose. | This randomized clinical trial of a complex multilevel, multimodal intervention did not find vascular risk factor improvements beyond that of usual care; however, further studies may consider testing the SUCCEED intervention with modifications to enhance implementation and participant engagement |
| Tung et al., 2021 (88) | Retrospective cohort study  N=261 (Inpatient PAC: n=138, Home-based PAC n=59) | Post-acute care (PAC) delivering physical, OT and speech therapy in inpatient and home settings (pathway) | QoL: EuroQoL EQ-5D (ED5Q) Cost-effectiveness was calculated as the total cost divided by the improvement in Barthel index (BI), Lawton–Brody instrumental activities of daily life scale (IADL), ED5Q and mini nutritional assessment (MNA) scores | The total rehabilitative cost was cheaper in the home-based PAC group (p < 0.001), and the cost-effectiveness is USD 152.474 ± USD 164.661 in the inpatient group, and USD 48.184 ± USD 35.018 in the home group (p < 0.001). Lesser rehabilitative hours per 1-point increase of BI score was noted in the home-PAC group with similar improvements in daily activities, life quality and nutrition in both groups. | Home-based PAC is more cost-effective than inpatient PAC for stroke rehabilitation. |
| Vluggen, 2021 (89) | Two-arm multicenter randomised controlled trial  N=190 (Control n=91, int n=99) | Inpatient neurorehabilitation treatment, home-based self-management training (patient and caregiver), education | Primary: Daily activity  Secondary: QoL (SSQOL) at 6 months | Significant favourable effects for the programme were observed for the subscale autonomy outdoors of the IPA (− 2.15, P = .047, and for the informal caregivers perceived care burden (1.23, P = .048. For the primary outcome daily activity and the other secondary outcomes, no significant effects were observed. | The integrated multidisciplinary programme had no effect on daily activity of older stroke patients. However, patients participating in the programme had a higher level of perceived autonomy of outdoor activities and their informal caregivers perceived a lower care burden. The programme might be promising in providing adequate (after) care, although adaptation of the programme is recommended to increase its feasibility and improve its effects |
| Wang et al., 2021 (90) | Randomised controlled trial  N=184 (Control n=82, int n=84) | Pharmacist-delivered care program providing inpatient and outpatient care - education focused on medication therapy management (MTM), disease state management, and lifestyle for secondary prevention of ischemic stroke | Primary: modifiable risk factors, medication adherence rates  Secondary: Readmission | Compared to CG, at the 6-month follow-up, medication adherence rates significantly increased regarding antihypertensive drugs (92.86% versus 78.57%, P = 0.031), anti-diabetic drugs (91.67% versus 69.7%, P = 0.02), and lipid-lowering drugs (77.38% versus 60.98%, P = 0.022) in IG. Compared to CG, more patients in IG attained the goal surrogate risk factor control markers of hemoglobin A1c (87.88% vs. 52.78%, P = 0.038) and low-density lipoprotein-C (66.67% vs. 48.78%, P = 0.02). Significantly fewer patients were re-admitted to the hospital in IG than CG (7.14% vs. 18.3%, P = 0.03). | Pharmaceutical care programs can improve risk factor control for the secondary prevention of stroke recurrence in ischemic stroke patients. |
| Long Weijan and Zhang, 2017 (91) | Randomised controlled trial  N=96 (Control n=48, int n=48) | Training program for HCPs on comprehensive care for patients, rehabilitation training for caregivers (pathway) | QoL (SS-QOL) at 3 months posy-discharge | There was no significant difference in SS-QOL and Barthel scores between the two groups before discharge. 3 months after the intervention, the total score of SS-QOL in the intervention group (155.02 points ± 16.85 points) was higher than that before the intervention (123.46 points ± 13.23 points) and the control group after the intervention (125.71 points) ±20. Compared with the control group after the intervention, except for the three dimensions of thinking, vision, and work productivity, the scores of the other dimensions were significantly different (P<0.01); the intervention group The patient's Barthel score (62.71±6.76) was higher than that before the intervention (48.65±5.03) and the control group after the intervention (52.60±4.61) | The study of caregivers Comprehensive rehabilitation nursing training combined with continuous nursing after discharge can improve the quality of life of ischemic stroke patients at home. |
| Willeit et al., 2020 (STROKE-CARD) (92) | Pragmatic open-label two-centre randomised controlled trial with blinded outcome assessment of STROKE-CARD disease management programme and standard care  Control n=711, intervention n=1438 | STROKE-CARD care is a disease management programme by a multidisciplinary stroke team that comprises a standardised 3-month visit and access to a web-based patient portal targeting risk factor management, post-stroke complications, comorbidities and cardiovascular warning signs, rehabilitation demands, and patient education, counselling, and self-empowerment. | Stroke, MI, vascular death, QoL (EQ-5D-3L), functional status, major bleeding | Major cardiovascular disease events occurred in 78 patients in the STROKE-CARD care group (5.4%) and in 59 patients in the standard care group (8.3%) (hazard ratio, 0.63; 95% confidence interval: 0.45-0.88; P=0.007).  Stroke or TIA: Intervention 91/1438 (6.3%), control 46/711 (6.5%), HR 0.96 (95% CI 0.67, 1.37), P=0.822  All-cause mortality: Intervention 52/1438 (3.6%), control 35/711 (4.9%), HR 0.72 (95% CI 0.47, 1.10), P=0.131  The median EQ-5D-3L health utility score was 0.783 (IQR 0.687-1.000) in the STROKE-CARD care group and 0.779 (IQR 0.573-1.000) in the standard care group (P < 0.001). A score of 1.0 indicating absence of problems in all five EQ-5D-3L dimensions was achieved by 43% in the STROKE-CARD care group and 32% in the standard care group (relative risk 1.36; 95% CI: 1.20-1.54; P < 0.001).  Major bleeding: Intervention 20/1438 (1.4%), control 15/711 (2.1), HR 0.65 (0.33, 1.27), P=0.210  mRS ≤2: Intervention 1150/1413 (81.4%), control 536/704 (76.1%), HR 1.07 (1.02, 1.12), P=0.006 | The pragmatic and easily implementable STROKE-CARD care programme reduced cardiovascular risk and improved health-related quality of life and functional outcome in patients with acute ischaemic stroke or TIA |
| Wong and Yeung 2014 (93) | Randomised controlled trial  Control n=54, intervention n=54 | The intervention group received the transitional care program (TCP) which was commenced before discharge and lasted for 4 weeks after discharge. The TCP had 3 components 1) holistic care delivered by holistic care managers (HCMS), 2) transitional care track and 3) holistic care managers. | QoL (SF-36), depressive symptoms (CES-D), readmission | SF-36 PCS:  After 4 weeks: Control 47.1 (1.2), intervention 47.9 (1.2)  After 8 weeks: Control 47.3 (1.4), intervention 48.6 (1.4)  F(P-value) after 4 weeks 13.76 (<0.001), after 8 weeks 37.60 (<0.001), interaction effect p=0.006  SF-36 MCS: After 4 weeks: Control 49.2 (1.1), intervention 49.9 (1.0)  After 8 weeks: Control 49.4 (1.4), intervention 49.9 (1.0)  F(P-value) after 4 weeks 13.72 (<0.001), after 8 weeks 7.67 (0.007), interaction effect p=0.591  CES-D-HK (median [10^th^-90^th^ percentile]):  Total after 4 weeks: Control 4 (1.5–8), intervention 2 (0–5), p<0.001  Total after 8 weeks: Control 4 (0–7), intervention 2 (0–4), p<0.001  Readmission rates at 8 weeks: Control 14.8%, intervention 7.4% , p=0.358 | The TCP improved QoL and reduced depression. Readmission rates in the intervention group were lower but this did not reach statistical significance |
| Wu, 2020 (94) | Randomised controlled trial  N=64 (Control n=31, int n=30) | Home remote rehabilitation based on a collaborative care model creating a personalised plan delivered through videoconferencing (pathway) | Primary: Motor function, balance ability  Secondary: quality of life | Both groups were signiﬁcantly improved in terms of motor function and quality of life, but the intervention group showed greater improvement in Fugl-Meyer Motor Function Assessment (intervention group = 83.70 ± 4.44, control group = 75.29 ± 2.89), Berg Balance Scale (intervention group = 43.13 ± 2.32, control group = 38.29 ± 2.70) and Stroke-Speciﬁc Quality of Life Scale (intervention group = 190.57 ± 5.09, control group = 175.90 ± 5.78). Group-time interaction was signiﬁcant in motor function and quality of life. | Collaborative care model based telerehabilitation exercise train- ing program can safely and effectively improve the recovery of motor function and improve the quality of life in patients with stroke. |
| Xi, 2017 (95) | Randomised controlled trial  N=90 (Control n=45, int n=45) | Home remote rehabilitation based on a collaborative care model creating a personalised plan delivered through videoconferencing (pathway) | Out-of-hospital rehabilitation compliance, exercise rehabilitation effect and quality of life | Six months after discharge, the total score of rehabilitation training compliance (11.60 ± 1.67), Fugl-Meyer score (76.27 ± 10.23), Barthel index (72) in intervention group .08 ± 12.56 points) and overall health score (72.68 ± 12.84 points) than the control group (9.58 ± 1.74 points, 68.64 ± 9.88 points, 60 points .44 points ± 11.42 points, 64.13 points ± 13.26 points), the difference was statistically significant (P <0.05). | The out-of-hospital continuous nursing intervention improves the compliance of stroke patients with out-of-hospital rehabilitation, promotes the recovery of the patient's limb motor function and the improvement of daily living ability, and also improves the patient's quality of life |
| Yan et al., 2021 (96) | Open-label, two-arm cluster-randomized controlled trial  villages n=50, participants n=1299 (Control n=615, int n=611) | Primary care based app (SINEMA) to improve communication between HCPs and patients (mhealth) | The primary outcome was the 12- month change in systolic blood pressure (BP). Secondary outcomes were predefined, including diastolic BP, health-related quality of life, physical activity level, self-reported medi- cation adherence (antiplatelet, statin, and antihypertensive), and performance in “timed up and go” test. | The program was implemented with high fidelity, and the annual program delivery cost per capita was US$24.3. There was a significant reduction in systolic BP in the intervention as compared with the control group with an adjusted mean difference: −2.8 mm Hg (95% CI −4.8, −0.9; p = 0.005). The intervention was significantly associated with improvements in 6 out of 7 secondary outcomes in diastolic BP reduction (p < 0.001), health-related quality of life (p = 0.008), physical activity level (p < 0.001), adherence in statin (p = 0.003) and antihypertensive medicines (p = 0.039), and performance in “timed up and go” test (p = 0.022). We observed reductions in all exploratory outcomes, including stroke recurrence (4.4% ver- sus 9.3%; risk ratio [RR] = 0.46, 95% CI 0.32, 0.66; risk difference [RD] = 4.9 percentage points [pp]), hospitalization (4.4% versus 9.3%; RR = 0.45, 95% CI 0.32, 0.62; RD = 4.9 pp), disability (20.9% versus 30.2%; RR = 0.65, 95% CI 0.53, 0.79; RD = 9.3 pp), and death (1.8% versus 3.1%; RR = 0.52, 95% CI 0.28, 0.96; RD = 1.3 pp). | In this study, a primary care-based mobile health intervention integrating provider-centered and patient-facing technology was effective in reducing BP and improving stroke secondary prevention in a resource-limited rural setting in China |
| Yoo et al., 2022 (97) | Register-based, retrospective cohort study  Control n=8337, intervention n=1298 | Intervention: Group 1 Physiotherapy whole; disorder of central nervous system (DCNS), mattress or mobilisation training (MMT), and Gait training (GT) or occupational therapy (OT) whole: CO, special OT (SO), activities of daily living training (ADLT), and rehabilitative dysphagia therapy (RDT); Group 2 PT: MMT and GT + OT whole; Group 3: PT whole + OT whole; Group 4: PT: DCNS and GT + OT whole. DCNS = >120 hours of PT | Mortality | Results of the Cox multivariate analysis of survival according to the treatment received:  Control (no treatment): HR = 1.000 (reference)  PT whole or OT whole: 1.348 (0.959–1.896), p=0.0856  PT: MMT and GT + OT whole: 1.538 (0.718–3.293), p-0.2682  PT whole + OT whole: 1.272 (0.910–1.778), p=0.1584  PT: DNCS and GT + OT whole: 0.361 (0.169–0.769), p=0.0082 | Patients with stroke who received both PT and OT had a better long-term prognosis than those who received either treatment alone. Therapy performed by a physical therapist with more than 120 hours of training effectively improved the patients’ long-term prognosis. Patients who received CO, SO, ADLT, and RDT in addition to DCNS and GT had a lower mortality rate (HR: 0.361, 95% CI: 0.169–0.769) than those who did not receive intensive treatment. Patients who received >10 sessions of DCNS per month had significantly lower mortality rates at 3 (HR: 0.709, 95% CI: 0.564–0.891) and 6 months (HR: 0.752, 95% CI: 0.587–0.962) post-stroke than those who received < 10 treatment sessions per month |
| Yu et al., 2019 (98) | Randomised controlled trial  N=242 (Control n=121, int n=121) | Intensive patient care program (IPCP) on cognitive impairment, anxiety, depression including comprehensive psychoeducation and psychonursing, cognitive rehabilitation training and mobile communication application (psychological, mhealth) | Cognitive impairment, anxiety, depression, relapse-free survival | IPCP increased MMSE score at M12 and change of MMSE score (M12-M0), while decreased cognitive impairment rate at M12. For anxiety, decreased change of HADS-A score (M12-M0) and lower anxiety rate at M12 were observed in IPCP group compared to control group. For depression, decreased HADS-D score at M6 and M12, reduced change of HADS-D score (M12-M0) and lower depression rate at M12 were shown in IPCP group compared to control group. Besides, RFS was numerically longer in IPCP group compared to control group, but without statistical signiﬁcance. | IPCP presents with a positive inﬂuence on improving cognitive impairment and decreasing anxiety as well as depression, while a less eﬀect on improving RFS in AIS patients |
| Zhang et al., 2019 (99) | Randomised controlled trial  N=196 (Control n=98, int n=98) | Intensive caregiver education program (ICEP) comprising intensive individualized education for patients and caregivers and psychological nursing for caregivers delivered by nurses in 1:1 sessions, caregivers received repeat sessions every 2 weeks | Patients’ cognitive function, anxiety, and depression | Cognitive impairment score at M12 and cognitive impairment score change (M12–M0) were increased, while cognitive impairment rate at M12 was reduced in the ICEP group compared with the Control group. Anxiety score change (M12–M0), anxiety score at M12, and anxiety rate at M12 were decreased in the ICEP group compared with the Control group. Depression score change (M12–M0), depression score at M12, and depression rate at M12 were lower in the ICEP group compared with the Control group. Further subgroup analysis based on baseline features also provided similar results. | In conclusion, ICEP effectively reduced cognitive impairment, anxiety, and depression in AIS patients. |

# References

1. Abdul Aziz Aznida Furzah, Azlin MNN, Amrizal MN, Saperi S, Mohamed AS. The integrated care pathway for managing post stroke patients (iCaPPS © ) in public primary care Healthcentres in Malaysia: impact on quality adjusted life years (QALYs) and cost effectiveness analysis. BMC geriatrics. 2020;20(1):70.

2. Ahmadi M, Laumeier I, Ihl T, Steinicke M, Ferse C, Endres M, et al. A support programme for secondary prevention in patients with transient ischaemic attack and minor stroke (INSPiRE-TMS): an open-label, randomised controlled trial. The lancet Neurology. 2020;19(1):49‐60.

3. Demir Avci Y, Gözüm S. Effects of Transitional Care Model-Based Interventions for Stroke Patients and Caregivers on Caregivers' Competence and Patient Outcomes: Randomized Controlled Trial. Computers, informatics, nursing : CIN. 2023;41(10):805-14.

4. Bath PM, Woodhouse LJ, Appleton JP, Beridze M, Christensen H, Dineen RA, et al. Triple versus guideline antiplatelet therapy to prevent recurrence after acute ischaemic stroke or transient ischaemic attack: the TARDIS RCT. Health Technol Assess. 2018;22(48):1-76.

5. Bergh E, Askim T, Rønning OM, Šaltyteė Benth J, Fjærtoft H, Thommessen B. Does implementation of a standardized pathway of stroke care affect functional outcome after stroke? International journal of stroke : official journal of the International Stroke Society. 2023;18(5):578-85.

6. Bernhardt J, Langhorne P, Lindley RI, Thrift AG, Ellery F, Collier J, et al. Efficacy and safety of very early mobilisation within 24 h of stroke onset (AVERT): a randomised controlled trial. Lancet. 2015;386(9988):46-55.

7. Bodechtel U, Barlinn K, Helbig U, Arnold K, Siepmann T, Pallesen L-P, et al. The stroke east Saxony pilot project for organized post-stroke care: a case-control study. Brain and behavior. 2016;6(5):e00455.

8. Bragstad LK, Hjelle EG, Zucknick M, Sveen U, Thommessen B, Bronken BA, et al. The effects of a dialogue-based intervention to promote psychosocial well-being after stroke: a randomized controlled trial. Clinical rehabilitation. 2020;34(8):1056‐71.

9. Brandal A, Eriksson E, Glader, EL, Wester P. Effect of early supported discharge after stroke on patient reported outcome based on the Swedish Riksstroke registry. BMC NEUROLOGY. 2019;19.

10. Brouns B, Van Bodegom-Vos L, De Kloet AJ. Tamminga SJ, Volker G, Berger MAM, et al. Effect of a Comprehensive erehabilitation Intervention Alongside Conventional Stroke Rehabilitation on Disability and Health-related Quality of Life: a Pre-post comparison. Journal of Rehabilitation Medicine (Stiftelsen Rehabiliteringsinformation). 2021;53(3):1-12.

11. Brouwer B, Bryant D, Garland SJ. Effectiveness of Client-Centered "Tune-Ups" on Community Reintegration, Mobility, and Quality of Life After Stroke: a Randomized Controlled Trial. Archives of physical medicine and rehabilitation. 2018;99(7):1325‐32.

12. Buckley BJR, Harrison SL, Fazio-Eynullayeva E, Underhill P, Lane DA, Thijssen DHJ, et al. Exercise-Based Cardiac Rehabilitation Associates with Lower Major Adverse Cardiovascular Events in People with Stroke. Cerebrovasc Dis. 2022;51(4):488-92.

13. Bushnell CD, Kucharska-Newton AM, Jones SB, Psioda MA, Johnson AM, Daras LC, et al. Hospital Readmissions and Mortality Among Fee-for-Service Medicare Patients With Minor Stroke or Transient Ischemic Attack: findings From the COMPASS Cluster-Randomized Pragmatic Trial. Journal of the American Heart Association. 2021;10(23):e023394.

14. Cecchi F, Diverio M, Arienti C, Corbella E, Marrazzo F, Speranza G, et al. Development and implementation of a stroke rehabilitation integrated care pathway in an Italian no profit institution: an observational study. EUROPEAN JOURNAL OF PHYSICAL AND REHABILITATION MEDICINE. 2020;56(6):713-24.

15. Chang K-C, Hung J-W, Lee H-C, Yen C-L, Wu C-Y, Yang C-L, et al. Rehabilitation Reduced Readmission and Mortality Risks in Patients With Stroke or Transient Ischemic Attack: A Population-based Study. Medical care. 2018;56(4):290-8.

16. Chang K-V, Chen K-H, Chen Y-H, Lien W-C, Chang W-H, Lai C-L, et al. A multicenter study to compare the effectiveness of the inpatient post acute care program versus traditional rehabilitation for stroke survivors. Scientific reports. 2022;12(1):12811.

17. Chen L, Sit JW-H, Shen X. Quasi-experimental evaluation of a home care model for patients with stroke in China. Disability & Rehabilitation. 2016;38(23):2271-6.

18. Chen CM, Yang YH, Chang CH, Chen PC. Effects of Transferring to the Rehabilitation Ward on Long-Term Mortality Rate of First-Time Stroke Survivors: A Population-Based Study. Archives of physical medicine and rehabilitation. 2017;98(12):2399-407.

19. Cheng C, Liu X, Fan W, Bai X, Liu Z. Comprehensive Rehabilitation Training Decreases Cognitive Impairment, Anxiety, and Depression in Poststroke Patients: A Randomized, Controlled Study. Journal of stroke and cerebrovascular diseases : the official journal of National Stroke Association. 2018;27(10):2613-22.

20. Cheng EM, Cunningham WE, Towfighi A, Sanossian N, Bryg RJ, Anderson TL, et al. Efficacy of a Chronic Care-Based Intervention on Secondary Stroke Prevention Among Vulnerable Stroke Survivors: a Randomized Controlled Trial. Circulation Cardiovascular quality and outcomes. 2018;11(1):e003228.

21. Chiu C-C, Lin H-F, Lin C-H, Chang H-T, Hsien H-H, Hung K-W, et al. Multidisciplinary Care after Acute Care for Stroke: A Prospective Comparison between a Multidisciplinary Post-Acute Care Group and a Standard Group Matched by Propensity Score. International journal of environmental research and public health. 2021;18(14).

22. Chu K, Bu X, Sun Z, Wang Y, Feng W, Xiao, L, et al. Feasibility of a Nurse-Trained, Family Member-Delivered Rehabilitation Model for Disabled Stroke Patients in Rural Chongqing, China. Journal of stroke and cerebrovascular diseases : the official journal of National Stroke Association. 2020;29(12):105382.

23. Cuccurullo SJ, Fleming TK, Kostis JB, Greiss C, Eckert A, Ray AR, et al. Impact of Modified Cardiac Rehabilitation Within a Stroke Recovery Program on All-Cause Hospital Readmissions. American journal of physical medicine & rehabilitation. 2022;101(1):40-7.

24. Cuccurullo SJ, Fleming TK, Zinonos S, Cosgrove NM, Cabrera J, Kostis JB, et al. Stroke Recovery Program with Modified Cardiac Rehabilitation Improves Mortality, Functional & Cardiovascular Performance. Journal of stroke and cerebrovascular diseases : the official journal of National Stroke Association. 2022;31(5):106322.

25. Cuccurullo SJ, Fleming TK, Kostis WJ, Greiss C, Gizzi MS, Eckert A, et al. Impact of a Stroke Recovery Program Integrating Modified Cardiac Rehabilitation on All-Cause Mortality, Cardiovascular Performance and Functional Performance. American Journal of Physical Medicine & Rehabilitation. 2019;98(11):953-63.

26. Cumming TB, Churilov L, Collier J, Donnan G, Ellery F, Dewey H, et al. Early mobilization and quality of life after stroke: Findings from AVERT. Neurology. 2019;93(7):e717-e28.

27. de Belvis AG, Lohmeyer FM, Barbara A, Giubbini G, Angioletti C, Frisullo G, et al. Ischemic stroke: clinical pathway impact. INTERNATIONAL JOURNAL OF HEALTH CARE QUALITY ASSURANCE. 2019;32(3):588-98.

28. Deng A, Yang S, Xiong R. Effects of an integrated transitional care program for stroke survivors living in a rural community: a randomized controlled trial. Clinical rehabilitation. 2020;34(4):524-32.

29. Dello S, Lemmens R, Demeestere J, Michiels D, Wellens L, Weltens C, et al. A nurse-led multicomponent intervention supported by advanced electronic health records to improve the acute management of stroke patients: A pre- and post-intervention study. INTERNATIONAL JOURNAL OF NURSING STUDIES ADVANCES. 2021;3.

30. Deutschbein J, Grittner U, Schneider A, Schenk L. Community care coordination for stroke survivors: results of a complex intervention study. BMC health services research. 2020;20(1):1143.

31. Døhl Ø, Halsteinli V, Askim T, Gunnes M, Ihle-Hansen H, Indredavik B, et al. Factors contributing to post-stroke health care utilization and costs, secondary results from the life after stroke (LAST) study. BMC health services research. 2020;20(1):288.

32. Duncan PW, Bushnell CD, Jones SB, Psioda MA, Gesell SB, D'Agostino RB, et al. Randomized Pragmatic Trial of Stroke Transitional Care: The COMPASS Study. Circulation Cardiovascular quality and outcomes. 2020;13(6):e006285.

33. Ellis-Hill C, Thomas S, Gracey F, Lamont-Robinson C, Cant R, et al. HeART of Stroke: randomised controlled, parallel-arm, feasibility study of a community-based arts and health intervention plus usual care compared with usual care to increase psychological well-being in people following a stroke. BMJ open. 2019;9(3):e021098.

34. Feng XH, Deng AW, Chen JH, Xiong RB. Health-related outcomes 6 months after integrated care of older people with stroke in 2 different primary care settings. FAMILY PRACTICE. 2023.

35. Feng W, Yu H, Wang J, Xia J. Application effect of the hospital-community integrated service model in home rehabilitation of stroke in disabled elderly: a randomised trial. Annals of palliative medicine. 2021;10(4):4670-7.

36. Freburger JK, Pastva AM, Coleman SW, Peter KM, Kucharska-Newton AM, Johnson AM, et al. Skilled Nursing and Inpatient Rehabilitation Facility Use by Medicare Fee-for-Service Beneficiaries Discharged Home After a Stroke: Findings From the COMPASS Trial. Archives of physical medicine and rehabilitation. 2022;103(5):882.

37. Fu V, Weatherall M, McPherson K, Taylor W, McRae A, Thomson T, et al. Taking Charge after Stroke: A randomized controlled trial of a person-centered, self-directed rehabilitation intervention. International journal of stroke : official journal of the International Stroke Society. 2020;15(9):954-64.

38. Gao J, Lin M, Zhao J, Bi S, Ni Z, Shang X. Different interventions for post-ischaemic stroke depression in different time periods: a single-blind randomized controlled trial with stratification by time after stroke. Clinical rehabilitation. 2017;31(1):71-81.

39. Geng G, He W, Ding L, Klug D, Xiao Y. Impact of transitional care for discharged elderly stroke patients in China: an application of the Integrated Behavioral Model. TOPICS IN STROKE REHABILITATION. 2019;26(8):621-9.

40. Graven C, Brock K, Hill KD, Cotton S, Joubert L. First Year After Stroke: An Integrated Approach Focusing on Participation Goals Aiming to Reduce Depressive Symptoms. Stroke. 2016;47(11):2820-7.

41. Greger J, Wojcik R, Westphal E, Aladeen T, Landolf K, Boyce S, et al. Pharmacist intervention and anti-platelet medication monitoring in patients following stroke and transient ischemic attack. JOURNAL OF THE AMERICAN COLLEGE OF CLINICAL PHARMACY. 2021;4(3):311-7.

42. Gun Young Yang, Min HS. The Effects of an Integrated Management Program on Physical Function, Cognitive Function, and Depression in Patients with Subacute stroke. Journal of Korean Critical Care Nursing. 2021;14(1):50-62.

43. He Y, Wang R, Dong S, Long S, Zhang P, Feng L. Nurse-led rapid rehabilitation following mechanical thrombectomy in patients with acute ischemic stroke: A historical control study. Medicine. 2023;102(28):e34232.

44. Hjelle EG, Bragstad LK, Kirkevold M, Zucknick M, Bronken BA, Martinsen R, et al. Effect of a dialogue-based intervention on psychosocial well-being 6 months after stroke in Norway: A randomized controlled trial. Journal of rehabilitation medicine. 2019;51(8):557-65.

45. Huang J, Zuo J, Tang X, Zou J, Zeng Y, Chen S, et al. Early Rehabilitation and Nursing Intervention (ERNI) Accelerates the Recovery of Patients With Ischemic Stroke. The neurologist. 2023;28(6):409-12.

46. Jones F, Gage H, Drummond A, Bhalla A, Grant R, Lennon S, et al. Feasibility study of an integrated stroke self-management programme: a cluster-randomised controlled trial. BMJ OPEN. 2016;6(1).

47. Joubert J, Davis SM, Donnan GA, Levi C, Gonzales G, Joubert L, et al. ICARUSS: An effective model for risk factor management in stroke survivors. INTERNATIONAL JOURNAL OF STROKE. 2020;15(4):438-53.

48. Jung S-H, Park E, Kim J-H, Park, B-A, Yu J-W, et al. Effects of Self RehAbilitation Video Exercises (SAVE) on Functional Restorations in Patients with Subacute Stroke. HEALTHCARE. 2021;9(5).

49. Kalav S, Bektas H, Ünal A. Effects of Chronic Care Model‐based interventions on self‐management, quality of life and patient satisfaction in patients with ischemic stroke: A single‐blinded randomized controlled trial. Japan Journal of Nursing Science. 2022;19(1):1-18.

50. Kam Yuet Wong F, Wang SL, Ng SSM, Lee PH, Wong AKC, Li H, et al. Effects of a transitional home-based care program for stroke survivors in Harbin, China: a randomized controlled trial. Age Ageing. 2022;51(2).

51. Khramov W, Kogaeva KP, Arkhipova, LU, Alekseeva, VO, Lukyanova, MI. EFFECTIVENESS OF POST-STROKE SOCIAL REHABILITATION IN PATIENTS WITH MODERATE IMPAIRMENTS. BULLETIN OF RUSSIAN STATE MEDICAL UNIVERSITY. 2021(2):62-8.

52. Koch S, Tiozzo E, Simonetto M, Loewenstein D, Wright CB, Dong C, et al. Randomized Trial of Combined Aerobic, Resistance, and Cognitive Training to Improve Recovery From Stroke: Feasibility and Safety. Journal of the American Heart Association. 2020;9(10):e015377.

53. Langhorne P, Wu O, Rodgers H, Ashburn A, Bernhardt J. A Very Early Rehabilitation Trial after stroke (AVERT): a Phase III, multicentre, randomised controlled trial. Health Technol Assess. 2017;21(54):1-120.

54. Lee A-Y, Park S-A, Park H-G, Son K-C. Determining the Effects of a Horticultural Therapy Program for Improving the Upper Limb Function and Balance Ability of Stroke Patients. HORTSCIENCE. 2018;53(1):110-9.

55. Lewthwaite R, Winstein CJ, Lane CJL, Blanton S, Wagenheim BR, Nelsen MA, Dromerick AW, et al. Accelerating Stroke Recovery: Body Structures and Functions, Activities, Participation, and Quality of Life Outcomes From a Large Rehabilitation Trial. Neurorehabilitation & Neural Repair. 2018;32(2):150-65.

56. Li Y, Wang Q, Liu XL, Hui R, Zhang YP. Effect of the physical rehabilitation program based on self-care ability in patients with acute ischemic stroke: a quasi-experimental study. FRONTIERS IN NEUROLOGY. 2023;14.

57. Lin Y. EFFECT OF MULTI-DISCIPLINARY CLINICAL NURSING PATHWAY ON SELF-EFFICACY AND SELF-MANAGEMENT BEHAVIOR IN PATIENTS WITH CEREBRAL INFARCTION. ACTA MEDICA MEDITERRANEA. 2022;38(5):3133-8.

58. Lin RC, Chiang SL, Heitkemper MM, Weng, SM, Lin CF, Yang FC, et al. Effectiveness of Early Rehabilitation Combined With Virtual Reality Training on Muscle Strength, Mood State, and Functional Status in Patients With Acute Stroke: A Randomized Controlled Trial. Worldviews on Evidence-Based Nursing. 2020;17(2):158-67.

59. Liu N, Cadilhac DA, Andrew NE, Zeng L, Li Z, Li J, et al. Randomized controlled trial of early rehabilitation after intracerebral hemorrhage stroke: difference in outcomes within 6 months of stroke. Stroke (00392499). 2014;45(12):3502-7.

60. Liu LM. EFFECTS OF TRANSITIONAL CARE ON FUNCTIONAL EXERCISE: COMPLIANCE AND HEALTH STATUS OF STROKE PATIENTS. ACTA MEDICA MEDITERRANEA. 2018;34(4):959-65.

61. Lo SHS, Chau JPC, Lau AYL, Choi KC, Shum EWC, Lee VWY, et al. Virtual Multidisciplinary Stroke Care Clinic for Community-Dwelling Stroke Survivors: A Randomized Controlled Trial. Stroke. 2023;54(10):2482-90.

62. López-Liria R, Vega-Ramírez FA, Rocamora-Pérez P, Aguilar-Parra JM, Padilla-Góngora D. Comparison of Two Post-Stroke Rehabilitation Programs: A Follow-Up Study among Primary versus Specialized Health Care. PloS one. 2016;11(11):e0166242.

63. Lu LX, Wei SY, Huang QY, Chen YK, Huang FX, Ma XA, et al. Original Article Effect of "Internet plus tertiary hospital-primary hospital-family linkage home care" model on self-care ability and quality of life of discharged stroke patients. AMERICAN JOURNAL OF TRANSLATIONAL RESEARCH. 2023;15(12):6727-39.

64. Luengo-Fernandez R, Li L, Silver L, Gutnikov S, Beddows NC, Rothwell PM. Long-Term Impact of Urgent Secondary Prevention After Transient Ischemic Attack and Minor Stroke: Ten-Year Follow-Up of the EXPRESS Study. Stroke. 2022;53(2):488-96.

65. MacKay-Lyons M, Gubitz G, Phillips S, Giacomantonio N, Firth W, Thompson K, et al. Program of Rehabilitative Exercise and Education to Avert Vascular Events After Non-Disabling Stroke or Transient Ischemic Attack (PREVENT Trial): a Randomized Controlled Trial. Neurorehabilitation and neural repair. 2022;36(2):119‐30.

66. Man S, Zhao X, Uchino K, Hussain MS, Smith EE, Bhatt DL, et al. Comparison of Acute Ischemic Stroke Care and Outcomes Between Comprehensive Stroke Centers and Primary Stroke Centers in the United States. Circulation Cardiovascular quality and outcomes. 2018;11(6):e004512.

67. Markle-Reid M, Valaitis R, Bartholomew A, Fisher K, Fleck R, Ploeg J, et al. An integrated hospital-to-home transitional care intervention for older adults with stroke and multimorbidity: A feasibility study. Journal of Comorbidity. 2020;10:1-21.

68. Minshall C, Castle DJ, Thompson DR, Pascoe M, Cameron J, McCabe M, et al. A psychosocial intervention for stroke survivors and carers: 12-month outcomes of a randomized controlled trial. TOPICS IN STROKE REHABILITATION. 2020;27(8):563-76.

69. Mofidi R, Thomas M, Wong PF, Bergin A, Young G. Do Integrated Systems of Stroke Care Improve Symptom to Surgery Times in Patients with Symptomatic Carotid Stenosis? A Single Centre Decision Tree Analysis. Eur J Vasc Endovasc Surg. 2018;56(6):784-92.

70. Mohammadi E, Hassandoost F, Mozhdehipanah H. Evaluation of the "partnership care model" on quality of life and activity of daily living in stroke patients: A randomized clinical trial. Japan journal of nursing science : JJNS. 2022;19(1):e12448.

71. Nakibuuka J, Sajatovic M, Nankabirwa J, Ssendikadiwa C, Kalema N, Kwizera A, et al. Effect of a 72 Hour Stroke Care Bundle on Early Outcomes after Acute Stroke: A Non Randomised Controlled Study. PLOS ONE. 2016;11(5).

72. Nayeri ND, Mohammadi S, Razi SP, Kazemnejad A. Investigating the effects of a family-centered care program on stroke patients' adherence to their therapeutic regimens. Contemporary nurse. 2014;47(1-2):88-96.

73. Nguyen-Huynh MN, Klingman JG, Avins AL, Rao VA, Eaton A, Bhopale S, et al. Novel Telestroke Program Improves Thrombolysis for Acute Stroke Across 21 Hospitals of an Integrated Healthcare System. Stroke. 2018;49(1):133-9.

74. Olaiya MT, Kim J, Nelson MR, Srikanth VK, Bladin CF, Gerraty RP, et al. Effectiveness of a shared team approach between nurses and doctors for improved risk factor management in survivors of stroke: a cluster randomized controlled trial. Eur J Neurol. 2017;24(7):920-8.

75. Pedapati R, Bhatia R, Shakywar M, Gupta A, Vishnubhatla S, Srivastava MVP, et al. Educating Caregivers to Reduce Complications and Improve Outcomes of Stroke Patients (ECCOS)-A Cluster-Randomized Trial. JOURNAL OF STROKE & CEREBROVASCULAR DISEASES. 2021;30(9).

76. Peng LN, Lu WH, Liang CK, Chou MY, Chung CP, Tsai SL, et al. Functional Outcomes, Subsequent Healthcare Utilization, and Mortality of Stroke Postacute Care Patients in Taiwan: A Nationwide Propensity Score-matched Study. Journal of the American Medical Directors Association. 2017;18(11):990.e7-.e12.

77. Rafsten L, Danielsson A, Nordin A, Björkdahl A, Lundgren-Nilsson A, Larsson M, et al. Gothenburg Very Early Supported Discharge study (GOTVED): a randomised controlled trial investigating anxiety and overall disability in the first year after stroke. BMC neurology. 2019;19(1):277.

78. Rasmussen RS, Østergaard A, Kjær P, Skerris A, Skou C, Christoffersen J, et al. Stroke rehabilitation at home before and after discharge reduced disability and improved quality of life: a randomised controlled trial. Clinical Rehabilitation. 2016;30(3):225-36.

79. Reeves MJ, Fritz MC, Woodward AT, Hughes AK, Coursaris CK, Swierenga SJ, et al. Michigan Stroke Transitions Trial. Circulation Cardiovascular quality and outcomes. 2019;12(7):e005493.

80. Rodgers H, Howel D, Bhattarai N, Cant R. Drummond A, Ford GA, et al. Evaluation of an Extended Stroke Rehabilitation Service (EXTRAS): A Randomized Controlled Trial and Economic Analysis. STROKE. 2019;50(12):3561-8.

81. Saal S, Becker C, Lorenz S, Schubert M, Kuss O, Stang A, et al. Effect of a stroke support service in Germany: a randomized trial. TOPICS IN STROKE REHABILITATION. 2015;22(6):429-36.

82. Schwarzbach CJ, Eichner FA, Rücker V, Hofmann AL, Keller M, Audebert HJ, et al. The structured ambulatory post-stroke care program for outpatient aftercare in patients with ischaemic stroke in Germany (SANO): an open-label, cluster-randomised controlled trial. Lancet Neurol. 2023;22(9):787-99.

83. Sharma M, Hart RG, Connolly SJ, Bosch J, Shestakovska O, Ng KKH, et al. Stroke Outcomes in the COMPASS Trial. Circulation. 2019;139(9):1134-45.

84. Shaw L, Bhattarai N, Cant R, Drummond A, Ford GA, Forster A, et al. An extended stroke rehabilitation service for people who have had a stroke: the EXTRAS RCT. HEALTH TECHNOLOGY ASSESSMENT. 2020;24(24):1-+.

85. Swanson JO, Moger TA. Comparisons of readmissions and mortality based on post-discharge ambulatory follow-up services received by stroke patients discharged home: a register-based study. BMC health services research. 2019;19(1):4.

86. Teuschl Y, Matz K, Firlinger B, Dachenhausen A, Tuomilehto J, Brainin M, et al. Preventive effects of multiple domain interventions on lifestyle and risk factor changes in stroke survivors: Evidence from a two-year randomized trial. Int J Stroke. 2017;12(9):976-84.

87. Towfighi A, Cheng EM, Ayala-Rivera M, Barry F, McCreath H, Ganz D, et al. Secondary Stroke Prevention, Unitin. Effect of a Coordinated Community and Chronic Care Model Team Intervention vs Usual Care on Systolic Blood Pressure in Patients With Stroke or Transient Ischemic Attack The SUCCEED Randomized Clinical Trial. JAMA NETWORK OPEN. 2021;4(2).

88. Tung YJ, Lin WC, Lee LF, Lin HM, Ho CH, Chou W. Comparison of Cost-Effectiveness between Inpatient and Home-Based Post-Acute Care Models for Stroke Rehabilitation in Taiwan. INTERNATIONAL JOURNAL OF ENVIRONMENTAL RESEARCH AND PUBLIC HEALTH. 2021;18(8).

89. Vluggen TPMM, van Haagstreg, JCM. Tan FE, Verbunt JA, van Heugten CM, Schols, JMG. A. Effectiveness of an integrated multidisciplinary geriatric rehabilitation programme for older persons with stroke: a multicentre randomised controlled trial. BMC geriatrics. 2021;21(1):134.

90. Wang J, Wang J, Qiu S, Zhou C, Zhang H, Li Q, et al. Pharmaceutical care program for ischemic stroke patients: a randomized controlled trial. Int J Clin Pharm. 2021;43(5):1412-9.

91. Long Weijan, Zhang J. Patient Caregiver Comprehensive Rehabilitation Nursing Training Joint Continued nursing care for ischemic stroke patients at home Impact on quality of life and activities of daily living. Chinese Nursing Research. 2017;31(20):2456-61.

92. Willeit P, Toell T, Boehme C, Krebs S, Mayer L, Lang C, et al. STROKE-CARD care to prevent cardiovascular events and improve quality of life after acute ischaemic stroke or TIA: A randomised clinical trial. EClinicalMedicine. 2020;25:100476.

93. Wong FKY, Yeung SM. Effects of a 4-week transitional care programme for discharged stroke survivors in Hong Kong: a randomised controlled trial. Health & Social Care in the Community. 2015;23(6):619-31.

94. Wu Z, Xu J, Yue C, Li Y, Liang Y. Collaborative Care Model Based Telerehabilitation Exercise Training Program for Acute Stroke Patients in China: A Randomized Controlled Trial. Journal of stroke and cerebrovascular diseases : the official journal of National Stroke Association. 2020;29(12):105328.

95. Xi W, Duan Y, Wang A. Influence of continuing nursing outside hospital on rehabilitation effect and quality of life in patients with stroke. Chinese Nursing Research. 2017;31(29):3760-2.

96. Yan LL, Gong E, Gu W, Turner EL, Gallis JA, Zhou Y, et al. Effectiveness of a primary care-based integrated mobile health intervention for stroke management in rural China (SINEMA): A cluster-randomized controlled trial. PLoS Med. 2021;18(4):e1003582.

97. Yoo D-Y, Choi J-K, Baek C-Y, Shin J-B. Impact of intensive rehabilitation on long-term prognosis after stroke: A Korean nationwide retrospective cohort study. Medicine. 2022;101(38):e30827.

98. Yu HL, Cao DX, Liu J. Effect of a novel designed intensive patient care program on cognitive impairment, anxiety, depression as well as relapse free survival in acute ischemic stroke patients: a randomized controlled study. NEUROLOGICAL RESEARCH. 2019;41(9):857-66.

99. Zhang L, Zhang T, Sun Y. A newly designed intensive caregiver education program reduces cognitive impairment, anxiety, and depression in patients with acute ischemic stroke. Braz J Med Biol Res. 2019;52(9):e8533.
