# Supplementary material for: Integrated care management for patients following acute stroke: a systematic review
Source: QJM. 2025 Jan 24;118(5):317–28. doi: 10.1093/qjmed/hcaf029 (PMC12341757; doi:10.1093/qjmed/hcaf029)

# Supplement S3. Risk of bias assessment (Randomised and non-randomised studies) Figure S1. Risk of bias according to ROB2 tool (randomised studies)


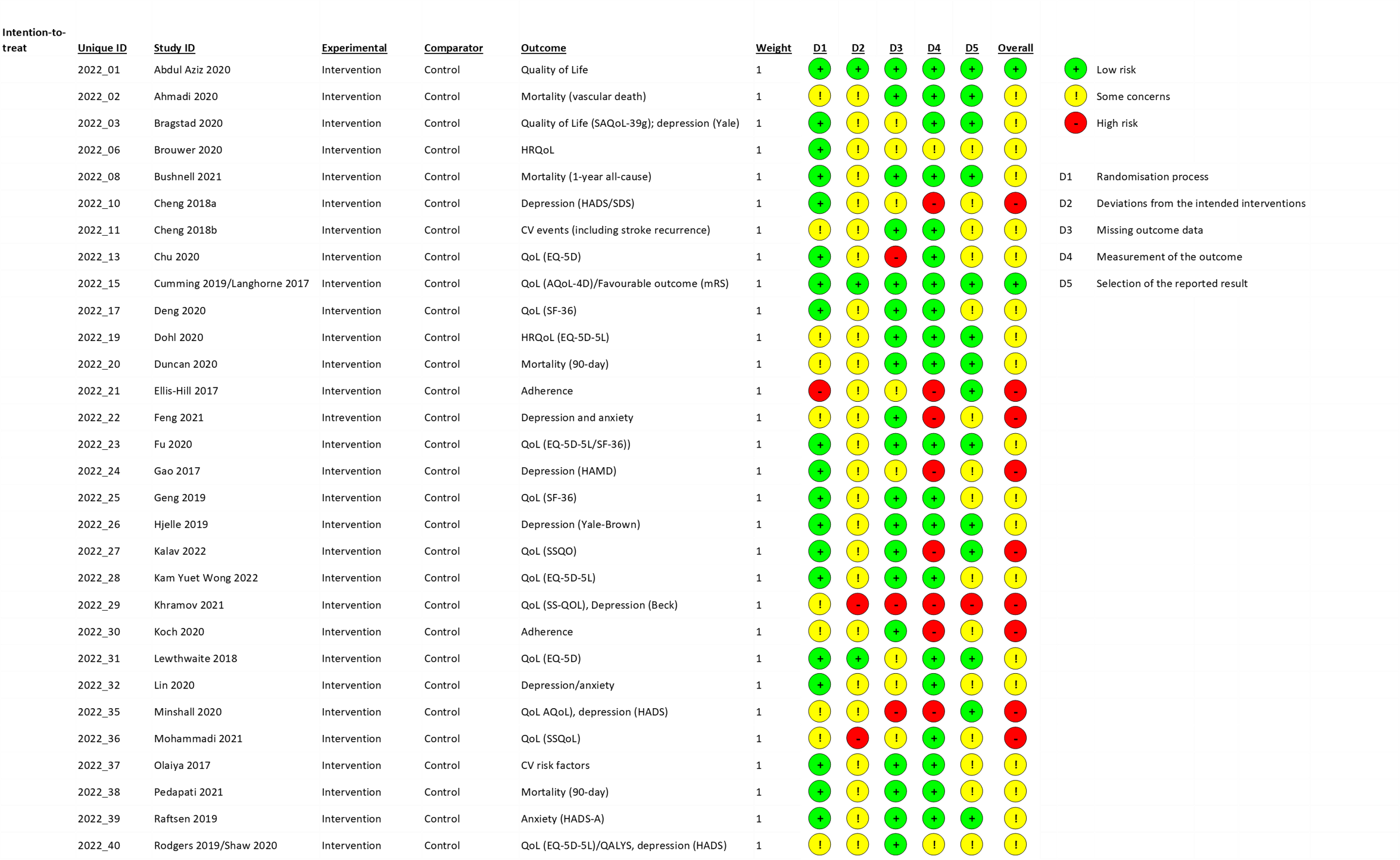


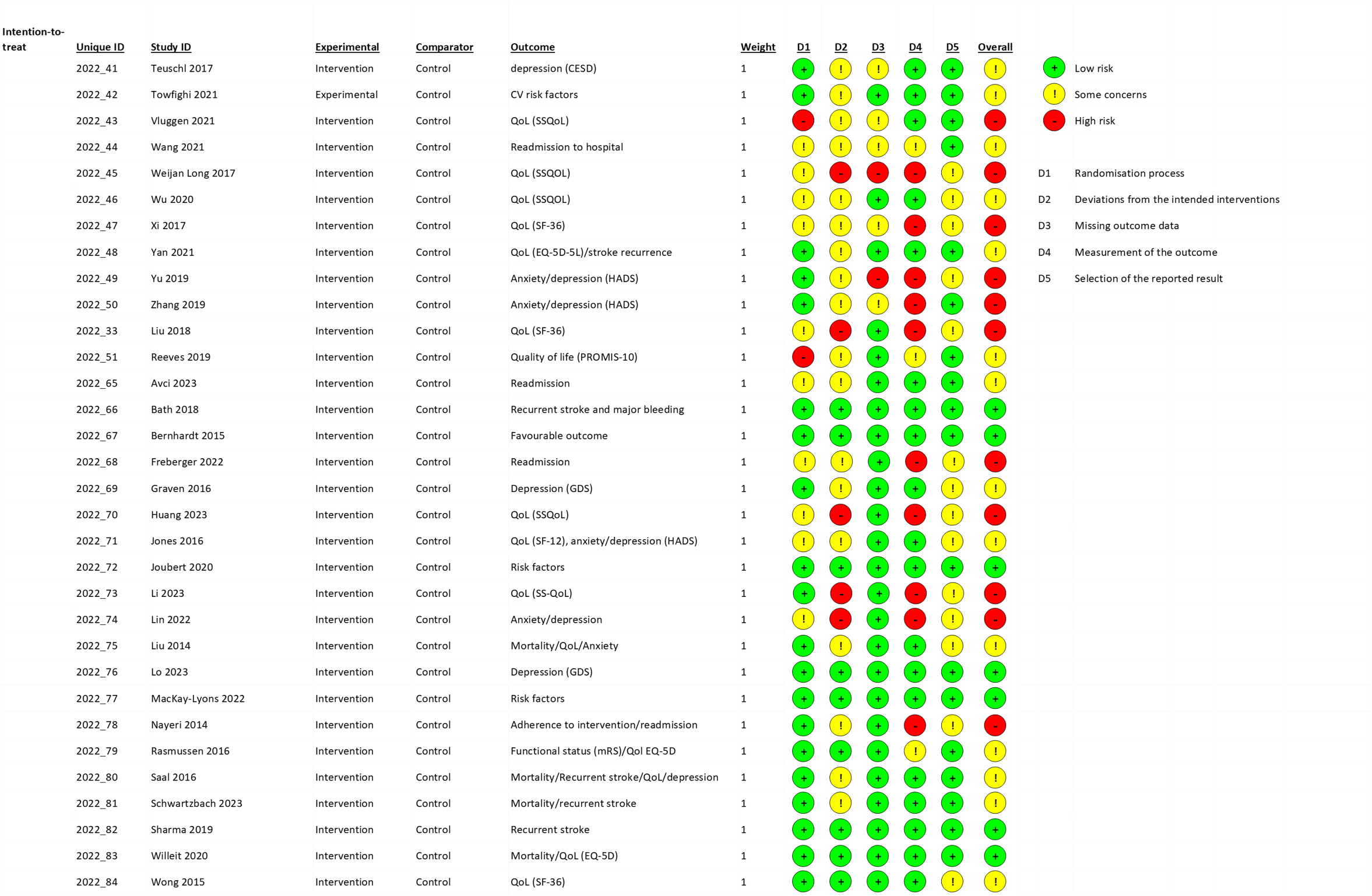


# Figure S2. Risk of bias according to ROBINS-I tool (nonrandomised studies)


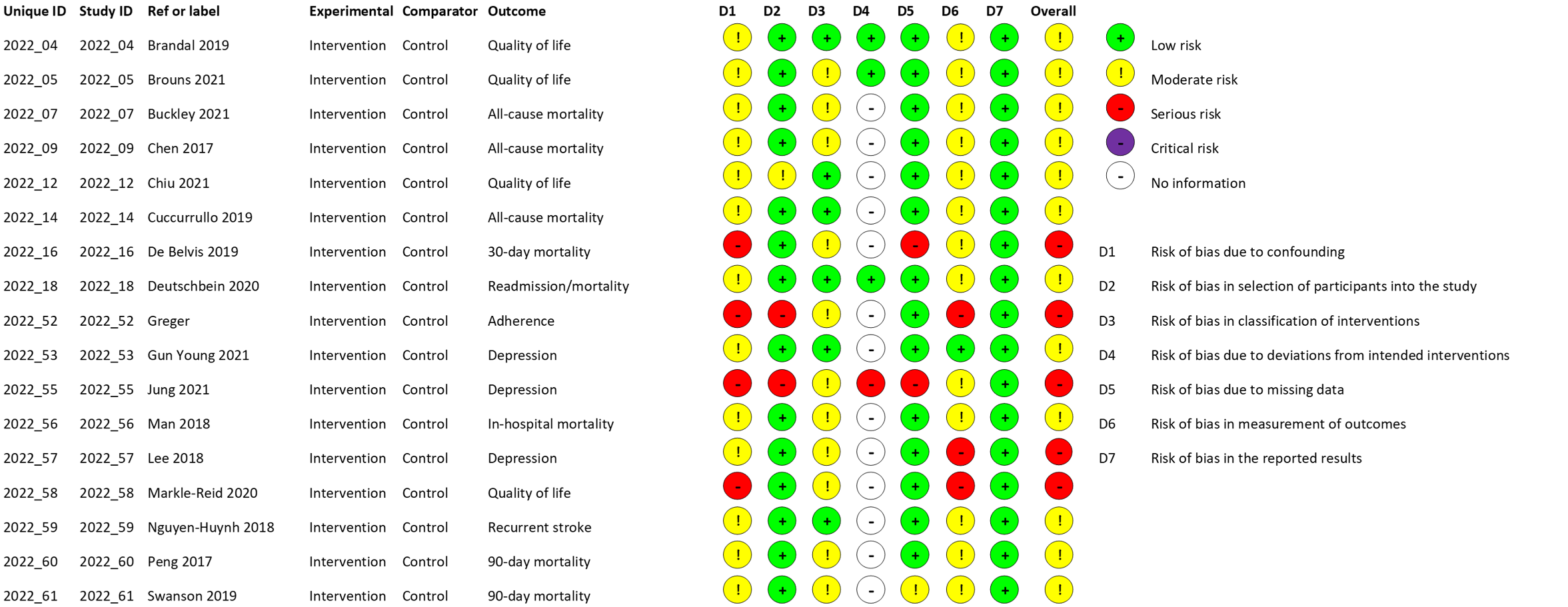


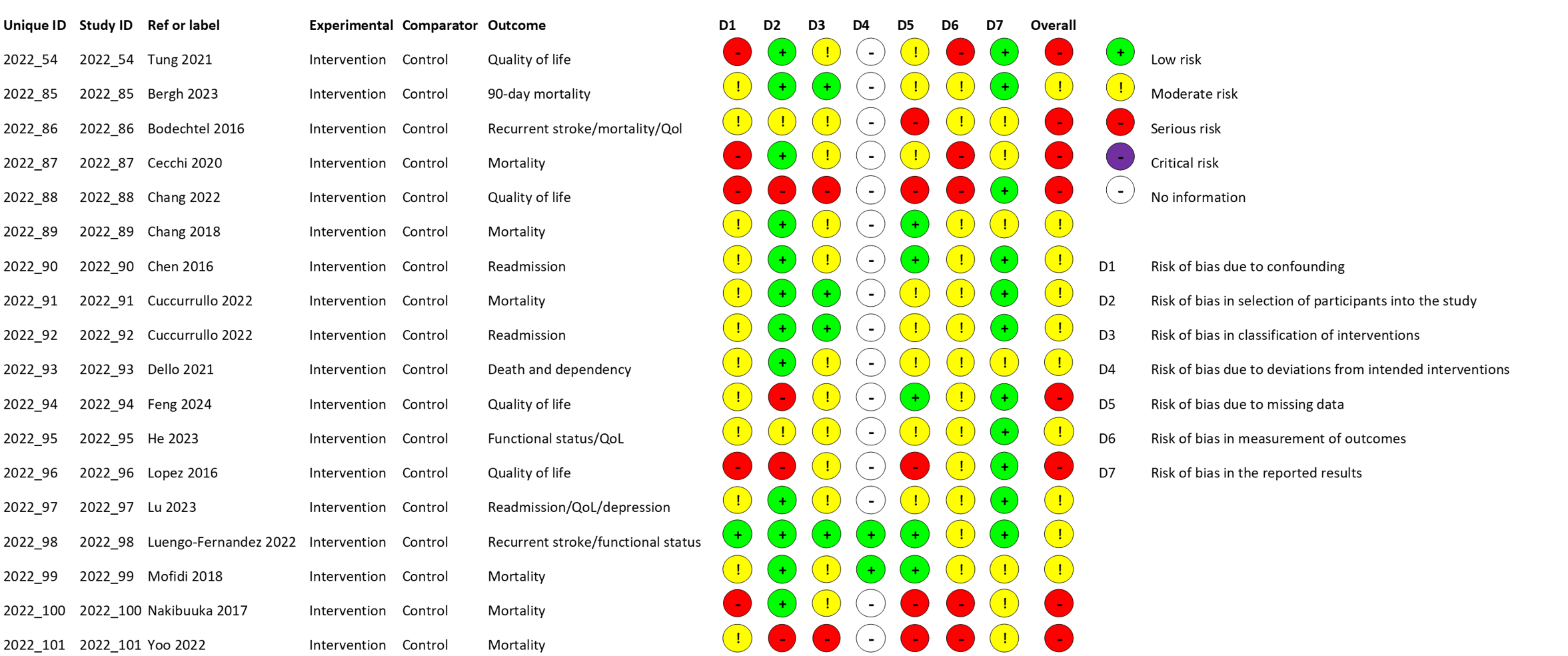

Supplement: hcaf029_Supplementary_Data [file hcaf029_supplementary_data.zip › hcaf029_Supplementary_Data/Supplement S3.docx]
