# Supplementary material for: Integrated care management for patients following acute stroke: a systematic review
Source: QJM. 2025 Jan 24;118(5):317–28. doi: 10.1093/qjmed/hcaf029 (PMC12341757; doi:10.1093/qjmed/hcaf029)
Supplement: hcaf029_Supplementary_Data [file hcaf029_supplementary_data.zip › hcaf029_Supplementary_Data/Supplement S4.docx]

# Supplement S4. Analysis of primary and secondary review outcomes

# Primary outcomes

## Mortality

Table S1: Analysis of mortality

| **Study details** | **Design/Patients** | **Intervention** | **Outcome** | **Results** | **Comments** |
| --- | --- | --- | --- | --- | --- |
| Ahmadi et al, 2020 (INSPiRE-TMS) (1) | Prospective, randomised, open­label, blinded endpoint, event-­driven trial  Control n=1042, intervention n=1030 | Counselling, lifestyle changes, medication adherence | Composite of vascular events, ACS, vascular mortality, all-cause mortality (2-year) | Vascular mortality (ITT): Intervention n=30 (2.9%), control n= 32 (3.1%) HR 0·94 (0·57–1·54)  All-cause mortality (ITT): Intervention n=73 (7·1%), control n=85 (8·2%) HR 0·85 (0·62–1·17) | No significant difference in primary outcome between groups |
| Bath et al., 2018 (TARDIS) (2) | International (UK, Denmark, Georgia, NZ) prospective randomised open-label blinded end-point superiority clinical trial  Guideline (control) n=1540, Intervention n=1556 | Intensive treatment group given combined aspirin (load 300mg, maintenance 50-150mg daily, typically 75mg), clopidogrel (load 300mg, maintenance 75mg daily), and dipyridamole (200mg twice daily modified release, given orally; or 100mg three or four times daily). | Secondary: death at 90 days | Events: Intervention 26/1556 (1.7%), control 28/1540 (1.8%) | No difference in mortality between groups |
| Bergh, 2023 (3) | Repeated measures analysis (pre-post) from a retrospective cohort of patients registered in the Norwegian Stroke Register (NSR).  N = 11009 included in study comparing functional outcome (5388 in 2017 and 5621 in 2019) | Standardised Care Pathway for stroke (SCP) based on National guidelines | Secondary: Mortality at 90 days | Overall, 1407 (16%) of 8785 patients had died within 90 days in 2017, and 1380 (15.3%) of 9028 in 2019, no significant difference between groups. | No significant improvement in function outcome after the introduction of an SCP. |
| Bernhardt et al., 2015 (AVERT) (4) | Single-blind randomised controlled trial in 56 acute stroke units in 5 countries (Aus, NZ, Malaysia, Singapore, UK -Eng, Scot, Wales, NI)  Control n=1050, intervention n=1054 | Very early mobilisation (exercise and movement therapy) | Secondary: Change in mRS, mortality | Mortality: Intervention 88/1048 (8%), control 72/1050 (7%), OR 1.34 (95% CI: 0.93-1.93), p=0.113 | Significantly fewer participants in the AVERT group had favourable outcome and there was no difference in mortality |
| Cuccurrullo et al., 2022 (5) | Prospective matched cohort study: feasibility study followed by matched group subanalysis  Control n=66, intervention n=76 | Enhanced SRP program including home visits, outpatient therapy and modifies cardiac rehabilitation | 1-year mortality | Mortality: Control 10/66 (15.2%), intervention 1/76 (1.3%), difference 13.9%, HR 0.11 (95% CI: 0.01-0.90), p=0.039 | Stroke survivors receiving a SRP integrating modified cardiac rehabilitation may potentially benefit from reductions in all-cause mortality and improvements in cardiovascular performance and function |
| Buckley et al., 2022 (6) | Retrospective observational study  N=836,923  Propensity matched cohorts:  Control n=2909, intervention n=2909 | Exercise-based cardiac rehabilitation | 2-year mortality | 8.5% Intervention, 16.5% control, (OR 0.47, 95% CI 0.40-0.56) | Significant (53%) reduction in mortality with intervention |
| Bushnell et al., 2021 (7) | COMPASS was a cluster-randomized trial of the effectiveness of the COMPASS-TC intervention (INV) compared with usual care (UC)  Participating hospitals: control n=20, inv n=20 Patients: Control n=1193, inv n=1069 | Post-acute care pathway (full pathway) follow-up with the advanced practice provider and nurse coordinator in clinic within 7 to 14 days post discharge, an individualized electronic care plan, and referral to community services | 1-year mortality | 8.5% intervention, 8.8% control (OR, 0.93; 95% CI, 0.68–1.26) | 7% reduction in mortality with intervention (not significant) |
| Chang et al., 2018 (8) | Retrospective cohort study using National Health Insurance database  Control n=2491, intervention 2103 | High intensity rehabilitation (inpatient, outpatient or both) versus no rehab | All-cause mortality (average follow-up time = 32 months) | Model 1  Control: 458/2491 (18.4%), low-intensity 250/1398 (17.9%), high intensity 103/705 (14.6%) (p=0.0062 high intensity vs control) | Rehabilitation use was associated with reduction of readmissions/mortality risks following stroke or TIA. |
| Chen, 2017 (9) | Retrospective cohort study  N=11,419  Propensity matched group n=390 (RR n=130, NtR n=130, NoR n=130) | Rehabilitation for hospitalised patients including mobilisation (exercise), speech therapy and dysphagia treatment | 5-year all-cause mortality | Rehab + rehab ward (RR) 17.7% Rehab no rehab ward (NtR) 26.9% (HR 2.2 95% CI 1.36-3.57; P<.01 vs RR) No rehab (NR) 47.7% (HR, 4.00; 95% CI, 2.55-6.27; P<.01 vs RR) | Rehab with transfer to rehab ward significantly reduces 5-y mortality |
| Cuccurrullo, 2019 (10) | Prospective cohort study: feasibility study followed by matched group sub-analysis  N=783 (SRP feasibility study n=136, matched NP n=66, matched SRP n=76) | Physician Visits, Outpatient Therapy, and Modified Cardiac Rehabilitation (management of blood pressure (BP), cholesterol control, reduction of blood sugar, getting active, eating better, losing weight, and smoking cessation) Lifestyle | 1-year mortality | 1.3% intervention, 15.2% control HR 0.11 (SE = 1.07, 95% CI = 0.01 - 0.90, P = 0.039) | Significant reduction in mortality with intervention |
| de Belvis, 2019 (11) | Pre-post retrospective observational study  N=1,009  Control n=483, intervention n=526 | 5-stage clinical pathway: (1) initial framing; (2) evaluation/staging; (3) treatment; (4) rehabilitation; and (5) follow-up/monitoring (full pathway) | 30-day mortality | 9.7% intervention, 8.8% control (difference 18.9%) | Mortality higher in the intervention group |
| Deutschbein, 2020 (12) | Pragmatic non-randomised controlled trial  N=361 (intervention n=145, control n=270) | Individualised case management, with regular face-to-face visits as part of a comprehensive individualised programme (PT, OT, support required), regular monitoring and adjustment (full pathway) | 1-year mortality | Intervention vs control HR 0.74 (95% CI: 0.32–1.68, P=0.57) unadjusted  Intervention vs control HR 0.65 (95% CI: 0.28–1.49, P=0.39) adjusted | No difference in mortality between groups |
| Duncan, 2020 (13) | Pragmatic cluster-randomized trial  6024 patients, 41 hospitals (Hospitals n=40, patients intervention n=2689, control n=3193) | Education, secondary prevention, rehabilitation, recovery, referrals to community-based resources, and caregiver support services by telephone follow-up | 90-day mortality | Survival to 90d, n (%): Intervention 2634 (98.0), control 3137 (98.2); OR 1.04 (0.62 to 1.75) | No difference in 90-day mortality |
| Fu, 2020 (14) | Prospective, randomised, open trial of two active and one control interventions with blinded endpoint assessment  N=400 (Primary analysis: control n=125, T1 n=123, t2 n=133) | One-to-one qualitative assessment of patient views and needs, education and goal setting | Mortality at 12 months (secondary outcome) | Death at 12 months, N/N (%): Control 2/130 (1.5), TC1 4/132 (3.0), TC2 4/138 (2.9), Odds ratio difference (95% CI) TC2 + TC1 versus control 1.95 (0.41 to 9.34), p=0.40 | No difference in 12-month mortality |
| Liu et al., 2014 (15) | Multi centre randomized controlled trial  Control n=122, intervention n=121 | Early intervention: Standard care but at 48 hours following ICH onset. | Mortality at 3 and 6 months | Mortality:  3 months: Control 10/122, intervention 1/121  6 months: Control 12/122, intervention 3/121 | Commencement of rehabilitation within 48 hours of ICH significantly reduced hospital length of stay and improved long-term survival and morbidity outcomes when compared with standard practices |
| Luengo-Fernandez et al., 2022 (EXPRESS) (16) | Prospective population-based before (phase 1: April 2002–September 2004; n=310) versus after (phase 2: October 2004–March 2007; n=281) study  EXPRESS: Phase 1 n=310, Phase 2 n=281; Control: Phase 1 n=167, Phase 2 n=224 | EXPRESS Phase 1: Clinic referral (1-2 days), brain imaging and ECG, ultrasound/echo, primary care treatment protocol comprising antiplatelet, statin, BP lowering. Phase 2: As Phase 1 but treatment initiated immediately instead of by primary care, high-dose aspirin or clopidogrel, CT scan during clinic. Follow-up 1, 6, 12, 60, and 120 months after the index event in both Phases. Control: standard care (not referred to study clinic) | Mortality | Death, n (%):  1 month: Control Phase 1 14/167 (8), Phase 1 7/310 (2), Control Phase 2 15/224 (7%), Phase 2 4/281 (1)  6 months: Control Phase 1 16/167 (10), Phase 1 14/310 (5), Control Phase 2 23/224 (10%), Phase 2 9/281 (3)  1 year: Control Phase 1 26/167 (16), Phase 1 24/310 (8), Control Phase 2 32/224 (14), Phase 2 12/281 (4)  5 years: Control Phase 1 77/167 (46), Phase 1 89/310 (29), Phase 2 control 89/224 (40), Phase 2 62/281 (22)  10 years: Control Phase 1 109/167 (65), Phase 1 152/310 (49), control Phase 2 132/224 (59), Phase 2 123/281 (44) | Urgent assessment and treatment of patients with transient ischemic attack or minor stroke resulted in a long-term reduction in recurrent strokes and improved outcomes, with little atrophy of the early benefit over time, representing good value for money even with a 10-year time horizon.  A reduction in stroke risk in phase 2 was still evident at 10 years (55/23.3% versus 82/31.6%; hazard ratio=0.68 [95% CI, 0.48–0.95]; P=0.024), as was the impact on risk of disabling or fatal stroke (17/7.7% versus 32/13.1%; hazard ratio=0.54 [0.30–0.97]; P=0.036). |
| Man, 2018 (17) | Nonrandomized comparative group study  CSC n=134 (patients n=159,000), PSC n=1047 (patients n=563,941) | Comparison of stroke care in in-hospital outcomes between primary stroke centers (PSC) and comprehensive stroke centers (CSC) (pathway comparisons) | In-hospital mortality | CSC 5.5%, PSC 4.2% (standard difference -6.13%, P<0.05), OR 1.14 (95% CI: 1.01-1.19) | Lower mortality in primary stroke centre (PSC) compared with comprehensive stroke centre (CSC) |
| Mofidi et al., 2018 (18) | Retrospective registry study  Control n=133, intervention 243 | Integrated stroke care pathway for patients with carotid artery stenosis undergoing carotid endarterectomy (CEA). Within 60 minutes: CT scan, stroke team evaluation, thrombolysis is needed; within 24 hours carotid duplex, MR angiogram, diffusion weighted MRI; within 48 hours vascular surgeon review; within 2 weeks carotid endarterectomy, all cases discussed in MDT | Mortality at 30 days | There was no significant difference in peri-operative stroke death rate between integrated (5/243, 2.1%) and standard (2/133, 1.5%) pathways (chi-square = 0.14, p = .73). | No difference in mortality rates |
| Pedapati et al., 2021 (19) | Prospective single-centre, cluster-randomised, open-label, blinded endpoint superiority trial  N=164 (Int n=82, control n=82) | Education and exercise plan tailored to the home needs of patients | In-hospital mortality, 90-day mortality | In-hospital mortality: 12.2% intervention, 19.5% control (P=0.2)  90-day mortality: 24.4% intervention, 30.5% control (p=0.38) | No significant differences in mortality between groups |
| Peng, 2017 (20) | Propensity score-matched case-control study using the National Health Insurance data  Original cohort: control n=3159, int n=1480 Matched Cohort: control n=657, int n=657 | PAC program measuring activities of daily living, nutrition, exercise, mobility and language | 90-day mortality | 1.4% intervention, 2.0% control (OR 0.68, 95% CI 0.29-1.62) | No significant differences in mortality between groups |
| Saal et al., 2015 (21) | Randomised controlled clinical trial  Control n=111, intervention n=119 | Post-discharge support including follow-up call and visit, education and training sessions | Mortality at 12 months | Mortality Intervention: 5 (3.9); Control: 15 (11.6); RR (95% CI): 0.34 (0.13; 0.90); RD (95% CI) - 0.06 (-0.13; 0.00) | The intervention group had a significantly lower mortality rate (3.9%) compared to the control group (11.6%) with a relative risk of 0.34 (95% CI, 0.13 to 0.90) and an adjusted hazard ratio of 0.32 (95% CI, 0.12 to 0.88). |
| Schwartzbach et al., 2023 (SANO) (22) | Prospective, open-label, cluster-randomised controlled trial  Control n=1283, intervention n=1203 | 1-year patient-centred integrated care intervention including regular follow-up, lifestyle, goal setting and motivational interviewing (SANO) | All-cause death at 12 months | All-cause death: Control: 31/1283 (2·4%); Intervention: 12/1203 (1·0%); OR 0·42 (95% CI 0·20–0·86); aOR 0·61 (95% CI 0·26–1·46) | This study demonstrated that the SANO programme had positive effects on the control of some cardiovascular risk factors in patients with stroke, but this did not translate into a reduction in the rate of major cardiovascular events 1 year after the first ischaemic stroke. |
| Swanson, 2019 (23) | Retrospective cohort study  Control: n-=2605, rehab only n=689, home nursing only n=535, home nursing and rehab n=898 | Standalone home nursing or rehabilitation, or a combination of both, with or without GP follow-up visits (pathway) | 90-day and 1-year mortality | Adjusted HR 90-day:  Rehab 2.69 (95% CI: 1.80–4.03)  Home nursing 0.81* (95% CI: 0.45–1.47)  Rehab + Home nursing 2.54 (95% CI: 1.70–3.81)  Adjusted HR 1-year:  Rehab 2.38 (95% CI: 1.83–3.09)  Home nursing 1.00* (95% CI: 0.71–1.41)  Rehab + Home nursing 2.20 (95% CI: 1.68–2.89)  *Significant difference to home nursing with rehabilitation, p < 0.05 | 90- and 365-day mortality rates were significantly higher in the intervention groups |
| Willeit et al., 2020 (STROKE-CARD) (24) | Pragmatic open-label two-centre randomised controlled trial with blinded outcome assessment of STROKE-CARD disease management programme and standard care  Control n=711, intervention n=1438 | STROKE-CARD care is a disease management programme by a multidisciplinary stroke team that comprises a standardised 3-month visit and access to a web-based patient portal targeting risk factor management, post-stroke complications, comorbidities and cardiovascular warning signs, rehabilitation demands, and patient education, counselling, and self-empowerment. | All-cause mortality at 12 months | All-cause mortality: Intervention 52/1438 (3.6%), control 35/711 (4.9%), HR 0.72 (95% CI 0.47, 1.10), P=0.131 | The pragmatic and easily implementable STROKE-CARD care programme reduced cardiovascular risk and improved health-related quality of life and functional outcome in patients with acute ischaemic stroke or TIA |

## Recurrent stroke

Table S2: Analysis of recurrent stroke

| **Study details** | **Design/Patients** | **Intervention** | **Outcome** | **Results** | **Comments** |
| --- | --- | --- | --- | --- | --- |
| Bath et al., 2018 (TARDIS) (2) | International (UK, Denmark, Georgia, NZ) prospective randomised open-label blinded end-point superiority clinical trial  Guideline (control) n=1540, Intervention n=1556 | Intensive treatment group given combined aspirin (load 300mg, maintenance 50-150mg daily, typically 75mg), clopidogrel (load 300mg, maintenance 75mg daily), and dipyridamole (200mg twice daily modified release, given orally; or 100mg three or four times daily). | Primary: Incidence and severity of any recurrent stroke (ischaemic, haemorrhagic; assessed using the modified Rankin Scale) or TIA within 90 days by blinded telephone follow-up. | Ordinal stroke/TIA: Intervention 93/1540 (6.0%), control 105/1530 (6.9%).  The incidence and severity of recurrent stroke or TIA did not differ between intensive and guideline therapy [adjusted common odds ratio (acOR) 0.90, 95% confidence interval (CI) 0.67 to 1.20; p = 0.47]. | The TARDIS trial found that there was no significant reduction in the recurrence of stroke or TIA, or their severity, with intensive antiplatelet therapy based on three agents as compared with guideline therapy. |
| Buckley et al., 2022 (6) | Retrospective observational study  N=836,923  Propensity matched cohorts:  Control n=2909, intervention n=2909 | Exercise-based cardiac rehabilitation | Recurrent stroke 2-year | 39.2% intervention, 42,3% control (OR 0.88, 95% CI 0.79-0.98) | 12% lower odds of recurrent stroke with intervention |
| Deutschbein, 2020 (12) | Pragmatic non-randomised controlled trial  N=361 (intervention n=145, control n=270) | Individualised case management, with regular face-to-face visits as part of a comprehensive individualised programme (PT, OT, support required), regular monitoring and adjustment (full pathway) | Recurrent stroke 1-year | Intervention vs control IRR 0.78 (95% CI: 0.39–1.57, P=0.49) unadjusted  Intervention vs control OR 0.76 (95% CI: 0.38–1.54, P=0.45) adjusted | No difference in recurrent stroke between groups |
| Ahmadi et al., 2020 (INSPiRE-TMS) (1) | Prospective, randomised, open­label, blinded endpoint, event-­driven trial  Control n=1042, intervention n=1030 | Counselling, lifestyle changes, medication adherence | 2-year recurrent stroke (secondary) | Stroke (ITT): 11.8% intervention, 11.4% control (HR 1·02, 95% CI: 0·79–1·32) | No difference in recurrent stroke between groups |
| Cheng et al., 2018 (25) | Randomised controlled trial  N=407 (Control n=200, int n=207) | Medication modification relevant to stroke risk factors, tobacco cessation, physical activity, depression, and medication adherence | 12-month recurrent stroke (secondary) | Intervention 13%, control 14%, p=0.98 | No difference in recurrent stroke between groups |
| Fu, 2020 (14) | Prospective, randomised, open trial of two active and one control interventions with blinded endpoint assessment  N=400 (Primary analysis: control n=125, T1 n=123, t2 n=133) | One-to-one qualitative assessment of patient views and needs, education and goal setting | Recurrent stroke at 12 months (secondary outcome) | Recurrent stroke by 12 months, N/N (%): control 10/130 (7.7), TC1 10/129 (7.8), TC2 4/136 (2.9), Odds ratio difference (95% CI) TC2 + TC1 versus control 0.61 (0.25 to 1.45), p=0.26 | No difference in recurrent stroke between groups |
| Luengo-Fernandez et al., 2022 (EXPRESS) (16) | Prospective population-based before (phase 1: April 2002–September 2004; n=310) versus after (phase 2: October 2004–March 2007; n=281) study  EXPRESS: Phase 1 n=310, Phase 2 n=281; Control: Phase 1 n=167, Phase 2 n=224 | EXPRESS Phase 1: Clinic referral (1-2 days), brain imaging and ECG, ultrasound/echo, primary care treatment protocol comprising antiplatelet, statin, BP lowering. Phase 2: As Phase 1 but treatment initiated immediately instead of by primary care, high-dose aspirin or clopidogrel, CT scan during clinic. Follow-up 1, 6, 12, 60, and 120 months after the index event in both Phases. Control: standard care (not referred to study clinic) | Recurrent stroke at 10 years | Recurrent stroke, n (%) 90 days: Phase 1 32/310 (10%), Phase 2 6/281 (2%)  10 years: Control Phase 1 39/167 (23%), Phase 1 82/310 (26%), Control Phase 2 55/224 (25), Phase 2 55/281 (20%) | Urgent assessment and treatment of patients with transient ischemic attack or minor stroke resulted in a long-term reduction in recurrent strokes and improved outcomes, with little atrophy of the early benefit over time, representing good value for money even with a 10-year time horizon.  A reduction in stroke risk in phase 2 was still evident at 10 years (55/23.3% versus 82/31.6%; hazard ratio=0.68 [95% CI, 0.48–0.95]; P=0.024), as was the impact on risk of disabling or fatal stroke (17/7.7% versus 32/13.1%; hazard ratio=0.54 [0.30–0.97]; P=0.036). |
| Mofidi et al., 2018 (18) | Retrospective registry study  Control n=133, intervention 243 | Integrated stroke care pathway for patients with carotid artery stenosis undergoing carotid endarterectomy (CEA). Within 60 minutes: CT scan, stroke team evaluation, thrombolysis is needed; within 24 hours carotid duplex, MR angiogram, diffusion weighted MRI; within 48 hours vascular surgeon review; within 2 weeks carotid endarterectomy, all cases discussed in MDT | Recurrent stroke at 30 days | Recurrent stroke: Intervention 5/243 (2.1%), control 2/133 (1.5%) | Recurrent stroke rates were higher in the intervention group |
| Nguyen-Huynh, 2018 (26) | Sequential pre-post intervention group study  N=867 (Control n=310, int n=557) | Telestroke program for thrombolysis through a dedicated tele-neurologist to advise on alteplase delivery (pathway) | Symptomatic ICH at 9 months | There was no significant difference in symptomatic intracranial haemorrhage rates in the 2 periods (3.8% versus 2.2% before implementation; P=0.29). | No significant difference in recurrent stroke rates |
| Reeves, 2019 (27) | Open-label (unblinded) 3-group parallel-design clinical trial  N=320 (Group 1 (UC): Global-10 n=77, PAM n=76, Group 2 (SWCM) Global-10 n=82, PAM n=79, Group 3 (SWCM + MISTT website) Global-10 n=81, PAM n=78)  UC, usual care; MISTT, Michigan stroke transitions trial; PAM, pain activation measure; SWCM, social worker–led case management | Home-based social worker–led case management (SWCM) program with or without a website, comprising biopsychosocial assessment followed by development of a personalised plan to address unmet needs (pathway, psychological) | Stroke/TIA recurrence at 90 days | 90-day stroke/TIA recurrence (n=265)  UC 7 (8%)  SWCM 5 (6%) OR 0.69 [0.21–2.26]  SWCM+MISTT Website 12 (13%) OR 1.76 [0.66–4.70]  Type 3 χ2 global test: P=0.20 | Recurrent stroke/transient ischemic attack occurred in 24 patients (9.1%), but rates were similar across groups (P=0.20). |
| Saal et al., 2015 (21) | Randomised controlled clinical trial  Control n=111, intervention n=119 | Post-discharge support including follow-up call and visit, education and training sessions | Recurrent stroke (reinfarction) at 12 months | Reinfarct Intervention: 3 (2.7); Control: 4 (3.8); RR (95% CI) 0.71 (0.16; 3.10); RD (95% CI) -0.01 (-0.09; 0.07) | No significant differences between groups in the number of recurrent strokes. |
| Schwartzbach et al., 2023 (SANO) (22) | Prospective, open-label, cluster-randomised controlled trial  Control n=1283, intervention n=1203 | 1-year patient-centred integrated care intervention including regular follow-up, lifestyle, goal setting and motivational interviewing (SANO) | Recurrent stroke at 12 months | First recurrent stroke: Control: 50/1283 (3·9%); Intervention: 51/1203 (4·2%); OR 1·00 (95% CI 0·58–1·73); aOR 1·03 (95% CI0·53–1·99) | This study demonstrated that the SANO programme had positive effects on the control of some cardiovascular risk factors in patients with stroke, but this did not translate into a reduction in the rate of major cardiovascular events 1 year after the first ischaemic stroke. |
| Sharma et al., 2019 (COMPASS) (28) | COMPASS was a randomized, double-blind, double-dummy trial  Prior stroke: rivaroxaban + aspirin n=351, rivaroxaban alone n=346, aspirin alone n=335. Total n=1032 | Participants had stable coronary artery or peripheral artery disease and were randomly assigned to receive aspirin 100 mg once daily (n=9126), rivaroxaban 5 mg twice daily (n=9117), or rivaroxaban 2.5 mg twice daily plus aspirin (n=9152). | Recurrent stroke at 23 months | Recurrent stroke: R+A (n=351) 9/351, R (n=346) 18/346, A (n=335) 20/335  R+A vs A HR 0.42 (0.19-0.92) p=0.03  R vs A: HR 0.88 (0.47-1.67) p=0.70 | In summary, low-dose rivaroxaban plus aspirin is an important new option for efficacious antithrombotic therapy for primary and especially secondary prevention of stroke in patients with atherosclerosis. The absolute risk reduction for secondary prevention is substantial and makes a compelling case favoring the use of 2.5 mg rivaroxaban twice daily plus aspirin in these patients. |
| Willeit et al., 2020 (STROKE-CARD) (24) | Pragmatic open-label two-centre randomised controlled trial with blinded outcome assessment of STROKE-CARD disease management programme and standard care  Control n=711, intervention n=1438 | STROKE-CARD care is a disease management programme by a multidisciplinary stroke team that comprises a standardised 3-month visit and access to a web-based patient portal targeting risk factor management, post-stroke complications, comorbidities and cardiovascular warning signs, rehabilitation demands, and patient education, counselling, and self-empowerment. | Recurrent stroke at 12 months | Stroke or TIA: Intervention 91/1438 (6.3%), control 46/711 (6.5%), HR 0.96 (95% CI 0.67, 1.37), P=0.822 | The pragmatic and easily implementable STROKE-CARD care programme reduced cardiovascular risk and improved health-related quality of life and functional outcome in patients with acute ischaemic stroke or TIA |
| Yan et al., 2021 (29) | Open-label, two-arm cluster-randomised controlled trial  villages n=50, participants n=1299  Control n=615, int n=611 | Primary care based app (SINEMA) to improve communication between HCPs and patients (mhealth) | 12-month recurrent stroke (exploratory) | 4.4% intervention versus 9.3% control; (risk ratio [RR] = 0.46, 95% CI 0.32, 0.66; risk difference [RD] = 4.9 percentage  points [pp]), p<0.0001 | Significant reduction in recurrent stroke rate with intervention |

## Major bleeding

Table S3: Analysis of major bleeding

| **Study details** | **Design/Patients** | **Intervention** | **Outcome** | **Results** | **Comments** |
| --- | --- | --- | --- | --- | --- |
| Ahmadi et al., 2020 (INSPiRE-TMS) (1) | Prospective, randomised, open­label, blinded endpoint, event-­driven trial  Control n=1042, intervention n=1030 | Screening for complications, QoL, depression | Major bleeding (2-year) secondary | Major bleeding (ITT) events/1000 patient years (possibly more than 1 event per patient): Intervention 32 (9; 6–12), control 38 (10; 7–14) IRR 0·84 (0·53–1·35) | No significant difference in major bleeding between groups |
| Bath et al., 2018 (TARDIS) (2) | International (UK, Denmark, Georgia, NZ) prospective randomised open-label blinded end-point superiority clinical trial  Guideline (control) n=1540, Intervention n=1556 | Intensive treatment group given combined aspirin (load 300mg, maintenance 50-150mg daily, typically 75mg), clopidogrel (load 300mg, maintenance 75mg daily), and dipyridamole (200mg twice daily modified release, given orally; or 100mg three or four times daily). | Secondary: bleeding and its severity | Major bleeding events: Intervention 31/1541 (2.0%), control 14/1531 (0.9%)  Major bleeding (NIHSS score)  Score >3:  Intervention 442/1540 (28.7%), control 435/1530 (28.4%)  Score ≤3 or TIA:  Intervention 1099/1540 (71.3%), control 1096/1530 (71.6%) | Significantly more bleeding events overall in TARDIS group (19.8%) compared with control (9.1%, p<0.001), prompting the study to be stopped early. |
| Mofidi et al., 2018 (18) | Retrospective registry study  Control n=133, intervention 243 | Integrated stroke care pathway for patients with carotid artery stenosis undergoing carotid endarterectomy (CEA). Within 60 minutes: CT scan, stroke team evaluation, thrombolysis is needed; within 24 hours carotid duplex, MR angiogram, diffusion weighted MRI; within 48 hours vascular surgeon review; within 2 weeks carotid endarterectomy, all cases discussed in MDT | Major bleeding at 30 days | Major bleeding: Intervention 6/243 (2.5%), control 3/133 (2.3%) | More patients in the intervention group were returned to theatre as a result of major bleeding |
| Schwartzbach et al., 2023 (SANO) (22) | Prospective, open-label, cluster-randomised controlled trial  Control n=1283, intervention n=1203 | 1-year patient-centred integrated care intervention including regular follow-up, lifestyle, goal setting and motivational interviewing (SANO) | Major bleeding events (SAEs) at 12 months | Bleeding Control 8/1152 (0·7%), Intervention 31/1151 (2·6%) | This study demonstrated that the SANO programme had positive effects on the control of some cardiovascular risk factors in patients with stroke, but this did not translate into a reduction in the rate of major cardiovascular events 1 year after the first ischaemic stroke. |
| Sharma et al., 2019 (COMPASS) (28) | COMPASS was a randomized, double-blind, double-dummy trial  Prior stroke: rivaroxaban + aspirin n=351, rivaroxaban alone n=346, aspirin alone n=335. Total n=1032 | Participants had stable coronary artery or peripheral artery disease and were randomly assigned to receive aspirin 100 mg once daily (n=9126), rivaroxaban 5 mg twice daily (n=9117), or rivaroxaban 2.5 mg twice daily plus aspirin (n=9152). | Major bleeding events at 23 months | Major bleeding:  R+A 12/351, R 26/346, A 19/335  R+A vs A HR 3.79 (1.07-13.4), p=0.04  R vs A: HR 3.84 (1.08-13.6), p=0.04 | In summary, low-dose rivaroxaban plus aspirin is an important new option for efficacious antithrombotic therapy for primary and especially secondary prevention of stroke in patients with atherosclerosis. The absolute risk reduction for secondary prevention is substantial and makes a compelling case favoring the use of 2.5 mg rivaroxaban twice daily plus aspirin in these patients. |
| Willeit et al., 2020 (STROKE-CARD) (24) | Pragmatic open-label two-centre randomised controlled trial with blinded outcome assessment of STROKE-CARD disease management programme and standard care  Control n=711, intervention n=1438 | STROKE-CARD care is a disease management programme by a multidisciplinary stroke team that comprises a standardised 3-month visit and access to a web-based patient portal targeting risk factor management, post-stroke complications, comorbidities and cardiovascular warning signs, rehabilitation demands, and patient education, counselling, and self-empowerment. | Major bleeding events at 12 months | Major bleeding: Intervention 20/1438 (1.4%), control 15/711 (2.1), HR 0.65 (0.33, 1.27), P=0.210 | The pragmatic and easily implementable STROKE-CARD care programme reduced cardiovascular risk and improved health-related quality of life and functional outcome in patients with acute ischaemic stroke or TIA |

# Secondary outcomes

## Quality of life

Subgroup meta-analysis showed that the greatest differences in QoL were observed with SF-36 (Figure S1) and SSQOL (Figure S2).


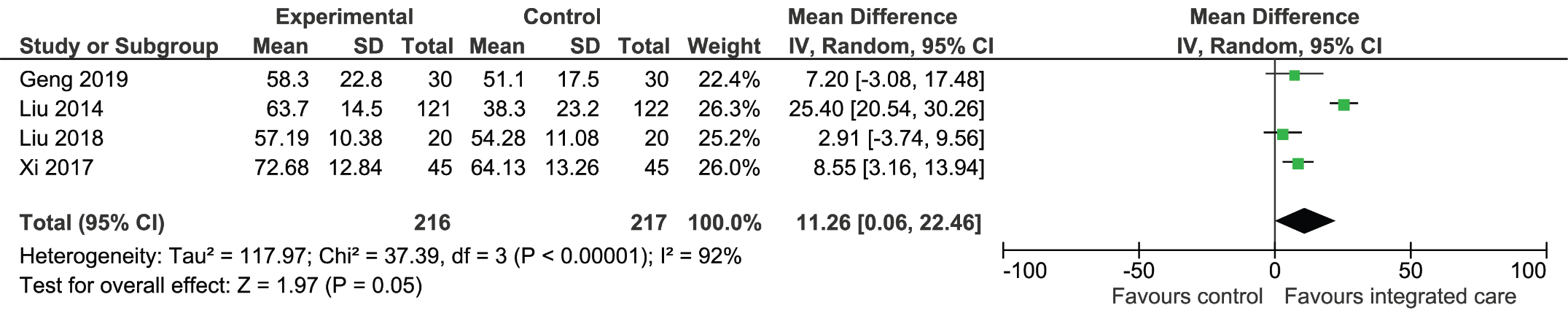


**Figure S1: Comparison of integrated care with Control for QoL (SF-36 general health scores)**


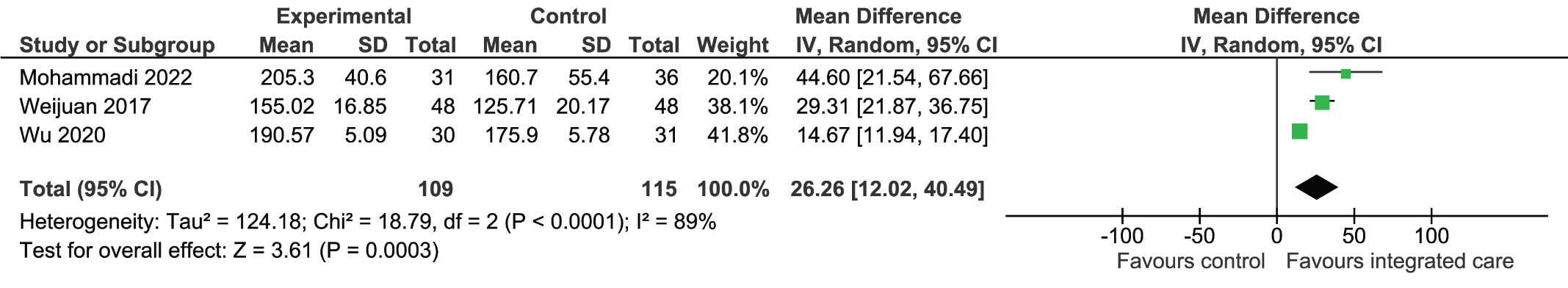


**Figure S2: Comparison of integrated care with Control for QoL (SSQOL scores)**

Table S4: Analysis of quality of life

| **Study details** | **Design/Patients** | **Intervention** | **Outcome** | **Results** | **Comments** |
| --- | --- | --- | --- | --- | --- |
| Abdul Aziz, 2020 (30) | Pragmatic cluster randomised controlled trial-within trial at public primary care health centres  Control n=65, iCaPPS n=86 | Screening for complications, QoL, depression | QoL (primary) EQ-5D-5L at 6 months  Cost/QALY (secondary) | 6-month EQ-5D-5L score changes:  Improvement: Intervention 36.1%, control 23.1%  No change: Intervention 39.5%, control 69.2%  Deterioration: Intervention 24.4%, control 7.7%  Cost/QALY  Intervention USD469.60 Control USD538.41 | No significant differences in EQ-5D-5L scores at baseline and at 6 months (p > 0.05) More cost-effective (12.7% lower) than standard care (cost/QALY) |
| Bath et al., 2018 (TARDIS) (2) | International (UK, Denmark, Georgia, NZ) prospective randomised open-label blinded end-point superiority clinical trial  Guideline (control) n=1540, Intervention n=1556 | Intensive treatment group given combined aspirin (load 300mg, maintenance 50-150mg daily, typically 75mg), clopidogrel (load 300mg, maintenance 75mg daily), and dipyridamole (200mg twice daily modified release, given orally; or 100mg three or four times daily). | Secondary: QoL | EQ-5D-3L score, mean (SD) at 90 days:  Intervention 0.7 (0.3), control 0.7 (0.3) | No difference in quality of life between groups |
| Bragstad et al., 2020 (31) | Multicenter, prospective, randomized, assessor-blinded, controlled trial with two parallel groups  Gave consent n=353, randomised n=322  Control n=156, intervention n=166 | Dialogue-based intervention to promote psychosocial wellbeing (psychological) | Stroke and Aphasia Quality of Life Scale (SAQOL) at 12 months | Between-group differences at 12 months (mean, SE):  Physical domain 0.06 (−0.07, 0.20), P=0.36  Communication domain 0.06 (−0.05, 0.16), P=0.27  Psychosocial domain 0.07 (−0.11, 0.25), P=0.45 | No difference in QoL at 12 months |
| Brouns, 2021 (32) | Pre-post test, controlled, pragmatic clinical trial  Control n=258, intervention n=310 | Web-based e-rehabilitation comprising physical exercise and cognitive exercise (psychological) programmes (mhealth) | QoL EQ-5D-5L at 3 and 6 months | Within group EQ-5D-5L changes (mean, SD):  3 months Intervention 0.07 (0.2), control 0.07 (0.2), between group P=0.19  6 months Intervention 0.01 (0.2), control –0.01 (0.2), between group P=0.38  LMM (0-6 months) P-value 0.50 (95% CI: 0.0–0.1) | No difference in QoL at 3 or 6 months |
| Brouwer et al., 2018 (33) | Multicenter randomized controlled trial with 2 groups: an intervention (“tune-up”) group and a control group having the same exposure to assessment  N=132 (Control n=52, Intervention n=51) | Patient-centred treatment sessions comprising strength/power, balance, cardiovascular endurance (exercise), motor coordination, and education about relevant community resources, as well as how to monitor their mobility and activities | QoL (SF-36) at 12 months | SF-36 difference at 12 months  Physical component -1.7 (-4.9 - 1.5), p=0.30  Mental component -1.6 (-5.4 - 2.1), p=0.40 | No difference in QoL at 12 months |
| Chiu, 2021 (34) | Large-scale prospective cohort study  N=1786 (PAC n=273, non-PAC n=273) | Post-acute care (PAC): physical exercise (physical, occupational, or speech-swallowing therapy) in multiple sessions | QoL EQ-5D at 12 months | Mean (SE) differences (PAC vs non-PAC) at 1 year after rehabilitation:  -2.03 (-0.26) | No difference in QoL at 12 months |
| Chu, 2020 (35) | Single-centre randomised controlled trial  N=61 (Control n=30, intervention n=31) | Training for caregivers (education), personalised training plan for patients for recovery of physical function (mobility, self-care, bowel/toilet control) | QoL EQ-5D at 6 months | Comparison of EQ-5D at 6 months post-hospital  Intervention (n=26): EQ-5D 1.55 (0.39),  Control (n=30): EQ-5D 1.56 (0.54),  t: EQ-5D 0.11  P: EQ-5D 0.9125 | No difference in QoL at 6 months |
| Cumming, 2019 (36) | International, multicenter (56 sites), pragmatic single-blind phase 3 randomized controlled trial, spanning 2006–2015  N=2104 (Intervention n=1054, control n=1050) | Very early mobilisation (physical activity within 24 hours) exercise | QoL AQoL 4D at 12 months | Adjusted median regression indicated no signiﬁcant group diﬀerence in either total AQoL-4D scores (coeﬃcient = −0.004; 95% conﬁdence interval [CI] −0.043, 0.036; p = 0.86) or in any of the 4 domains at 12 months: Independent Living (coeﬃcient = −0.007; 95% CI −0.038, 0.024; p = 0.66), Social Relationships (coeﬃcient = 0.011; 95% CI −0.007, 0.030; p = 0.24), Physical Senses (coeﬃcient = 0.009; 95% CI −0.004, 0.023; p = 0.16), and Psychological Well-being (coeﬃcient = 0.001; 95% CI −0.011, 0.014; p = 0.85) | No significant difference in QoL at 12 months |
| Døhl, 2020 (37) | Multicentre, pragmatic, single-blinded, randomized controlled trial  N=380 (Intervention n=186, control n=194) | A follow-up program comprising monthly individualized coaching by a physiotherapist, physical activity and exercise schedule, training diary | QoL EQ-5D-5L at 18 months | Regression results* of individual cost coefficient measured as proportion increase in cost with one unit increase, with 95% CI. EQ-5D-5L as Need variable:  Total costs: −3.50† (− 4.26 − − 2.75)  Primary care: −3.48† (− 5.22 − − 1.74)  Hospital care: −2.19† (− 3.14- -1.25)  GP: −2.15 (− 3.05 − − 1.25)  * Results from the regression †P < 0.05 | No reduction in healthcare costs and no difference in QoL |
| Fu, 2020 (14) | Prospective, randomised, open trial of two active and one control interventions with blinded endpoint assessment  N=400 (Primary analysis: control n=125, T1 n=123, t2 n=133) | One-to-one qualitative assessment of patient views and needs, education and goal setting | QoL SF-36 PCS at 12 months | Primary outcome  SF-36 PCS 12 months, mean (SD): control 43.4 (10.7) n=125, TC1 45.4 (8.4) n=123, TC2 47.3 (8.4) n=133; difference (95% CI) TC2+TC1 minus control 2.9 (0.95 to 4.9), p=0.004  Secondary outcomes  SF-36 PCS adjusted* 12 months: TC2+TC1 minus control 1.8 (0.15 to 3.5), p=0.032  SF-12 PCS six months mean (SD): control 41.9 (10.1) n=113, TC1 43.5 (8.9) n=113, TC2 45.1 (7.5) n=125; difference (95% CI) TC2+TC1 minus control 2.4 (0.4 to 4.4), p=0.018  * Adjusted for Barthel Index five days after stroke, SF-12 PCS at baseline, age, sex, whether living alone | Significantly improved QoL with intervention |
| Geng, 2019 (38) | Randomised controlled trial  N=101 (Control n=30, intervention n=30) | Physical function, emotional function, lifestyle, medication adherence | QoL SF-36 at 6 months | QoL domain scores (mean [SD])  Physical functioning: Intervention 81.4 (16.7), control 70.5 (25.9); P<0.001  Role limitations due to physical problem: Intervention 85.3 (21.7), control 72.4 (23.5), P=0.004  Bodily pain: Intervention 78.1 (16.0), control 82.6 (16.4), P=0.835  General health: Intervention 58.3 (22.8), control 51.1 (17.5), P<0.001  Vitality: Intervention 77.2 (13.3), control 70.1 (13.1), P<0.001  Social functioning: Intervention 79.5 (21.9), control 67.1 (19.3), P=0.003  Role limitations due to emotional problems: Intervention 79.4 (22.6), control 54.0 (37.3), P=0.198  Mental health: Intervention 78.3 (17.3), control 69.2 (18.2), P<0.001 | Significant differences in QoL domains in favour of intervention except for bodily pain and role limitations due to emotional problems |
| He et al., 2023 (39) | Randomized, historically controlled clinical trial  Control n=124, intervention n=161 | Rapid recovery implementation process to optimise patient recovery post-surgery | QoL (EQ-5D) | EQ-5D-5 L:  Mobility 1.69 (0.78) (control),1.6 (0.82) (intervention), p=0.349  Self-care 1.56 (0.78) (control), 1.65 (0.82) (intervention), p=0.394  Usual activities 1.6 (0.76) (control), 1.61 (0.78) (intervention), p=0.897  Pain/discomfort 1.27 (0.48) (control),1.33 (0.58) (intervention), p=0.383  Anxiety/depression 1.39 (0.56) (control), 1.23 (0.43) (intervention), p=0.013  Visual analog scale 64.72 (25.93) (control), 72.29 (26.94) (intervention), p=0.017 | Significant differences were found in anxiety/depression and overall quality of life, with the intervention group showing better outcomes. |
| Hjelle, 2019 (40) | Multicentre, prospective, randomized controlled trial  Enrolled n=353, randomised n=322 (Control n=156, inv n=166) | Dialogue-based intervention delivered in patient home by nurse/OT (psychological) | SAQOL at 6 months | SAQOL between group differences (95% CI)  Physical domain: –0.028 (–0.14 to 0.09), P=0.645  Communication domain: –0.031 (–0.10 to 0.04), P=0.437  Psychosocial domain: –0.004 (–0.21 to 0.20), P=0.967  Overall: –0.026 (–0.13 to 0.08), P=0.637 | No significant between group differences in QoL |
| Jones et al., 2016 (41) | Cluster randomised controlled trial  Control n=30, intervention n=36 | Bridges self-management program (SMP) comprising one-to-one rehabilitation sessions and stroke workbook | QoL (SAQOL) at 12 weeks | SAQOL mean scores:  Baseline: control 3.2±0.8, intervention 3.4±0.8  6 weeks: control 3.1±1.0, intervention 3.3±0.8  12 weeks: control 3.1±0.8, intervention 3.4±0.8 | No significant differences in QoL |
| Kalav, 2022 (42) | Single centre, single-blinded randomised controlled experimental study  N=210 (Control n=34, int n=34) | Individualised patient education with follow-up | SSQOL at 12 weeks | Changes in SSQOL domains, mean ±SD:  Activities: control 0.62 ± 0.88, intervention 0.62 ± 0.88 (t=-0.82, p=0.4136)  Social and family roles: control 0.60 ± 1.25, intervention 0.79 ± 1.18 (t= -0.77, p=0.4446)  Language: Control 0.46 ± 1.04, intervention 0.92 ± 1.20 (t=-1.69, p=0.0959)  Vision: control 0.36 ± 0.72, intervention 0.37 ± 1.00 (t=-0.05, p=0.9632)  Energy: control 0.51 ± 1.14, intervention 0.54 ± 0.95 (t=-0.27, p=0.7861)  Mood: control 0.01 ± 1.18, intervention -0.26 ± 1.40 (t= 0.76, p=0.4507)  Personality: control 0.35 ± 1.02, intervention 0.09 ± 1.24 (t= 0.78, p=0.4375)  SSQoL: control 0.44 ± 0.67, intervention 0.54 ± 0.79 (t=-0.71, p=0.4803) | No significant difference in QoL |
| Kam Yuet Wong et al., 2022 (43) | Randomised controlled trial  N=2612 (Control n=58, int n=58) | Holistic MDT-based exercise training programme with psychological and social support | QOL: EQ-5D-5L at 3 and 6 months | GEE (mean, SE) EQ-5D-5L at 6 months:  Baseline: Int 0.66 (0.01), control 0.66 (0.02); p=0.768  Post-intervention: Int 0.83 (0.02), control 0.77 (0.02); p=0.026 | Significant improvements in QoL with intervention |
| Khramov, 2021 (44) | Prospective randomized controlled parallel-group study  N=122 (Control n=59, int n=63) | Exercise, massage, PT and OT, social adaptation, goal setting | Stroke-specific QoL (SSQOL) at 6 months | SSQOL domain scores, M ± m  Energy: Control 2.60 ± 0.17, Intervention 3.07 ± 0.15*  Family roles: Control 2.43 ± 0.21, Intervention 3.72 ± 0.32*  Language: Control 3.81 ± 0.16, Intervention 3.74 ± 0.26  Mobility: Control 2.92 ± 0.37, Intervention 4.31 ± 0.29*  Mood: Control 2.85 ± 0.28, Intervention 4.34 ± 0.41*  Personality: Control 3.13 ± 0.32, Intervention 3.32 ± 0.22  Self-care: Control 3.82 ± 0.20, Intervention 4.70 ± 0.19*  Social role: Control 2.74 ± 0.17, Intervention 3.82 ± 0.25*  Thinking: Control 2.51 ± 0.24, Intervention 2.61 ± 0.16  Upper extremity function: Control 4.03 ± 0.19, Intervention 4.74 ± 0.21*  Vision: Control 2.72 ± 0.21, Intervention 2.92 ± 0.15  Work, productivity: Control 2.41 ± 0.15, Intervention 3.20 ± 0.28*  * — differences are significant (р < 0.05) relative to the comparison group ( the Mann–Whitney U test). | More pronounced improvement in SS-QOL (p < 0.05) scores for intervention group |
| Langhorne et al., 2017 (45) | Pragmatic, prospective, parallel-group, multicentre, international, RCT with blinded assessment of outcomes and an intention to treat analysis  N=2104 (Control n=1050, int n=1054) | Very early mobilisation (within 24h of stroke onset) Exercise | QoL (AQOL) at 12 months | The median AQoL IQR for the VEM group was 0.47 (95% CI 0.07 to 0.81) and in the UC group was 0.49 (95% CI 0.08 to 0.81; p = 0.865). The adjusted median regression result was –0.0036 (95% CI –0.045 to 0.038; p = 0.865). | No significant between group differences in QoL |
| Lewthwaite, 2018 (46) | Parallel group, three-arm, single blind, superiority randomized controlled trial of a theoretically-defensible, upper extremity rehabilitation program (47)  N=361 (UCC n=122, DEUCC n=120, ASAP n=119) | Accelerated Skill Acquisition Program (ASAP) comprising skill acquisition through task-specific practice, impairment mitigation to increase capacity, and motivational enhancements to build self- confidence (exercise, psychological) | QoL (EQ-5D visual analogue scale [VAS]) at 3 months | End of treatment EQ-5D scores:  Intervention (ASAP): 77.1 (73.6, 80.5)*  Dose-equivalent usual care (DEUCC): 71.9 (68.6, 75.1)  Usual care (UCC): 70.7 (66.8, 74.5)  *P<0.05 vs DEUCC, P<0.001 vs UCC | Significant improvements in QoL at 3 months in intervention versus comparator groups |
| Liu, 2018 (48) | Randomised trial  N=40 (control n=20, int n=20) | Nurse-led personalised education and follow-up | QoL (SF-36) at 1, 3 and 6 months | At month 1, 6/8 domains of SF-36 were significantly improved over control (P<0.05) excluding vitality (VT) and social functioning (SF)  At month 3, 4/8 domains showed significance excluding bodily pain (BP), VT, SF and role limitations due to emotional problems (RE)  At month 6, 1/8 domains (General health perceptions [GH]) were significant | QoL significantly improved versus control at 1 month but effect declined with continuing follow-up |
| Liu et al., 2014 (15) | Multi centre randomized controlled trial  Control n=122, intervention n=121 | Early intervention: Standard care but at 48 hours following ICH onset. | HRQoL (SF-36) | SF-36 Mean differences between components at 3 months varied were -2.5 to 5.8 between groups, 6.4-25.5 at 6 months | Commencement of rehabilitation within 48 hours of ICH significantly reduced hospital length of stay and improved long-term survival and morbidity outcomes when compared with standard practices |
| Luengo-Fernandez et al., 2022 (EXPRESS) (16) | Prospective population-based before (phase 1: April 2002–September 2004; n=310) versus after (phase 2: October 2004–March 2007; n=281) study  EXPRESS: Phase 1 n=310, Phase 2 n=281; Control: Phase 1 n=167, Phase 2 n=224 | EXPRESS Phase 1: Clinic referral (1-2 days), brain imaging and ECG, ultrasound/echo, primary care treatment protocol comprising antiplatelet, statin, BP lowering. Phase 2: As Phase 1 but treatment initiated immediately instead of by primary care, high-dose aspirin or clopidogrel, CT scan during clinic. Follow-up 1, 6, 12, 60, and 120 months after the index event in both Phases. Control: standard care (not referred to study clinic) | Quality of life (EQ-5D) | EQ-5D scores by time period, mean (SD):  1 month: Phase1 0.751 (0.268), Control Phase 1 0.689 (0.279), Phase 2 0.784 (0.227). Control Phase 2 0.750 (0.249)  1 year: Phase 1 0.759 (0.245), Control Phase 1 0.736 (0.247), Phase 2 0.765 (0.264), Control Phase 2 0.721 (0.271)  5 years: Phase 1 0.759 (0.262), Control Phase 1 0.754 (0.239), Phase 2 0.757 (0.269), Control Phase 2 0.719 (0.283)  10 years: Phase 1 0.705 (0.325), Control Phase 1 0.766 (0.307), Phase 2 0.718 (0.320), Control Phase 2 0.746 (0.271) | Urgent assessment and treatment of patients with transient ischemic attack or minor stroke resulted in a long-term reduction in recurrent strokes and improved outcomes, with little atrophy of the early benefit over time, representing good value for money even with a 10-year time horizon.  A reduction in stroke risk in phase 2 was still evident at 10 years (55/23.3% versus 82/31.6%; hazard ratio=0.68 [95% CI, 0.48–0.95]; P=0.024), as was the impact on risk of disabling or fatal stroke (17/7.7% versus 32/13.1%; hazard ratio=0.54 [0.30–0.97]; P=0.036). |
| Markle-Reid, 2020 (49) | Prospective one-group pre-test/post-test pragmatic study  N=45 (participated n=30) | Multidisciplinary transitional care intervention delivering training and support to older stroke patients at home with regular consultation and follow-up (pathway) | HRQoL (SF-12) at 6 months | Difference in mean QoL scores (95% CI):  HRQoL–Physical composite summary score (SF-12) 2.3 ( 2.2, 6.8), P=0.3  HRQoL–Mental composite summary score (SF-12) 0.3 ( 3.9, 4.4), P=0.9 | No significant between group differences in QoL |
| Minshall, 2020 (50) | Randomised controlled trial  N=458 (Control: stroke patients n=39, carers n=40. 12-month assessment patients n=25, carers n=23  Int: stroke patients n=50, carers n=44. 12-month assessment patients n=27, carers n=18) | Program of personalized psychosocial support – Stroke Care Optimal Health Program (SCOHP) comprising education, self-management and reflective exercises | Australian Assessment of Quality of Life-6 Dimensions (AQoL- 6D)33 and the 3-level EuroQoL-5-dimensions (EQ- 5D-3 L) at 3, 6 and 12 months | Quality of life (AQOL), mean (SD)  Stroke survivor  Baseline: Int 0.61 (0.20), control 0.63 (0.24); p=0.71  3 months: Int 0.59 (0.18), control 0.62 (0.27); p=0.63  6 months: Int 0.58 (0.18), control 0.63 (0.27); p=0.40  12 months: Int 0.58 (0.19), control 0.64 (0.27); p=0.32  Stroke carer:  Baseline: Int 0.71 (0.19), control 0.72 (0.21); p=0.96  3 months: Int 0.77 (0.13), control 0.71 (0.19); p=0.25  6 months: Int 0.70 (0.16), control 0.72 (0.22); p=0.74  12 months: Int 0.72 (0.18), control 0.69 (0.24); p=0.69  Quality of life (EQ-5D)  Stroke survivor  Baseline: Int 65.05 (18.01), control 58.72 (23.19); p=0.21  3 months: Int 68.67 (20.34), control 65.45 (23.01); p=0.60  6 months: Int 64.03 (21.73), control 67.08 (18.24); p=0.58  12 months: Int 62.55 (20.50), control 67.00 (22.62); p=0.46  Stroke carer:  Baseline: Int 73.88 (17.49), control 74.93 (17.00); p=0.81  3 months: Int 79.22 (13.19), control 71.29 (15.89); p=0.11  6 months: Int 70.13 (19.13), control 74.60 (19.02); p=0.45  12 months: Int 72.94 (19.94), control 69.83 (19.78); p=0.64 | No significant differences between groups for QoL |
| Mohammadi, 2022 (51) | Single-blind RCT  N=80 (Control n=36, int n=31) | Personalised care plan providing education, diet, activity, anxiety (psychological) level, medication with follow-up | QoL (SSQOL) at 3 and 6 months | QoL scores at 3 and 6 months (Mean ± SD):  3 months: Intervention 172.2 ± 40.7, control 144.9 ± 51.7, P<0.05  6 months Intervention 205.3 ± 40.6, control 160.7 ± 55.4, P<0.05 | Significant improvements in QoL at 3 and 6 months in intervention versus control group |
| Reeves, 2019 (27) | Open-label (unblinded) 3-group parallel-design clinical trial  N=320 (Group 1 (UC): Global-10 n=77, PAM n=76, Group 2 (SWCM) Global-10 n=82, PAM n=79, Group 3 (SWCM + MISTT website) Global-10 n=81, PAM n=78)  UC, usual care; MISTT, Michigan stroke transitions trial; PAM, pain activation measure; SWCM, social worker–led case management | Home-based social worker–led case management (SWCM) program with or without a website, comprising biopsychosocial assessment followed by development of a personalised plan to address unmet needs (pathway, psychological) | PROMIS-10 global physical and mental health at 7 and 90 days | PROMIS-10 global physical health (n=434 observations) – difference in differences (D-in-D) analysis; test of group × time interaction: P=0.003  PROMIS-10 global mental health (n=434 observations) D-in-D analysis; test of group × time interaction: P=0.56 | Significant difference in physical health but not mental health |
| Rodgers, 2019; Shaw, 2020 (52, 53) | Parallel-group observer-blind multicenter individually randomized controlled trial  N=573 (Control n=231, int n=219) | Early supported discharge reviews (EXTRAS) including mobility, self-care, mood (psychological), cognitive function (pathway) | QALYs (EQ-5D-5L) at 1 and 2 years | Adjusted Difference* EXTRAS Minus Usual Care Mean (95% CI)  Utility year 1: 0.05 (0.00 to 0.09)  Utility year 2: 0.04 (0.01 to 0.07)  Total as QALY: 0.07 (0.01 to 0.12) | EXTRAS provided more Quality Adjusted Life Years (0.07 [95% CI, 0.01 to 0.12]) but no difference in QoL |
| Saal et al., 2015 (21) | Randomised controlled clinical trial  Control n=111, intervention n=119 | Post-discharge support including follow-up call and visit, education and training sessions | HRQoL BREF at 12 months | WHOQOL-BREF  Physical: Intervention: Baseline - 68.5 (17.4); 12 months - 65.8 (18.5); Control: Baseline - 64.4 (20.0); 12 months 63.0 (21.8) Mean difference (95% CI) -0.7 (-4.7; 3.3)  Psychological: Intervention: Baseline - 73.4 (14.9); 12 months 72.2 (17.8); Control: Baseline - 73.1 (16.8); 12 months -72.3 (18.9) Mean difference (95% CI) -1.5 (-5.4; 2.3)  Social: Intervention: Baseline - 73.7 (15.0); 12 month - 73.3 (19.0); Control: Baseline - 75.2 (15.0); 12 months 75.8 (17.3) Mean difference (95% CI) -1.8 (-6.7; 3.1)  Environment: Intervention: Baseline - 77.6 (12.0); 12 month - 78.9 (14.7); Control: Baseline- 81.3 (12.4); 12 month - 81.1 (14.5) Mean difference (95% CI) -0.7 (-4.4; 3.2)  Global: Intervention: Baseline - 61.8 (17.5);l 12 month - 59.6 (20.9); Control: Baseline - 61.7 (19.6); 12 month - 60.9 (25.3) Mean difference (95% CI) -1.8 (-7.1; 3.6) | No statistically significant differences were found between groups on HRQOL. |
| Tung et al., 2021 (54) | Retrospective cohort study  N=261 (Inpatient PAC: n=138, Home-based PAC n=59) | Post-acute care (PAC) delivering physical, OT and speech therapy in inpatient and home settings (pathway) | EuroQoL EQ-5D (ED5Q) Cost-effectiveness was calculated as the total cost divided by the improvement in Barthel index (BI), Lawton–Brody instrumental activities of daily life scale (IADL), ED5Q and mini nutritional assessment (MNA) scores | Functional improvement in QOL between inpatient PAC and home-based PAC: p=0.769  Cost-effectiveness vs. ED5Q (USD): Inpatient PAC 25,476.70 ± 52,741.59 (849.22 ± 1758.05)  Home-based PAC 15,623.60 ± 18,564.44 (520.79 ± 618.81); p<0.001 | No significant difference between groups in QoL but home-based PAC was more cost-effective |
| Vluggen, 2021 (55) | Two-arm multicenter randomised controlled trial  N=190 (Control n=91, int n=99) | Inpatient neurorehabilitation treatment, home-based self-management training (patient and caregiver), education | QoL (SSQOL) at 6 months | SSQOL domains  Subscale physical functioning (Mean [SD]):  Intervention 102.2 (19.9), control 99.2 (22.5), group effect 3.08, P=0.476  Subscale psychosocial functioning (Mean [SD]): Intervention 81.3 (17.1), control 78.7 (18.0), group effect 8.45, P=0.054 | No significant difference between groups in QoL |
| Long Weijan and Zhang, 2017 (56) | Randomised controlled trial  N=96 (Control n=48, int n=48) | Training program for HCPs on comprehensive care for patients, rehabilitation training for caregivers (pathway) | QoL (SS-QOL) at 3 months posy-discharge | SS-QOL components at 3 months, mean (SD), t-value, p=value  physical fitness: Control 6.08±0.901, Intervention 6.92±0.991; t= -4.336, P=0.000  Family role: Control 6.48±0.80, intervention 7.33±1.121; t= -4.309, P=0.000  Language features: Control 14.08±3.231, intervention 18.29±2.141; t= -7.525, P=0.000  Activity ability: Control 13.19±4.481, intervention 16.65±4.911; t= 3.605, P=0.001  Mood: Control 12.23±5.91, intervention 16.19±6.231; t= -3.193, P=0.002  Personality: Control 9.60±4.24, intervention 12.56±3.431; t= -3.759, P=0.000  Self-care ability: Control 13.71±5.69, intervention 17.91±5.691; t= -3.622, P=0.000  Social role: Control 10.33±5.31, intervention 14.23±7.181; t= -3.023, P=0.003  Thinking: Control 6.69±3.861, intervention 6.85±3.761; t=-0.214, P=0.831  Upper limb function: Control 14.04±6.07, intervention 18.23±6.601; t= -3.235, P=0.002  Vision: Control 10.23±2.75, intervention 10.52±2.581; t= -0.536, P=0.593  Work productivity: Control 9.04±1.66, intervention 9.33±1.621; t= -0.872, P=0.386  Total SSQOL score: Control 125.71±20.171, intervention 155.02±16.851; t= -7.727, P=0.000 | QoL was significantly higher in the intervention group overall at 3 months (P<0.01) and for all domains except thinking, vision and work productivity |
| Willeit et al., 2020 (STROKE-CARD) (24) | Pragmatic open-label two-centre randomised controlled trial with blinded outcome assessment of STROKE-CARD disease management programme and standard care  Control n=711, intervention n=1438 | STROKE-CARD care is a disease management programme by a multidisciplinary stroke team that comprises a standardised 3-month visit and access to a web-based patient portal targeting risk factor management, post-stroke complications, comorbidities and cardiovascular warning signs, rehabilitation demands, and patient education, counselling, and self-empowerment. | Stroke, MI, vascular death, QoL (EQ-5D-3L), functional status, major bleeding | The median EQ-5D-3L health utility score was 0.783 (IQR 0.687-1.000) in the STROKE-CARD care group and 0.779 (IQR 0.573-1.000) in the standard care group (P < 0.001). A score of 1.0 indicating absence of problems in all five EQ-5D-3L dimensions was achieved by 43% in the STROKE-CARD care group and 32% in the standard care group (relative risk 1.36; 95% CI: 1.20-1.54; P < 0.001). | The pragmatic and easily implementable STROKE-CARD care programme reduced cardiovascular risk and improved health-related quality of life and functional outcome in patients with acute ischaemic stroke or TIA |
| Wong and Yeung 2014 (57) | Randomised controlled trial  Control n=54, intervention n=54 | The intervention group received the transitional care program (TCP) which was commenced before discharge and lasted for 4 weeks after discharge. The TCP had 3 components 1) holistic care delivered by holistic care managers (HCMS), 2) transitional care track and 3) holistic care managers. | QoL (SF-36) at 4 and 8 weeks | SF-36 PCS:  After 4 weeks: Control 47.1 (1.2), intervention 47.9 (1.2)  After 8 weeks: Control 47.3 (1.4), intervention 48.6 (1.4)  F(P-value) after 4 weeks 13.76 (<0.001), after 8 weeks 37.60 (<0.001), interaction effect p=0.006  SF-36 MCS: After 4 weeks: Control 49.2 (1.1), intervention 49.9 (1.0)  After 8 weeks: Control 49.4 (1.4), intervention 49.9 (1.0)  F(P-value) after 4 weeks 13.72 (<0.001), after 8 weeks 7.67 (0.007), interaction effect p=0.591 | The TCP improved QoL and reduced depression. |
| Wu, 2020 (58) | Randomised controlled trial  N=64 (Control n=31, int n=30) | Home remote rehabilitation based on a collaborative care model creating a personalised plan delivered through videoconferencing (pathway) | QoL (SSQOL) at 4,8 and 12 weeks | Overall SSQOL scores:  4 weeks: 148.97±5.22 intervention, control 137.23±4.57  8 weeks: 174.07±4.71 intervention, 155.52±4.44 control  12 weeks: 190.57±5.09 intervention, 175.90±5.78 control  P<0.001 | Significant improvements in QoL for intervention |
| Xi, 2017 (59) | Randomised controlled trial  N=90 (Control n=45, int n=45) | Home remote rehabilitation based on a collaborative care model creating a personalised plan delivered through videoconferencing (pathway) | QoL (SF-36) at 6 months | Significant differences (P<0.05) versus control for 6/8 SF-36 domains excluding social functioning (SF) and bodily pain (BP) | Significant improvements in QoL |
| Yan et al., 2021 (29) | Open-label, two-arm cluster-randomized controlled trial  villages n=50, participants n=1299 (Control n=615, int n=611) | Primary care based app (SINEMA) to improve communication between HCPs and patients (mhealth) | QoL (EQ-5D-5L) at 12 months | Change in health-related quality of life score, mean (SD)§  Intervention 0.01 (0.15), control −0.03 (0.14)  Minimally adjusted model estimate (95% CI): 0.04 (0.01, 0.06), P=0.006  Fully adjusted model estimate (95% CI):  0.04 (0.01, 0.06), P=0.008  §Health-related quality of life was measured by using EQ5D-5L and was converted into a utility score based on the Chinese value set. | Significant improvements in QoL |

## Readmission

Table S5: Analysis of rehospitalisation

| **Study details** | **Design/Patients** | **Intervention** | **Outcome** | **Results** | **Comments** |
| --- | --- | --- | --- | --- | --- |
| Avci and Gozum, 2023 (60) | Parallel-group, assessor-blind, monocenter randomized controlled trial  Control n = 60 (30 patients and 30 caregivers), Intervention n = 66 in (33 patients and 33 caregivers) | Interviews and education (web-based training) for caregivers and patients | Readmission rates (12 weeks) | Hospital return status of stroke patients:  Intervention Group (n = 33): Yes = 5 (15.2%), no = 28 (84.8%)  Control Group (n = 30): Yes = 7 (23.3%), no = 23 (76.7%) | Readmission and pressure ulcers were less common in stroke patients in the intervention group compared with the control group. |
| Buckley et al., 2022 (6) | Retrospective observational study  N=836,923  Propensity matched cohorts:  Control n=2909, intervention n=2909 | Exercise-based cardiac rehabilitation | 2-year incidence of rehospitalisation | Rehospitalisation at 2-years was proportionally lower with 40.7% (n=1,185 of 2,909 patients) in the exercise-based cardiac rehabilitation cohort compared to 51.8% (n=1,507 of 2,909 patients) in the controls (OR 0.64, 0.58-0.71) | Significant reduction in rate of rehospitalisation with intervention (36%) |
| Bushnell et al., 2021 (7) | COMPASS was a cluster-randomized trial of the effectiveness of the COMPASS-TC intervention (INV) compared with usual care (UC)  Participating hospitals: control n=20, inv n=20 Patients: Control n=1193, inv n=1069 | Post-acute care pathway (full pathway) follow-up with the advanced practice provider and nurse coordinator in clinic within 7 to 14 days post discharge, an individualized electronic care plan, and referral to community services | 30- and 90-day unplanned readmissions, 1 year all-cause readmissions | 30d admissions: Intervention 105 (9.9%), control 103 (8.7%), OR 1,20 (95% CI: 0.83-1.74)  60d readmissions: Intervention 210 (19.9%), control 222 (18.9%), OR 1.10 (95% CI: 0.84-1.41)  1 year all-cause readmissions: Intervention 485 (45.4%), control 516 (43.3%), HR 1.06 (95% CI: 0.95-1.77)  1 year stroke readmission: Intervention 67 (6.3%), control 62 (5.2%), HR 1.26 (95% CI: 0o.0-1.77) | No difference in readmission rates between groups |
| Chen et al., 2016 (61) | Quasi-experimental controlled study  Control n=173, intervention n=168 | Individualised transitional care model based at home | Readmission (1 month) | Readmission: Intervention 2/168 (1.19%) vs. control 11/173 (6.36%), Chi^2^ = 5.30, p = 0.02) | The intervention was associated with significantly lower readmission rates. |
| Cuccurrullo et al., 2022 (62) | Prospective matched cohort study  Control n=88, intervention n=105 | SRP program: Physician Visits, Outpatient Therapy, and Modified Cardiac Rehabilitation (management of blood pressure (BP), cholesterol control, reduction of blood sugar, getting active, eating better, losing weight, and smoking cessation) Lifestyle | Readmission rates (1 year) | All cause readmission: control 59/88 (67%), intervention 47/105 (45%), difference in proportion 22%, HR 0.51 (0.31-0.83), p=0.006 | Acute care hospital readmissions were reduced in stroke survivors who participated in SRP. |
| de Belvis, 2019 (11) | Pre-post retrospective observational study  N=1,009  Control n=483, intervention n=526 | 5-stage clinical pathway: (1) initial framing; (2) evaluation/staging; (3) treatment; (4) rehabilitation; and (5) follow-up/monitoring (full pathway) | 30-day readmission rate (Patients not registered in the Italian NHS) | Pre-intervention (control) 7.4%, intervention 7.0%, Δ% -5.7, P=0.85 | No statistically significant decrease in readmission rates within 30 days |
| Deutschbein, 2020 (12) | Pragmatic non-randomised controlled trial  N=361 (intervention n=145, control n=270) | Individualised case management, with regular face-to-face visits as part of a comprehensive individualised programme (PT, OT, support required), regular monitoring and adjustment (full pathway) | Readmissions within 12 months (incidence ratio [IR]) per 100 person months | Intervention: 4.03 (95% CI: 2.58–6.28) Control: 9.56 (95% CI: 7.99–11.43)  Incidence rate ratio (IRR) 0.45 (95% CI: 0.31-0.65) unadjusted, 0.42 (95% CI: 0.29-0.62), both P<0.001 | Significantly lower rates of readmission in intervention group |
| Fu, 2020 (14) | Prospective, randomised, open trial of two active and one control interventions with blinded endpoint assessment  N=400 (Primary analysis: control n=125, T1 n=123, t2 n=133) | One-to-one qualitative assessment of patient views and needs, education and goal setting | Readmission within 12 months (OR) secondary outcome | Readmission by 12 months, N/N (%): Control 53/130 (40.8), TC1 54/132 (40.9), TC2 41/138 (29.7), Odds ratio difference (95% CI) TC2 + TC1 versus control 0.79 (0.51 to 1.21), p=0.27 | No difference in readmission rates |
| Peng, 2017 (20) | Propensity score-matched case-control study using the National Health Insurance data  Original cohort: control n=3159, int n=1480 Matched Cohort: control n=657, int n=657 | PAC program measuring activities of daily living, nutrition, exercise, mobility and language | 90-day readmission event rates (events/total patient number) | 90-d readmission events Original cohort Intervention: 161/1480 (10.9%), control: 674/3159 (21.3%), aOR 0.47 (95% CI: 0.39-0.57), P<0.01  Matched cohort Intervention: 73/657 (11.1%), control: 138/657 (21.0%), aOR 0.47 (95% CI: 0.34-0.64), P<0.01  90-d readmission events for stroke Original cohort Intervention: 41/1480 (2.8%), control: 254/3159 (8.0%), aOR 0.32 (95% CI: 0.23-0.45), P<0.01  Matched cohort Intervention: 14/657 (2.1%), control: 58/657 (8.8%), aOR 0.22 (95% CI: 0.12-0.41), P<0.01 | Significantly lower hospital 90-day readmissions and stroke-related readmissions |
| Reeves, 2019 (27) | Open-label (unblinded) 3-group parallel-design clinical trial  N=320 (Group 1 (UC): Global-10 n=77, PAM n=76, Group 2 (SWCM) Global-10 n=82, PAM n=79, Group 3 (SWCM + MISTT website) Global-10 n=81, PAM n=78)  UC, usual care; MISTT, Michigan stroke transitions trial; PAM, pain activation measure; SWCM, social worker–led case management | Home-based social worker–led case management (SWCM) program with or without a website, comprising biopsychosocial assessment followed by development of a personalised plan to address unmet needs (pathway, psychological) | Rehospitalisation at 90 days | 90-day hospital readmissions (n=265)  UC 17 (20%)  SWCM 20 (23%) OR 1.21 [0.59–2.51]  SWCM+MISTT Website 18 (20%) OR 1.03 [0.49–2.16]  Type 3 χ2 global test: P=0.85 | Hospital readmission occurred in 55 (20.8%) of the 265 subjects, but rates did not differ between treatment groups (P=0.85) |
| Schwartzbach et al., 2023 (SANO) (22) | Prospective, open-label, cluster-randomised controlled trial  Control n=1283, intervention n=1203 | 1-year patient-centred integrated care intervention including regular follow-up, lifestyle, goal setting and motivational interviewing (SANO) | Readmission to hospital at 12 months | Readmission to hospital within 1 year of index stroke: Control: 383/1216 (31·5%); Intervention: 375/1169 (32·1%): OR 1·01 (95% CI 0·74–1·38); aOR: 0·99 (95% CI 0·73–1·37) | This study demonstrated that the SANO programme had positive effects on the control of some cardiovascular risk factors in patients with stroke, but this did not translate into a reduction in the rate of major cardiovascular events 1 year after the first ischaemic stroke. |
| Swanson, 2019 (23) | Retrospective cohort study  Control: n-=2605, rehab only n=689, home nursing only n=535, home nursing and rehab n=898 | Standalone home nursing or rehabilitation, or a combination of both, with or without GP follow-up visits (pathway) | 90-day and 1-year readmission | Adjusted HR 90-day:  Rehab 1.45* (95% CI: 1.06–1.97)  Home nursing 1.08 (95% CI: 0.74–1.57)  Rehab + Home nursing 0.95 (95% CI: 0.67–1.34)  Adjusted HR 1-year:  Rehab 1.80* (95% CI: 1.45–2.22)  Home nursing 1.31 (95% CI: 1.02–1.66)  Rehab + Home nursing 1.46 (95% CI: 1.16–1.84)  *Significant difference to home nursing with rehabilitation, p < 0.05 | Patients receiving home nursing and/or rehabilitation had higher unadjusted 90- and 365-day readmission rates than those without services (HR from 1.87 to 2.63 depending on analysis, p < 0.001), but the 90-day differences disappeared after risk adjustment, except for patients receiving only rehabilitation |
| Wang et al., 2021 (63) | Randomised controlled trial  N=184 (Control n=82, int n=84) | Pharmacist-delivered care program providing inpatient and outpatient care - education focused on medication therapy management (MTM), disease state management, and lifestyle for secondary prevention of ischemic stroke | Readmission was any event leading to hospitalization, including stroke recurrence or poor control of risk factors. The rates of re-hospitalizations were calculated and compared between groups at 6-month discharge. | Comparison of re-admission rates between the two groups at 6-month discharge (n = 166)  Recurrent stroke, n (%): Intervention 3 (3.57), control 8 (9.76); p=0.11  Poor control of risk factors, n (%): intervention 1 (1.19), control 5 (6.10); p=0.20  Others, n (%): Intervention 2 (2.38), control 2 (2.44), p=1.00  Total, n (%): Intervention 6 (7.14), control 15 (18.30); p=0.03 | Significantly fewer patients were re-admitted to the hospital in IG than CG (7.14% vs. 18.3%, P = 0.03). |
| Wong and Yeung 2014 (57) | Randomised controlled trial  Control n=54, intervention n=54 | The intervention group received the transitional care program (TCP) which was commenced before discharge and lasted for 4 weeks after discharge. The TCP had 3 components 1) holistic care delivered by holistic care managers (HCMS), 2) transitional care track and 3) holistic care managers. | Readmission at 8 weeks | Readmission rates at 8 weeks: Control 8/54 (14.8%), intervention 4/54 (7.4%) p=0.358 | Readmission rates in the intervention group were lower but this did not reach statistical significance |

## Depression and anxiety

Meta-analysis also showed that depression scores were significantly lower with integrated care than control regardless of the scale being used or the follow up time (SMD = -0.16, 95% CI: -0.26 to -0.06, p=0.001, I^2^ = 0%, Figure S3).

Meta-analysis was conducted in 3 (n=606) trials reporting proportions of patients with anxiety using HADS-A (Figure S4). Integrated care was associated with a significant reduction in the proportion of patients with anxiety compared with control (RR 0.75, 95% CI: 0.57-0.99, p=0.04, I^2^ = 17%, Figure S4). Similar findings were reported in 2 studies using Zung’s SAS (RR 0.50, 95% CI: 0.32-0.79 , p=0.003, I^2^ = 0%, Figure S5). (64, 65).

Anxiety scores were also lower with integrated care interventions according to a meta-analysis of 4 studies (n=940) (Figure S6). SMD was -0.35 (95% CI: -0.70-0.01, p=0.05, I^2^ = 84%).


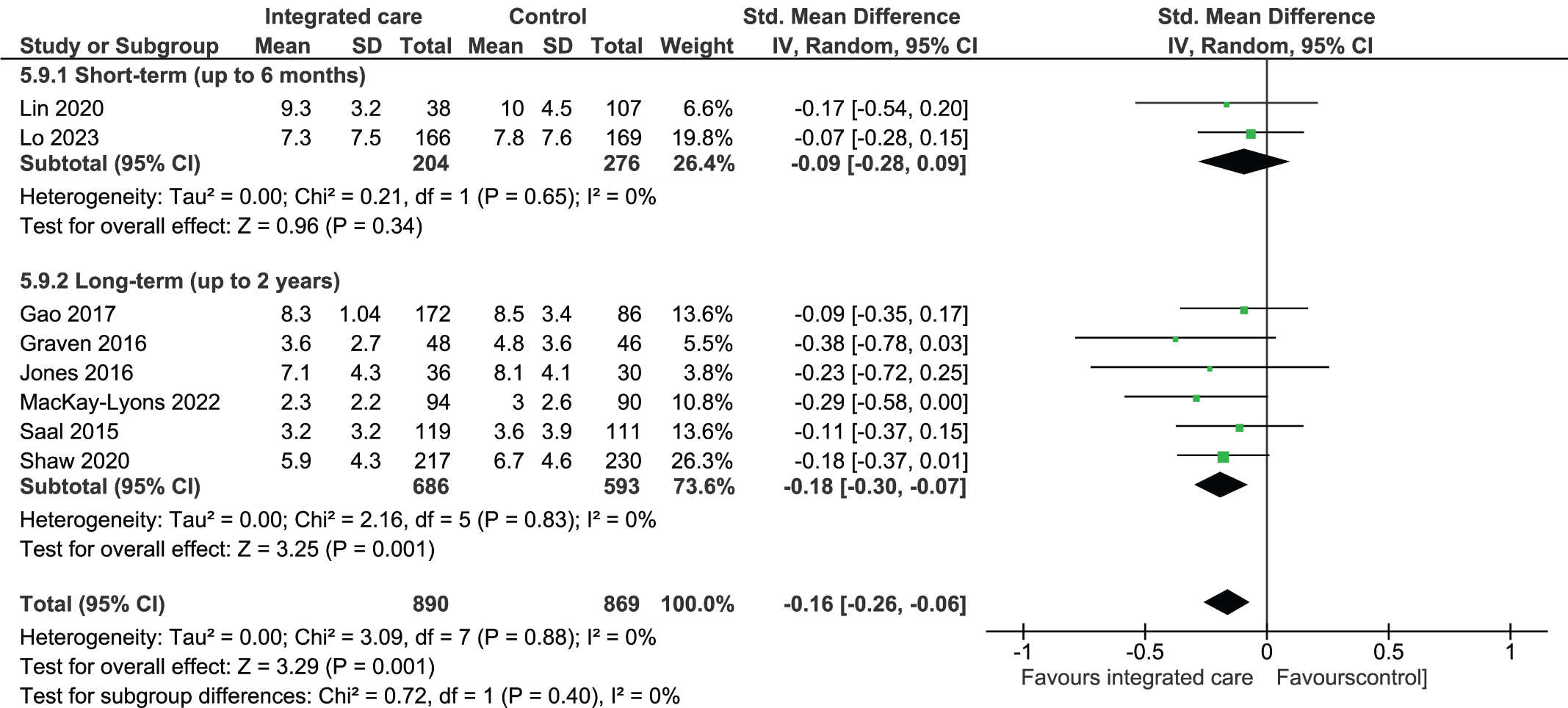


**Figure S3: Comparison of integrated care with control for depression scores by follow-up time**


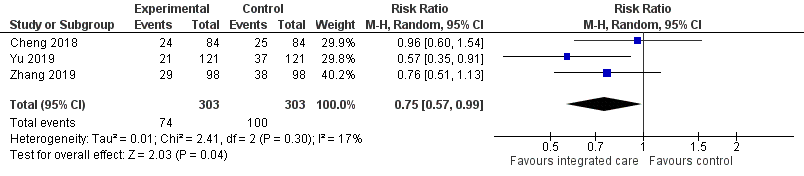


**Figure S4: Comparison of integrated care with control for anxiety (HADS-A Anxiety Scale)**


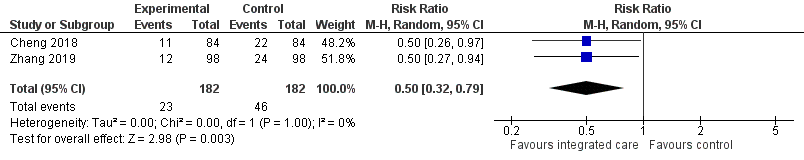


**Figure S5: Comparison of integrated care with control for anxiety (Zung’s SAS Scale)**

**
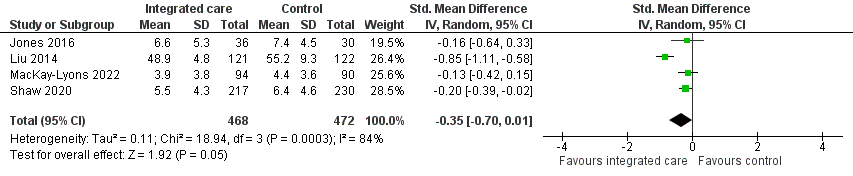
**

**Figure S6: Comparison on integrated care with control for anxiety scores (SMD)**

Table S6: Analysis of depression and anxiety

| **Study details** | **Design/Patients** | **Intervention** | **Outcome** | **Results** | **Comments** |
| --- | --- | --- | --- | --- | --- |
| Abdul Aziz, 2020 (30) | Pragmatic cluster randomised controlled trial-within trial at public primary care health centres  Control n=65, iCaPPS n=86 | Screening for complications, QoL, depression | Depression screen PHQ scores | Control:  Baseline ≥10 6 (9.2%), <10 59 (90.8%)  Exit ≥10 1 (1.2%), <10 85 (98.8%)  Intervention:  Baseline ≥10 6 (7.0%), <10 80 (93.0%)  Exit ≥10 6 (7.0%), <10 80 (93.0%)  Fisher’s exact tests:  Baseline 0.793  Exit 0.240 | No significant difference in depression between groups |
| Bragstad et al., 2020 (31) | Multicenter, prospective, randomized, assessor-blinded, controlled trial with two parallel groups  Gave consent n=353, randomised n=322  Control n=156, intervention n=166 | Dialogue-based intervention to promote psychosocial wellbeing (psychological) | Yale-Brown single-item questionnaire | Feeling sad or depressed (Yale; N (%))b  Intervention group  T1 29 (17.4), T2 37 (22.3), T338 (22.9)  Control group  T1 43 (27.6), T2 36 (23.1), T3 37 (23.7)  Between group differences at 12 months 0.96 (0.55, 1.68), OR 0.890c | No difference in depression at 12 months |
| Brandal, 2019 (66) | Prospective, randomised, open­label, blinded endpoint, event-­driven trial  N=99833 (Control n=27554, intervention n=1432) | Early supported discharge (ESD) | Patient recorded outcome measures (PROMS) | Depression n (%)  Seldom Intervention 1083 (75.6), Control 20,819 (75.6)  Often intervention109 (7.6), control 3118 (13.3), p=0.001 (OR 0.68 95% CI: 0.55 - 0.84)  Missing: Intervention 240 (16.8), Control 3617 (13.1) | Significantly improved satisfaction with rehab after discharge, independence in mobility, toileting and dressing, significantly less depression |
| Cheng, 2018 (67) | Randomised controlled trial  N=201 (CRT n=84, control n=84) | Patient and family member education, cognitive training (psychological), rehabilitation training, regular follow-up | HADS anxiety/depression score  SAS anxiety score, SDS depression score | HADS anxiety score change at 12 months: -0.23 ± 0.20 (CRT) vs 0.62 ± 0.17 (control), p=0.002  SAS anxiety score change at 12 months: -0.23 ± 0.20 (CRT) vs 2.00 ± 0.77 (control), p=0.006  HADS depression score change at 12 months: -0.71 ± 0.23 (CRT) vs 0.44 ± 0.18 (control), p<0.001  SDS depression score change at 12 months: -2.10 ± 1.10 (CRT) vs 2.30 ± 0.88 (control), p=0.002 | Significantly less cognitive impairment, anxiety and depression |
| Duncan, 2020 (13) | Pragmatic cluster-randomized trial  6024 patients, 41 hospitals (Hospitals n=40, patients intervention n=2689, control n=3193) | Education, secondary prevention, rehabilitation, recovery, referrals to community-based resources, and caregiver support services by telephone follow-up | Depression/no depression. Depression measured by the PHQ-2. Scores range from 0 to 6; higher scores corresponding to less favourable outcomes. Absence of depression considered to be a score of ≤2. | No depression, n (%): Intervention 1025 (78.3), control 1122 (76.6); OR 0.97 (0.74 to 1.26) | No difference in depression |
| Ellis-Hill, 2019 (68) | Two-centre, 24-month, parallel-arm RCT with qualitative and economic components  N=56 (Control n=25, intervention n=22) | Arts and health practitioner-led group sessions (psychosocial and wellbeing) | Warwick-Edinburgh Mental Well-being Scale (potential range 14–70, higher scores greater well-being)  HADS | Warwick-Edinburgh Mental Well-being Scale  Mean difference (95% CI) in change from baseline (unadjusted): 2.25 (−2.83 to 7.32), effect size 0.23  Mean difference (95% CI) in change from baseline (adjusted for centre and baseline score): 1.14 (−3.42 to 5.70), effect size 0.12  Hospital Anxiety and Depression Scale-anxiety subscale  Mean difference (95% CI) in change from baseline (unadjusted): −0.47 (−2.48 to 1.54), effect size -0.12  Mean difference (95% CI) in change from baseline (adjusted for centre and baseline score): −0.55 (−2.39 to 1.28), effect size -0.14  Hospital Anxiety and Depression Scale-depression  Mean (SD) n baseline: Control 5.0 (  Mean difference (95% CI) in change from baseline (unadjusted): −1.82 (−3.42 to −0.22), effect size -0.56  Mean difference (95% CI) in change from baseline (adjusted for centre and baseline score): −1.46 (−3.12 to 0.21), effect size -0.45 | Positive trend towards reduced depression and anxiety with intervention but not significant |
| Feng, 2021 (69) | Randomised controlled trial  N=120 (Control n=60, intervention n=60) | Trained multidisciplinary team providing an intervention plan based on the patient’s condition and treatment-related information, and arranging specific plans for family visits and telephone follow-ups, continued support and follow-up in the community (full pathway) | Zung’s Self-rating Anxiety Scale (SAS) and Self-rating Depression Scale (SDS) scores | Comparison adverse mood between the two groups before and after intervention (x±s, points)  Before the intervention: control SAS scores 58.27±6.03, SDS scores 61.06±5.37; intervention group SAS scores 58.41±5.98, SDS scores 61.28±5.62  t value SAS 0.128, SDS 0.219  P value SAS 0.899, SDS 0.827  3 months after the intervention: control SAS scores 53.37±5.26, SDS scores 55.34±6.11; intervention group SAS scores 47.01±5.14, SDS scores 48.71±5.42  t value SAS 6.698, SDS 6.288  P value SAS 0.000, SDS 0.000 | Significantly lower anxiety and depression with intervention |
| Gao, 2017 (70) | A single-blind, randomised, controlled trial that compared three intervention groups, with subgroups stratified by time after stroke  N=273 (Group A n=91, Group B n=91, group C n=92)  Analysis groups: Group A n=86, Group B n=85, Group C n=87 | Citalopram, psychological intervention for depression, cognitive behavioural education | HAMD 17-item scale, Bech-Rafaelsen Melancholia Scale (MES) | Comparison of outcome between the three groups – total sample  MES, mean (SD): Group A 10.0 (2.8), Group B 9.0 (2.2), Group C 9.6 (2.1); Comparison (A, B, and C) p=0.02, comparison (A and B) p=0.02, Comparison (A and C) p=0.51 HAMD17, mean (SD): Group A 8.5 (3.4), Group B 8.1 (2.4), Group C 8.5 (2.7); Comparison (A, B, and C) p=0.60, comparison (A and B) p=0.79, Comparison (A and C) p=1.00  Outcome according to the depression scales with the stratified subgroups.  3-6 months after stroke MES and HAMD A vs B (both P=0.02)  6-9 months after stroke MES A, B and C (P=0.03), A vs C (P=0.01) | Significantly improved depression scores but only for MES and MES/HAMD at certain times in subgroup analysis (no difference in HAMD overall) |
| Graven et al., 2016 (71) | Prospective randomised controlled trial  Control n=46, intervention n=48 | Physiotherapist-led comprehensive multifactorial care pathway individualised to each participant | Depression (Geriatric depression score [GDS-15]) at 12 months | At 12 months, the mean GDS-15 score was lower in the intervention group (3.6±2.7) compared to the control group (4.8±3.6). A lower proportion of participants in the intervention group (14.6%) had significant depressive symptoms (GDS-15 ≥6) at 12 months compared to the control group (34.8%). At 12 months. | Positive impact of individualised, goal-centered, comprehensive poststroke management for reducing depressive symptoms during the first year poststroke. |
| Gun Young, 2021 (72) | Open label non-randomised study with non-equivalent control group design  N=50 (Control n=23, inv n=20) | Integrated management programme assessing physical function (structured exercise), cognitive function, and depression | Beck Depression inventory at 4 and 8 weeks | Depression, mean (±SD)  Pre-test  Exp. 26.90±6.63, Control 24.26±6.13  Post-test (4 wk)  Exp 19.05±4.49, control 21.00±4.72  Post-test (8 wk)  Exp 12.00±2.60, control 17.826±4.76  G 1.62, p=0.211  T 171.35, p<.001  G*T 27.02, p<.001 | Significant reduction in depression with intervention |
| Hjelle, 2019 (40) | Multicentre, prospective, randomised controlled trial  Enrolled n=353, randomised n=322 (Control n=156, inv n=166) | Dialogue-based intervention delivered in patient home by nurse/OT (psychological) | Yale-Brown single-item questionnaire at 6 months | Depressed (Yale), n (%)  T1: Intervention 29 (17.4), control 43 (27.6)  T2: Intervention 37 (22.3), control 36 (23.1)  Between group difference: OR 1.248 (95% CI: 0.64-2.41), P=0.507 | No difference in depression between groups |
| Jones et al., 2016 (41) | Cluster randomised controlled trial  Control n=30, intervention n=36 | Bridges self-management program (SMP) comprising one-to-one rehabilitation sessions and stroke workbook | Hospital anxiety and depression scores at 12 weeks | HADS-A scores:  Baseline: control 7.4±5.1, intervention 7.5±5.3  6 weeks: control 7.3±4.9, intervention 7.5±4.9  12 weeks: control 7.4±4.5, intervention 6.6±5.3  HADS-D scores:  Baseline: control 7.1±3.4, intervention 6.9±4.2  6 weeks: control 8.2±4.1, intervention7.1±4.5  12 weeks: control 8.1±4.1, intervention 7.1±4.3 | No significant differences in QoL or anxiety/depression |
| Jung, 2021 (73) | Non-randomised single arm trial with historical control group  N=313 (Control n=96, int n=227)  Analysis: Control n=96, int n=88 | Exercise based intervention tailored to each patient | Mental component summary (MCS) of the Short-Form Survey 36 (SF-36), and Beck Depression Inventory (BDI) at 4 weeks | BDI: T0: Intervention 12.08 ± 10.30, Control 14.45 ± 11.60  T1: Intervention 9.20 ± 9.18, Control 14.73 ± 12.18  P=0.087 (95% CI: −2.21 to 4.31)  MCS: T0: Intervention 34.28 ± 9.48, control 35.56 ± 11.84  T1: Intervention 38.53 ± 10.19, control 37.32 ± 11.37  P=0.074 (95% CI: −9.39 to 0.45) | No significant differences in BDI or NCS between groups |
| Khramov, 2021 (44) | Prospective randomized controlled parallel-group study  N=122 (Control n=59, int n=63) | Exercise, massage, PT and OT, social adaptation, goal setting | Beck depression inventory at 6 months | In the comparison group, the Beck Depression Inventory score declined to 15.3 ± 1.8 points at 3 months. The score was significantly lower in the main group (p = 0.008), equalling 12.7 ± 1.6 points. At 6 months, the situation was the same: group 1 scored 15.8 ± 1.4 points, group 2 scored 13.1 ± 0.9 points (p < 0.001) | More pronounced improvement in BDI scores for intervention group |
| Lee, 2018) (74) | Open-label parallel group study  N=31 (Control n=17, int n=14) | Horticulture-based exercise therapy | Geriatric depression scale (GDS 5/15) at 6 weeks | GDS, mean (SD): Pre-test: control 6.5 (3.3), Intervention 7.0 (4.5)  Post-test: Control 7.4 (3.4), Intervention 4.6 (3.7); P=0.044 | Stroke patients in the HT group showed significantly improved upper limb function, hand force, balance, fall efficacy, activities of daily living, and decreased depression (P < 0.05) |
| Lin, 2020 (75) | Randomised controlled trial  Control n=107, int n=38 | Supervised virtual reality training in addition to early rehabilitation exercise | HADS at baseline and day of discharge (7-21 days) | Depression:  Baseline: Int 12.1 ± 2.5, control 10.3 ± 4.8; t=5.13, p=0.025  After: Int 9.3 ± 3.2, control 10.0 ± 4.5; t=0.78, p<0.001 | Significantly decreased anxiety and depression scores with intervention |
| Liu et al., 2014 (15) | Multi centre randomized controlled trial  Control n=122, intervention n=121 | Early intervention: Standard care but at 48 hours following ICH onset. | Anxiety (Zung’s self rating anxiety scale) | Zung’s self rating anxiety scale:  3 months: control 51.8 (5.8), intervention 51.8 (4.9), mean difference = 0  6 months: control 55.2 (9.3), intervention 48.9 (4.8), mean difference -6.4 | Commencement of rehabilitation within 48 hours of ICH significantly reduced hospital length of stay and improved long-term survival and morbidity outcomes when compared with standard practices |
| Lo et al., 2023 (76) | Randomised controlled trial  Control n=169, intervention n=166 | Virtual multidisciplinary stroke clinic including monthly follow-up calls and educational videos | Primary: self-efficacy  Secondary: depression (GDS) | Depression level (GDS): Baseline (T0): control 8.4 (6.9), intervention 10.2 (6.7)  3 months (T1): control 7.1 (6.7), intervention 8.2 (6.9)  6 months (T2): control 7.8 (7.6), intervention 7.3 (7.5), effect size 0.36 (95% CI: 0.13-0.60) | The intervention reduced depression but not significantly |
| MacKay-Lyons et al., 2022 (PREVENT) (77) | Three-site, single-blinded, randomized controlled trial  Control n=90, intervention n=94 | Participants randomized to the PREVENT group engaged in a multi-modal, case-managed program of exercise and education. | Anxiety and depression (HADS) | HADS-A 0-21:  Baseline: PREVENT 5.2±4.1, control 5.5±4.0  Post-treatment: PREVENT 5.1±4.1, control 4.4±3.8  6 months: PREVENT 4.6±3.8, control 4.5±3.9  12 months: PREVENT 3.9±3.8, control 4.4±3.6  Change from baseline between groups: -.9 (-2.1, 0,3)  HADS-D 0-21:  Baseline: PREVENT 3.4±2.8, control 3.5±3.2  Post-treatment: PREVENT 3.1±2.9, control 3.4±3.1  6 months: PREVENT 2.9±2.6, control 3.3±2.9  12 months: PREVENT 2.3±2.2, control 3.0±2.6  Change from baseline between groups: -.2 (-1.1, .7) | Significant between-group differences at post-intervention favored PREVENT group over UC: DBPrest (mean difference [MD]: -3.2 mmHg, 95% confidence interval [CI]: -6.3, -.2, P = .04) and LDL-C (MD: -.31 mmol/L, 95% CI: -.42, -.20, P = .02). Trends of improvement in PREVENT group were noted in several variables between baseline and 6-month follow-up but not sustained at 12-month follow-up.  Impact of PREVENT on vascular risk factor reduction was more modest than anticipated, possibly because several outcome variables approximated normative values at baseline and training intensity may have been sub-optimal. |
| Minshall, 2020 (50) | Randomised controlled trial  N=458 (Control: stroke patients n=39, carers n=40. 12-month assessment patients n=25, carers n=23  Int: stroke patients n=50, carers n=44. 12-month assessment patients n=27, carers n=18) | Program of personalized psychosocial support – Stroke Care Optimal Health Program (SCOHP) comprising education, self-management and reflective exercises | HADS at 3, 6 and 12 months | HADS, mean (SD)  Depressive symptoms  Stroke survivor  Baseline: Int 6.31 (4.20), control 6.40 (5.42); p=0.93  3 months: Int 6.19 (4.44), control 6.88 (5.09); p=0.59  6 months: Int 6.71 (3.85), control 6.40 (5.38); p=0.80  12 months: Int 6.57 (5.07), control 6.72 (5.51); p=0.91  Stroke carer:  Baseline: Int 5.14 (3.47), control 5.31 (4.27); p=0.86  3 months: Int 4.20 (2.64), control 6.10 (5.35); p=0.16  6 months: Int 5.27 (3.80), control 5.52 (5.25); p=0.85  12 months: Int 5.33 (3.92), control 5.77 (5.01); p=0.76  Anxiety symptoms  Stroke survivor  Baseline: Int 7.33 (3.78), control 6.83 (4.55); p=0.62  3 months: Int 6.64 (3.58), control 7.00 (4.34); p=0.73  6 months: Int 7.35 (3.28), control 6.44 (5.05); p=0.41  12 months: Int 6.53 (3.72), control 6.96 (5.15); p=0.73  Stroke carer:  Baseline: Int 6.97 (4.14), control 6.86 (4.32); p=0.91  3 months: Int 6.10 (3.38), control 6.90 (4.05); p=0.52  6 months: Int 6.22 (3.82), control 6.47 (5.20); p=0.85  12 months: Int 6.27 (3.35), control 7.54 (5.90); p=0.42 | No significant differences between groups for QoL |
| Rafsten, 2019 (78) | Randomised controlled trial with blinded assessors  N=140 (Control n=71, int n=69) | Very early supported discharge comprising person-centred goal setting for achievement of identified aims (pathway) | HADS-A at 3 and 12 months post-stroke | No significant differences were found between the groups regarding anxiety at three or 12 months post-stroke (p = 0.811) | No significant differences between groups |
| Reeves, 2019 (27) | Open-label (unblinded) 3-group parallel-design clinical trial  N=320 (Group 1 (UC): Global-10 n=77, PAM n=76, Group 2 (SWCM) Global-10 n=82, PAM n=79, Group 3 (SWCM + MISTT website) Global-10 n=81, PAM n=78)  UC, usual care; MISTT, Michigan stroke transitions trial; PAM, pain activation measure; SWCM, social worker–led case management | Home-based social worker–led case management (SWCM) program with or without a website, comprising biopsychosocial assessment followed by development of a personalised plan to address unmet needs (pathway, psychological) | PHQ-9 at 7 and 90 days | PHQ-9 (depression; n=378 observations)*– difference in differences (D-in-D) analysis; test of group × time interaction: P=0.97  Neuro-QOL anxiety (n=351 observations)* D-in-D analysis; test of group × time interaction: P=0.49  *Number of available observations differ between outcomes due to missing data | No differences in depression or anxiety |
| Rodgers, 2019; Shaw, 2020 (52, 53) | Parallel-group observer-blind multicenter individually randomized controlled trial  N=573 (Control n=231, int n=219) | Early supported discharge reviews (EXTRAS) including mobility, self-care, mood (psychological), cognitive function (pathway) | HADS at 1 and 2 years | Adjusted Difference in means EXTRAS Minus Usual Care Mean (95% CI)  Anxiety 12 months: −0.7 (−1.3 to 0.0)  Anxiety 24 months: −0.6 (−1.4 to 0.1)  Depression 12 months: −0.7 (−1.5 to 0.0)  Depression 24 months: −0.7 (−1.4 to 0.0) | The mean intervention group Hospital Anxiety and Depression Scale scores were not significantly different at 12 and 24 months. |
| Saal et al., 2015 (21) | Randomised controlled clinical trial  Control n=111, intervention n=119 | Post-discharge support including follow-up call and visit, education and training sessions | Depression (GDS) at 12 months | GDS: Intervention: Baseline - 2.7 (2.2); 12 month - 3.2 (3.2), Control: baseline - 3.6 (3.3); 12 month - 3.6 (3.9) Mean difference (95% CI) 0.4 (-0.4; 1.1) | No significant differences were observed between the intervention and control groups in depression and somatisation. |
| Teuschl et al., 2017 (79) | Prospective, Randomized, Open-Label, Blinded Endpoint multicenter, two-arm parallel group clinical trial  N=202 (Control n=87, int n=80) | BP measurement, lifestyle advice, exercise, cognitive training on a regular basis | Centre for Epidemiologic Studies Depression Scale (CES-D) at 12 and 24 months | Lifestyle summary score (LSS) and laboratory supported lifestyle score (LLSS) changes for CES-D at 12 and 24 months:  LSS  12 months: coefficient 0.02, SE 0.01, p=0.015  24 Months: 0.02, SE 0.01, p=0.104  LLSS  12 months: coefficient 0.05, SE 0.01, p=0.002  24 months: coefficient 0.02, SE 0.01, p=0.09 | Depression was significantly improved in the intervention group at 12 months but not at 24 months |
| Wong and Yeung 2014 (57) | Randomised controlled trial  Control n=54, intervention n=54 | The intervention group received the transitional care program (TCP) which was commenced before discharge and lasted for 4 weeks after discharge. The TCP had 3 components 1) holistic care delivered by holistic care managers (HCMS), 2) transitional care track and 3) holistic care managers. | QoL (SF-36), depressive symptoms (CES-D), readmission | CES-D-HK (median [10^th^-90^th^ percentile]):  Total after 4 weeks: Control 4 (1.5–8), intervention 2 (0–5), p<0.001  Total after 8 weeks: Control 4 (0–7), intervention 2 (0–4), p<0.001 | The TCP improved QoL and reduced depression. |
| Yu et al., 2019 (80) | Randomised controlled trial  N=242 (Control n=121, int n=121) | Intensive patient care program (IPCP) on cognitive impairment, anxiety, depression including comprehensive psychoeducation and psychonursing, cognitive rehabilitation training and mobile communication application (psychological, mhealth) | Hospital Anxiety and Depression Scale (HADS) anxiety (HADS-A) score, and HADS depression (HADS-D) score at 3, 6 and 12 months | HADS-A score change (M12-M0): control -0.4±1.6, int 0.4±1.5 (P<0.001), anxiety rate M0: control 24.0%, int 24.8% (P=0.881), M12: control 30.6%, int 17.4% (P=0.016)  HADS-D score change (M12-M0): control -0.02±1.7, int -1.3±2.2 (P<0.001), depression rate M0: control 39.7%, int 43.8% (P=0.515), M12: control 44.6%, int 31.4% (P=0.034) | Proportion of patients with anxiety and depression was significantly lower at M12 with intervention compared with control. Changes in HADS-A and HADS-D scores were also significant in favour of intervention (both P<0.001) |
| Zhang et al., 2019 (81) | Randomised controlled trial  N=196 (Control n=98, int n=98) | Intensive caregiver education program (ICEP) comprising intensive individualized education for patients and caregivers and psychological nursing for caregivers delivered by nurses in 1:1 sessions, caregivers received repeat sessions every 2 weeks | HADS-A, HADS-D, Zung Self-rating Anxiety Scale (SAS), and Zung Self-rating Depression Scale (SDS) at 3, 6 and 12 months | HADS-A score: change M12-M0 control 0.6±1.5, int -0.3±2.0 (P<0.001), anxiety M12: control 27.6%, int 29.6% (P=0.752)  SAS score: change M12-M0 control 2.3±7.2, int -3.6±9.6 (P<0.001), anxiety M12: control 24.5%, int 12.2% (P=0.027)  HADS-D score: change M12-M0 control 0.4±1.6, int -0.7±2.1 (P<0.001), depression rate M12: control 31.6%, int 30.6% (P=0.877)  SDS score: change M12-M0 control 2.5±8.2, int -2.3±10.1 (P<0.001), depression rate M12: control 38.8%, int 23.5% (P=0.021) | Anxiety score change (M12–M0), anxiety score at M12, and anxiety rate at M12 were decreased in the ICEP group compared with the Control group. Depression score change (M12–M0), depression score at M12, and depression rate at M12 were lower in the ICEP group compared with the Control group. Further subgroup analysis based on baseline features also provided similar results. |

## Lifestyle and risk factors

Meta-analysis was conducted on lifestyle and risk factors including systolic blood pressure (8 studies, n=4,477), smoking cessation (3 studies, n=1,346), total cholesterol (4 studies, n=993) LDL-cholesterol (5 studies, n=3,318) and BMI (4 studies, n=738). Overall, integrated care was associated with a significant benefit compared with control (Table S7) SMD -0.17, 95% CI: -0.25, -0.09, p<0.0001, I^2^ = 63%, Figure S7) and there were significant reductions in SBP and LDL-cholesterol (p=0.009 and p=0.1, respectively), but not for total cholesterol (p=0.29) or BMI (p=0.35). Four studies evaluated rates of smoking cessation however there was no difference between the proportion of patients stopping smoking between groups (RR 1.10, 95% CI: 0.93-1.31, p=0.27, I^2^ = 91%, Figure S8).


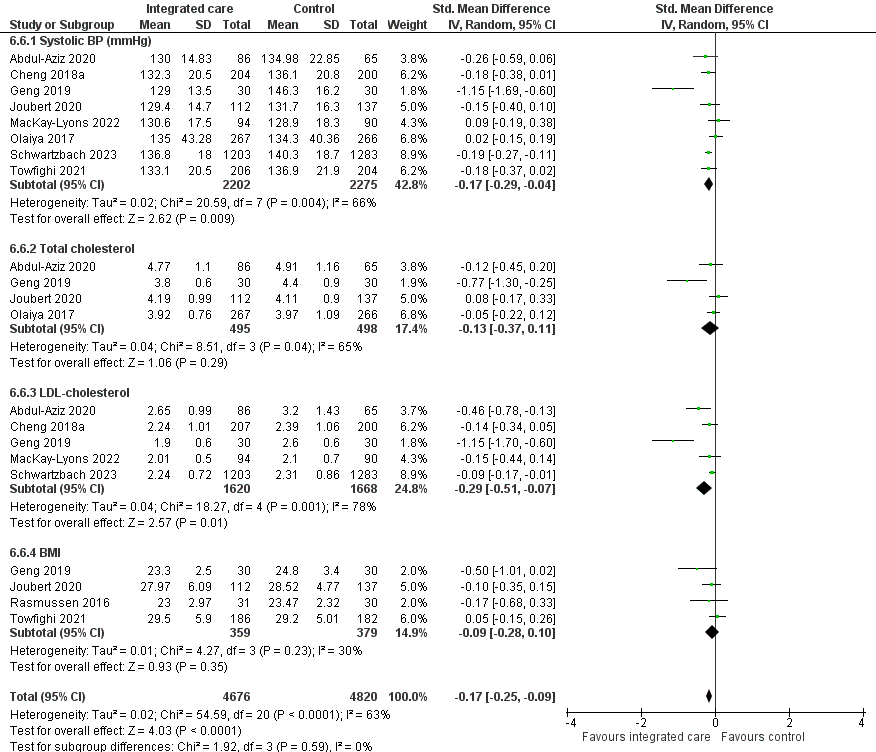


**Figure S7: Comparison of integrated care with control for cardiovascular risk factors**


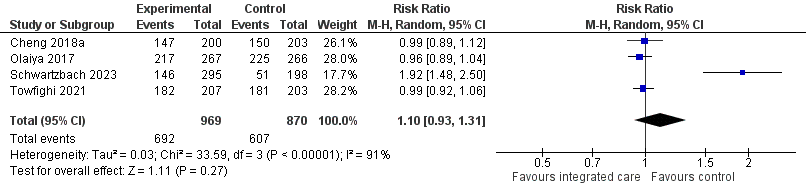


**Figure S8: Comparison of integrated care with control for smoking cessation**

Table S7: Analysis of lifestyle and/or cardiovascular risk factors

| **Study details** | **Design/Patients** | **Intervention** | **Outcome** | **Results** | **Comments** |
| --- | --- | --- | --- | --- | --- |
| Bath et al., 2018 (TARDIS) (2) | International (UK, Denmark, Georgia, NZ) prospective randomised open-label blinded end-point superiority clinical trial  Guideline (control) n=1540, Intervention n=1556 | Intensive treatment group given combined aspirin (load 300mg, maintenance 50-150mg daily, typically 75mg), clopidogrel (load 300mg, maintenance 75mg daily), and dipyridamole (200mg twice daily modified release, given orally; or 100mg three or four times daily). | Secondary: myocardial infarction at 90 days | Events: Intervention 11/1556 (0.7%), control 12/1540 (0.8%) | No difference in risk of recurrent cardiovascular disease |
| Cheng et al., 2018 (25) | Randomised controlled trial  N=407 (Control n=200, int n=207) | Medication modification relevant to stroke risk factors, tobacco cessation, physical activity, depression, and medication adherence | Change in SBP, LDL cholesterol, Physical activity, smoking cessation, 10-year risk of CV events | Differences in Changes From Baseline Between Control and Intervention Arms* (95% CI):  SBP −3.6 mm Hg (−9.3 to 2.2) p=0.55  LDL mg/dL, mean (SD) −10.2 mg/dL (−21.1 to −0.6) p=0.03  Smoking cessation 0.6 OR (0.3 to 1.3), p=0.58  Physical activity assessed as exercise ≥3 d per wk, n (%):0.9 OR (0.5 to 1.8), p=0.84  10 year risk of CV events: %, mean (SD) -0.8 (−3.1 to 1.6), p=0.98  *Repeated-measures mixed-effect models included baseline, 3-mo, and 12-mo values. | Among secondary outcomes, the only significant difference was that persons in the intervention arm were more likely to lower their low-density lipoprotein <100 md/dL (2.0 odds ratio; 95% CI, 1.1–3.5). All other between group differences were not significant  Depression was not reported |
| Geng, 2019 (38) | Randomised controlled trial  N=101 (Control n=30, intervention n=30) | Physical function, emotional function, lifestyle, medication adherence | Systolic blood pressure, BMI, cholesterol, physical activity, smoking and alcohol consumption at 6 months | Outcomes 6 months after discharge (mean [SD])  SBP (mm Hg): Intervention 129.0 (13.5), control 164.3 (16.2); P<0.001  BMI (kg/m2): Intervention 23.3 (2.5), 24.8 (3.4); P<0.001  Total cholesterol (mmol/l): Intervention 3.8 (0.6), control 4.4 (0.9); P<0.001  Triglycerides (mmol/l): Intervention 1.1 (0.60, control 1.2 (0.5); P=0.007  LDL (mmol/l): Intervention 1.9 (0.6), control 2.6 (0.6); P<0.001  HDL (mmol/l): Intervention 1.4 (0.3), control 1.3 (0.4); P=0.227  Physical activity: Intervention: 19.5 (3.7), control 15.9 (4.2); P<0.001  Smoking: Intervention 3.3 (0.7), control 2.9 (0.7), P=0.009  Alcohol consumption: Intervention 3.6 (0.6), control 2.8 (0.7), P=0.035 | Significant differences in SBP, BMI, cholesterol in favour of intervention but smoking and alcohol use were significantly lower in the control group |
| Greger, 2021 (82) | Single-centre retrospective chart review  Control n=4516, inv n=1274  Matched groups: Control n=171, inv n=171 | Pharmacist led medication review, counselling, risk factor management (lifestyle) | N (%) of patients counselled on medication adherence, added fish oil or vitamin C | Counselling on medication adherence:  Platelet function test (PFT) group: 24 (14%), control 5 (3%); P=0.008  Added fish oil or vitamin C: PFT group 39 (23%), control 5 (3%); P=0.0001 | Significantly improved medication adherence and added fish oil and vitamin C with intervention group |
| Joubert et al., 2020 (ICARUSS) (83) | Double blind randomized controlled trial  Control n=137, intervention n=112 | ICARUSS (Integrated Care for the Reduction of Secondary Stroke) intervention involving personalizing stroke risk factors for the stroke survivor and ongoing support from a stroke specialist and primary care physician vs usual care. | Risk factors (blood pressure, cholesterol, BMI, alcohol) | At the 12-month evaluation, there was a significant decrease in systolic blood pressure (129.4 [14.7]) from baseline (134.6 [16.7]) in the intervention group of 5.2mmHg (p<0.01), control 133.7 (17.0) at baseline to 131.7 (16.3) at 12 months, p=0.29.  Fasting total cholesterol (mmol/l):  Intervention: Baseline 5.00 (1.17), 12 month 4.19 (0.99)  Control: Baseline 4.36 (1.14), 12 month 4.11 (0.90)  BMI:  Intervention: Baseline 27.78 (5.38), 12 month 27.97 (6.09), p=0.50  Control: Baseline 27.45 (4.94), 12 month 28.52 (4.77), p=0.02  Alcohol (no of drinks per week) Intervention: Baseline 8.4 (16.5), 12 month 4.8 (10.1), p=0.04  Control: Baseline 6.0 (10.2), 12 month 4.6 (7.2), p=0.26 | In stroke survivors, the ICARUSS model was superior to usual care with respect to best-practice recommendations for traditional risk factors as well as behavioral and functional outcomes. |
| MacKay-Lyons et al., 2022 (PREVENT) (77) | Three-site, single-blinded, randomized controlled trial  Control n=90, intervention n=94 | Participants randomized to the PREVENT group engaged in a multi-modal, case-managed program of exercise and education. | CV risk factors including systolic BP, cholesterol, anxiety and depression (HADS) | Resting SBP mmHg:  Baseline: PREVENT 131.8±15.7, Control 131.7±18.4  Post-treatment: PREVENT 125.9±16.7, Control 129.3±18.1  6-months: PREVENT 126.8±15.1, control 128.2±16.8  12 months: PREVENT 130.6±17.5, control 128.9±18.3  Change from baseline between groups -3.5 (-8.8, 1.8)  Resting DBP mmHg  Baseline: PREVENT 76.6±11.1, control 74.9±9.8  Post-treatment: PREVENT 72.2±12.0, control 73.7±14.6  6 months: PREVENT 71.8±8.1, control 73.8±9.0  12 months: PREVENT 73.0±9.7, control 72.5±9.9  Change from baseline between groups -3.2 (-6.3, -.2), p<0.05  LDL-C Mmol/L:  Baseline: PREVENT 2.24±0.8, control 2.24±0.9  Post-treatment: PREVENT 1.94±0.8, control 2.23±0.8  6 months: PREVENT 1.96±0.6, control 2.21±0.8  12 months: PREVENT 2.01±0.5, control 2.10±0.7  Change from baseline between groups -.31 (-.42, -.20), p<0.05 | Significant between-group differences at post-intervention favored PREVENT group over UC: DBPrest (mean difference [MD]: -3.2 mmHg, 95% confidence interval [CI]: -6.3, -.2, P = .04) and LDL-C (MD: -.31 mmol/L, 95% CI: -.42, -.20, P = .02). Trends of improvement in PREVENT group were noted in several variables between baseline and 6-month follow-up but not sustained at 12-month follow-up.  Impact of PREVENT on vascular risk factor reduction was more modest than anticipated, possibly because several outcome variables approximated normative values at baseline and training intensity may have been sub-optimal. |
| Olaiya et al., 2017 (84) | Pragmatic multicentre, cluster- randomised, controlled trial, with blinded assessment of outcomes and intention-to-treat analysis  N=2516 (Control n=266, int n=267) | Individualised management programme comprising a chronic disease management (CDM) plan and education | Framingham risk score (modified for secondary outcomes). FRS includes cholesterol levels, systolic BP, use of antihypertensives, smoking status and uncontrolled diabetes | Between group differences for primary outcome variables (95% CI):  Systolic BP (mmHg) 0.05 (- 0.8, 1.6)  HbA1c (%)0.08 (-0.3, 0.5)  Total cholesterol (mmol/l) -0.008 (-0.1, 0.1)  HDL cholesterol (mmol/l) 0.02 (-0.02, 0.06)  Unadjusted and adjusted analyses of the effect of intervention on secondary outcomes, effect estimate (95% CI); p-value  Systolic BP (per mmHg): unadjusted 1.1 (0.1, 22.1); p=0.974, adjusted 2.1 (0.1, 41.9); p=0.620  Current smoking Unadjusted 2.2 (1.1, 4.5), p=0.031, adjusted 1.9 (0.9, 4.0), p=0.078  Current risky drinking Unadjusted 1.2 (0.5, 2.7); p=0.670, adjusted 1.4 (0.6, 3.3); p=0.440  Healthy eating  ≥5 servings of vegetables daily Unadjusted 0.6 (0.3, 1.2); p=0.171, adjusted 0.6 (0.3, 1.2); p=0.138  ≥2 servings of fruit daily Unadjusted 0.9 (0.6, 1.2); p=0.396, adjusted 1.0 (0.7, 1.4); p=0.843  <5 g salt daily Unadjusted 0.5 (0.2, 1.3); p=0.169, adjusted 0.6 (0.2, 1.4); p=0.216  Physically active Unadjusted 1.1 (0.6, 2.0); p=0.671, adjusted 1.1 (0.6, 2.0); p=0.719 | No significant between group difference for any outcome |
| Rasmussen et al., 2016 (85) | Interventional, randomised, safety/efficacy open-label trial  Control n=30, intervention n=31 | Multidisciplinary home-based pathway including exercise and goal setting | Secondary: BMI at 90 days | Body Mass Index (BMI), median (IQR): Intervention 23 (21–25); Control: 24 (22–25)  Body Mass Index (BMI) improvement: Intervention: 0 (-0.5–0) Control: −1 (-2–0) | Early home-based rehabilitation reduced disability and increased quality of life. Compared to standard care, home-based stroke rehabilitation was more cost-effective. |
| Schwartzbach et al., 2023 (SANO) (22) | Prospective, open-label, cluster-randomised controlled trial  Control n=1283, intervention n=1203 | 1-year patient-centred integrated care intervention including regular follow-up, lifestyle, goal setting and motivational interviewing (SANO) | Risk factors at 12 months | Blood pressure (including self-reported by interview) <140/90 mm Hg: Control: 657/936 (70·2%); Intervention: 809/1103 (73·3%); OR 1·18 (95% CI 0·79–1·76), aOR 1·08 (95% CI 0·73–1·59)  Blood pressure (physical examination only) <140/90 mm Hg† control: 468/679 (68·9%); intervention: 709/944 (75·1%); OR 1·34 (95% CI 0·86–2·09); aOR 1·34 (95% CI 0·88–2·04)  Diabetes control‡ HbA1c ≤7% (53 mmol/mol) Control: 123/218 (56·4%); Intervention: 144/218 (66·1%); OR: 1·10 (95% CI 1·02–2·21); aOR: 1·45 (95% CI 0·94–2·26)  Adherence to antithrombotic therapy targets§ Control: 1003 (88·3); Intervention: 1029 (89·4); OR 1·10 (95% CI 0·78–1·54); aOR 1·02 (95% CI 0·72–1·47)  Hyperlipidaemia control¶ Achievement of LDL cholesterol targets: Control: 444/729 (60·9%); Intervention: 526/782 (67·3%), OR 1·36 (95% CI 1·00–1·85); aOR: 1·65 (95% CI 1·22–2·22)  Smoking - Quit smoking - Control: 51/198 (25·8%); Intervention: 146/295 (49·5%); OR 3·13 (95% CI 1·72–5·70); aOR: 2·82 (95% CI 1·58–5·04) | This study demonstrated that the SANO programme had positive effects on the control of some cardiovascular risk factors in patients with stroke, but this did not translate into a reduction in the rate of major cardiovascular events 1 year after the first ischaemic stroke. Since a 1-year follow-up period might be too short to demonstrate a positive effect on recurrent cardiovascular events, longer-term effects need to be considered. |
| Teuschl et al., 2017 (79) | Prospective, Randomized, Open-Label, Blinded Endpoint multicenter, two-arm parallel group clinical trial  N=202 (Control n=87, int n=80) | BP measurement, lifestyle advice, exercise, cognitive training on a regular basis | Healthy lifestyle (reduced fat milk and spreads, eating fish, alcohol intake, physical activity, BMI, smoking cessation, systolic BP, Non-HDL cholesterol and fasting blood glucose) at 12 and 24 months | Changes in healthy lifestyle habits, physiological parameters, and low-risk summary scores from baseline to 12 months, and to 24 months in the intervention and the control group  Reduced fat milk  12 months: intervention +12.5% (p=0.031), control +5.8% (p=0.383), between group p=0.548  24 Months: intervention +12.5% (p=0.031), control -4.6% (p=0.503), between group p=0.062  Reduced fat spreads  12 months: intervention +16.3% (p=0.007), control +4.6% (p=0.481), between group p=0.273  24 Months: intervention +11.3% (p=0.108), control -2.3% (p=0.832), between group p=0.206  Fish ≥1/week  12 months: intervention +12.5% (p=0.021), control -4.6% (p=0.481), between group p=0.043  24 Months: intervention +10.0% (p=0.134), control +2.3% (p=0.832), between group p=0.616  Alcohol 1-6 times/week  12 months: intervention +7.5% (p=0.238), control -3.4% (p=0.648), between group p=0.323  24 Months: intervention 0.0% (p=1.000), control +5.8% (p=0.383), between group p=0.628  Physical activity  12 months: intervention +2.6% (p=0.754), control +1.1% (p=1.000), between group p=0.862  24 Months: intervention +1.3% (p=1.000), control -4.60.0% (p=1.000), between group p=0.862  Not currently smoking  12 months: intervention +2.5% (p=0.687), control +10.3% (p=0.004), between group p=0.118  24 Months: intervention +1.3% (p=1.000), control +9.2% (p=0.021), between group p=0.254  BMI  12 months: intervention -0.8 ± 2.0 (p=0.001), control -0.1 ± 2.1 (p=0.922), between group p=0.006  24 Months: intervention -0.8 ± 2.7 (p=0.007), control -0.2 ± 2.4 (p=0.938), between group p=0.036  Systolic BP (mm Hg)  12 months: intervention -1.6 ± 20.3 (p=0.529), control 2.4 ± 21.5 (p=0.494), between group p=0.397  24 Months: intervention -1.7 ± 20.1 (p=0.517), control -0.2 ± 22.9 (p=0.973), between group p=0.632  Non-HDL-C (mmol/l)  12 months: intervention -0.96 ± 1.16 (p<0.001), control -0.76 ± 1.64 (p<0.001), between group p=0.269  24 Months: intervention -0.97 ± 1.20 (p<0.001), control -0.88 ± 1.53 (p<0.001), between group p=0.732  Fasting blood glucose (mmol/l)  12 months: intervention -0.21 ± 1.51 (p=0.244), control -0.28 ± 2.01 (p=0.449), between group p=0.821  24 Months: intervention 0.01 ± 1.93 (p=0.611), control -0.06 ± 1.99 (p=0.600), between group p=0.499 | During the first 12 months, adherence to healthy lifestyle and adequately controlled physiological parameters (measured by summary scores) improved significantly in the intervention group compared to controls (p < 0.01). The consumption of reduced-fat milk (p = 0.031), reduced-fat spreads (p = 0.007), and fish (p = 0.021) increased in the intervention group from baseline to 12 months but not in controls. After 24 months, the group difference was significant for the lifestyle summary score but no longer for the combined laboratory lifestyle score. The number of smokers decreased significantly in the control but not in the intervention group. |
| Towfighi, 2021 (86) | Randomised controlled trial  N=487 (Control n=246, int n=241) | Advanced practice physician (APC) and community health worker (CHW) visits, culturally and linguistically tailored educational materials; vascular risk factor goal tools; and Chronic Disease Self-Management Program (CDSMP) workshops, medication adherence, self-management (pathway) | Systolic BP: mm Hg; Non-HDL, mg/dL; HbA1c, %; Log CRP, mg/dL, physical activity, IPAQ (International Physical Activity Questionnaire), MET; servings of fruit/veg, n (%), reduce/monitoring salt intake, n (%), not smoking and using antithrombotics, n (%) at 12 months | Differences in changes from baseline between usual care and intervention (95% CI):  SBP ≤130 mm Hg −3.3 (−14.9 to 8.8); p=0.57  SBP, mm Hg −1.7 (−6.4 to 2.9); p=0.46  Non-HDL, mg/dL −3.3 (−14.9 to 8.3); p=0.57  HbA1c,% −0.2 (−0.5 to 0.2); p=0.36  Log CRP, mg/dL −0.4 (−0.7 to −0.1); p=0.003  BMI 0.2 (−0.4 to 0.9); p=0.53  Physical activity, IPAQ, MET min/wk, median (IQR) 20 (−400 to 300); p=0.93  ≥5 daily servings of fruit/vegetables, No. (%)0.7 (−7.7 to 8.7); p=0.84  Reducing or monitoring salt intake, No. (%) 15.4 (4.4 to 26.0); p=0.004  Not smoking, No. (%)1.6 (−4.8 to 8); p=0.66  Using antithrombotic medication, No. (%)−0.6 (−6.7 to 5.3); p=0.80 | Mean (SD) systolic BP improved from 143 (17) mm Hg at baseline to 133 (20) mm Hg at 12 months in the intervention group and from 146 (19) mm Hg at baseline to 137 (22) mm Hg at 12 months in the usual care group, with no significant differences in the change between groups. Compared with the control group, participants in the intervention group had greater improvements in self-reported salt intake (difference, 15.4 [95% CI, 4.4 to 26.0]; P = .004) and serum CRP level (difference in log CRP, −0.4 [95% CI, −0.7 to −0.1] mg/dL; P = .003); there were no differences in other secondary outcomes. Although 216 participants (89.6%) in the intervention group received some of the 3 core components, only 35 participants (14.5%) received the intended full dose. |

## Adherence to intervention

Four (4) randomised studies included adherence to intervention as an outcome in meta-analysis. Interventions included triple antiplatelet therapy (2), combined exercise and cognitive training (87), individual transitional care models based at home (61), and self-management sessions and a stroke workbook (41). More participants were adherent to integrated care than control but this difference was not statistically significant (RR 1.31, 95% CI: 0.77, 2.23, p=0.32, I^2^=98%, Figure S9).


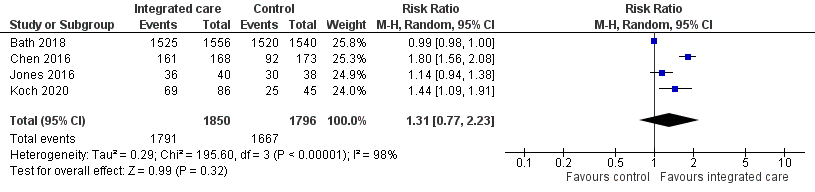


**Figure S9: Comparison of integrated care with control for adherence to intervention**

Table S8: Analysis of adherence to intervention

| **Study details** | **Design/Patients** | **Intervention** | **Outcome** | **Results** | **Comments** |
| --- | --- | --- | --- | --- | --- |
| Ellis-Hill, 2019 (68) | Two-centre, 24-month, parallel-arm RCT with qualitative and economic components  N=56 (Control n=25, intervention n=22) | Arts and health practitioner-led group sessions (psychosocial and wellbeing) | Follow-up data and study completion rates in n (%) of patients | Study retention was good (with follow-up data available for 84% of participants) and high data completion rates (>80% for the candidate primary outcome measures). | High adherence rates to intervention |
| Greger, 2021 (82) | Single-centre retrospective chart review  Control n=4516, inv n=1274  Matched groups: Control n=171, inv n=171 | Pharmacist led medication review, counselling, risk factor management (lifestyle) | N (%) of patients counselled on medication adherence, added fish oil or vitamin C | Counselling on medication adherence:  Platelet function test (PFT) group: 24 (14%), control 5 (3%); P=0.008  Added fish oil or vitamin C: PFT group 39 (23%), control 5 (3%); P=0.0001 | Significantly improved medication adherence and added fish oil and vitamin C with intervention group |
| Koch, 2020 (87) | Prospective randomised study  N=131 (control n=45, int n=86) | Combined exercise and cognitive training (psychological) | Number of visits attended per week and any adverse events (AE) | The observed-over-expected visit ratio was significantly higher in the intervention than in the control group (0.74±0.30 versus 0.54±0.38; P=0.003). | Significantly higher adherence  Intervention: 69/86 (80%)  Control: 25/45 (56%) |
| Bath et al., 2018 (TARDIS) (2) | International (UK, Denmark, Georgia, NZ) prospective randomised open-label blinded end-point superiority clinical trial  Guideline (control) n=1540, Intervention n=1556 | Intensive treatment group given combined aspirin (load 300mg, maintenance 50-150mg daily, typically 75mg), clopidogrel (load 300mg, maintenance 75mg daily), and dipyridamole (200mg twice daily modified release, given orally; or 100mg three or four times daily). | Adherence to intervention over the first 7 days | First treatment:  Intervention 1391/1556 (89.4%), control 1193/1540 (77.5%), p<0.001  Any treatment:  Intervention 1525/1556 (98.0%), control 1520/1540 (98.7%), p=0.14 | Significantly more participants in the TARDIS group were adherent with the first treatment compared with control but at 7 days there was no significant difference in adherence rates. |
| Chen et al., 2016 (61) | Quasi-experimental controlled study  Control n=173, intervention n=168 | Individualised transitional care model based at home | Adherence to medication at 1 month | Medication compliance rates: intervention 161 (95.83%) vs. control 92 (53.18%), p = 0.004. | The intervention was associated with significantly higher medication compliance. |
| Jones et al., 2016 (41) | Cluster randomised controlled trial  Control n=30, intervention n=36 | Bridges self-management program (SMP) comprising one-to-one rehabilitation sessions and stroke workbook | Adherence to intervention | Thirty-nine participants (98%) completed baseline measures and 36 participants completed week 12 outcome measures (90%) in intervention sites, compared with 35 (92%) completing baseline outcomes and 30 (79%) completing week 12 outcomes measures in control sites. | No significant differences in QoL or anxiety/depression but responsiveness to intervention was shown |

## **Functional status (modified Rankin scale)**

Four studies included favourable outcome (defined as mRS≤2). Overall, there was no significant difference in rates of favourable outcome between groups (RR 1.02, 95% CI: 0.98, 1.07, p=0.35, I^2^ = 77%, Figure S10).


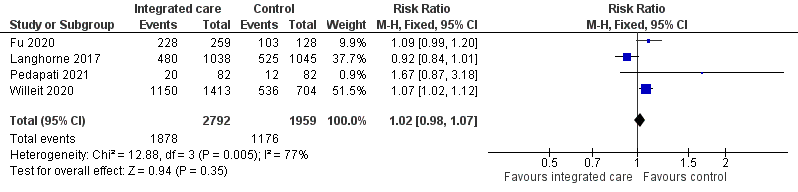


**Figure S10: Comparison of integrated care with control for favourable outcome**

Two non-randomised studies included disability or death (defined as mRS≥2). Overall, there was a significant difference in rates of disability or death in favour of integrated care (RR 0.85, 95% CI: 0.73, 1.00, p=0.0.04, I^2^ = 0%, Figure S11).


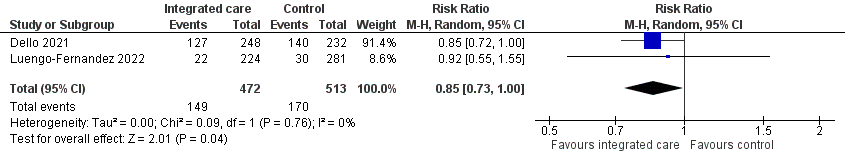


**Figure S11: Comparison of integrated care with control for disability or death**

Table S9: Analysis of functional status

| **Study details** | **Design/Patients** | **Intervention** | **Outcome** | **Results** | **Comments** |
| --- | --- | --- | --- | --- | --- |
| Bath et al., 2018 (TARDIS) (2) | International (UK, Denmark, Georgia, NZ) prospective randomised open-label blinded end-point superiority clinical trial  Guideline (control) n=1540, Intervention n=1556 | Intensive treatment group given combined aspirin (load 300mg, maintenance 50-150mg daily, typically 75mg), clopidogrel (load 300mg, maintenance 75mg daily), and dipyridamole (200mg twice daily modified release, given orally; or 100mg three or four times daily). | Primary: Incidence and severity of any recurrent stroke (ischaemic, haemorrhagic; assessed using the modified Rankin Scale) or TIA within 90 days by blinded telephone follow-up. | mRS at 90 days:  Death (mRS score of 6): Intervention 13/1540 (0.7%), control 9/1530 (0.6%)  mRS score of 4-5: Intervention 11/1540 (0.7%), control 9/1530 (0.6%)  mRS score of 2-3: Intervention 22/1540 (101%), control 23/1530 (1.5%)  mRS score of 0-1: Intervention 15/1540 (1.0%), control 18/1530 (1.2%)  Median mRS score [IQR]:  Intervention 1.0 [1.0, 2.0], control [1.0, 2.0] | No difference in mRS between groups |
| Bergh, 2023 (3) | Repeated measures analysis (pre-post) from a retrospective cohort of patients registered in the Norwegian Stroke Register (NSR).  N = 11009 included in study comparing functional outcome (5388 in 2017 and 5621 in 2019) | Standardised Care Pathway for stroke (SCP) based on National guidelines | Primary: change in mRS at 90 days | mRS at 90 days:  mRS score of 0-2: Intervention 4115/5621 (73.2%), control 3959/5388 (73.5%), p=0.77  mRS score of 3-5: Intervention 1504/5621 (26.8%), control 1429/5388 (26.5%) | No significant in change in mRS between the 2 years (OR=1.06, 95%CI 0.86, 1.30). |
| Bernhardt et al., 2015 (AVERT) (4) | Single-blind randomised controlled trial in 56 acute stroke units in 5 countries (Aus, NZ, Malaysia, Singapore, UK -Eng, Scot, Wales, NI)  Control n=1050, intervention n=1054 | Very early mobilisation (exercise and movement therapy) | Primary: favourable outcome (mRS)  Secondary: Change in mRS, mortality | Favourable outcome: intervention 480 (46%), control 525 (50%) (adjusted OR 0.73 [95% CI: 0.59-0.90]), p=0.004 | Significantly fewer participants in the AVERT group had favourable outcome and there was no difference in mortality |
| Dello et al., 2021 (88) | Single-centre pre- and post-intervention study  Control n=238, intervention n=257 | Nurse-led management of temperature, glycaemia and swallowing (risk factors) | Mortality and functional status (mRS ≥2) at 90 days | The rates of 90-day death and dependency (mRS ≥2) were lower in the post-intervention period (127/248 [51%]) compared to the pre-intervention period (140/232 [60%]), adjusted OR 0.63, 95% CI: 0.41-0.97. | The intervention in the study was associated with significantly lower death and dependency compared with control |
| He et al., 2023 (39) | Randomized, historically controlled clinical trial  Control n=124, intervention n=161 | Rapid recovery implementation process to optimise patient recovery post-surgery | Functional status (mRS) | mRS score at discharge: 2.52 (1.89) Control, 3.61 (5.74) intervention, p<0.001  mRS score 3 month after discharge: 2.38 (1.77) (control), 2.01 (1.95) (intervention), p = 0.097 | Statistically significant differences in mRS were seen at discharge but not at 3 months. |
| Luengo-Fernandez et al., 2022 (EXPRESS) (16) | Prospective population-based before (phase 1: April 2002–September 2004; n=310) versus after (phase 2: October 2004–March 2007; n=281) study  EXPRESS: Phase 1 n=310, Phase 2 n=281; Control: Phase 1 n=167, Phase 2 n=224 | EXPRESS Phase 1: Clinic referral (1-2 days), brain imaging and ECG, ultrasound/echo, primary care treatment protocol comprising antiplatelet, statin, BP lowering. Phase 2: As Phase 1 but treatment initiated immediately instead of by primary care, high-dose aspirin or clopidogrel, CT scan during clinic. Follow-up 1, 6, 12, 60, and 120 months after the index event in both Phases. Control: standard care (not referred to study clinic) | Mortality, functional status (mRS), recurrent stroke, quality of life (EQ-5D) | Disability (mRS>2), n (%):  1 month: Control Phase 1 47/167 (33), Phase 1 55/310 (18), control Phase 2 58/224 (32), Phase 2 34/281 (13)  6 months: Control Phase 1 47/167 (34), Phase 1 71/310 (24), control Phase 2 50/224 (29), Phase 2 34/281 (13)*  1 year: Control Phase 1 43/167 (33), Phase 1 60/310 (22), Control Phase 2 47/224 (29), Phase 2 36/281 (14)**  5 years: Control Phase 1 27/167 (34), Phase 1 51/310 (25), Control Phase 2 37/224 (32), Phase 2 50/281 (29)  10 years: Control Phase 1 9/167 (21), Phase 1 44/310 (36). Control Phase 2 22/224 (35), Phase 2 30/281 (27)  Statistically significant at: *p<0.001 & ** p<0.050 when compared to phase 1 | Urgent assessment and treatment of patients with transient ischemic attack or minor stroke resulted in a long-term reduction in recurrent strokes and improved outcomes, with little atrophy of the early benefit over time, representing good value for money even with a 10-year time horizon.  A reduction in stroke risk in phase 2 was still evident at 10 years (55/23.3% versus 82/31.6%; hazard ratio=0.68 [95% CI, 0.48–0.95]; P=0.024), as was the impact on risk of disabling or fatal stroke (17/7.7% versus 32/13.1%; hazard ratio=0.54 [0.30–0.97]; P=0.036). |
| Rasmussen et al., 2016 (85) | Interventional, randomised, safety/efficacy open-label trial  Control n=30, intervention n=31 | Multidisciplinary home-based pathway including exercise and goal setting | Primary: mRS at 90 days | mRS:  Modified Rankin Scale: Intervention: 2 (2–3); Control: 3 (2–4)  Modified Rankin Scale improvement: Intervention: 1 (1–2); Control: 1 (0–1) | Early home-based rehabilitation reduced disability and increased quality of life. Compared to standard care, home-based stroke rehabilitation was more cost-effective. |
| Sharma et al., 2019 (COMPASS) (28) | COMPASS was a randomized, double-blind, double-dummy trial  Prior stroke: rivaroxaban + aspirin n=351, rivaroxaban alone n=346, aspirin alone n=335. Total n=1032 | Participants had stable coronary artery or peripheral artery disease and were randomly assigned to receive aspirin 100 mg once daily (n=9126), rivaroxaban 5 mg twice daily (n=9117), or rivaroxaban 2.5 mg twice daily plus aspirin (n=9152). | Functional status (mRS) at 23 months | mRS at 7 days or discharge  R+A (n=83) 2.4±2.0, R (n=115) 2.6±2.1, A (n=141) 2.2±1.  R+A vs A mean difference (95% CI) 0.2 (–0.4 to 0.7), p=0.66  R vs A mean difference (95% CI) 0.3 (–0.1 to 0.8), p=0.29 | In summary, low-dose rivaroxaban plus aspirin is an important new option for efficacious antithrombotic therapy for primary and especially secondary prevention of stroke in patients with atherosclerosis. The absolute risk reduction for secondary prevention is substantial and makes a compelling case favoring the use of 2.5 mg rivaroxaban twice daily plus aspirin in these patients. |
| Willeit et al., 2020 (STROKE-CARD) (24) | Pragmatic open-label two-centre randomised controlled trial with blinded outcome assessment of STROKE-CARD disease management programme and standard care  Control n=711, intervention n=1438 | STROKE-CARD care is a disease management programme by a multidisciplinary stroke team that comprises a standardised 3-month visit and access to a web-based patient portal targeting risk factor management, post-stroke complications, comorbidities and cardiovascular warning signs, rehabilitation demands, and patient education, counselling, and self-empowerment. | Functional status (mRS) at 12 months | mRS ≤2: Intervention 1150/1413 (81.4%), control 536/704 (76.1%), HR 1.07 (1.02, 1.12), P=0.006 | The pragmatic and easily implementable STROKE-CARD care programme reduced cardiovascular risk and improved health-related quality of life and functional outcome in patients with acute ischaemic stroke or TIA |

## Recurrent risk of cardiovascular disease


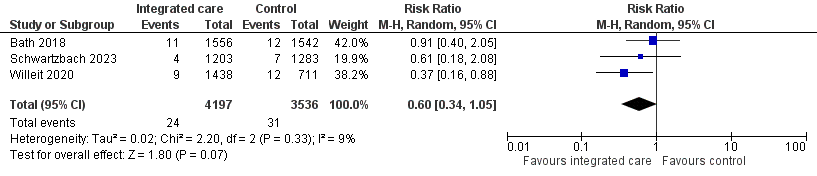


**Figure S11: Comparison of integrated care with control for myocardial infarction**

A meta analysis of 3 studies (n=7,317) showed no significant difference in the rates of vascular death between groups (RR, 0.76, 95% CI: 0.35, 1.63, p=0.48, I^2^ = 75%, Figure S12).


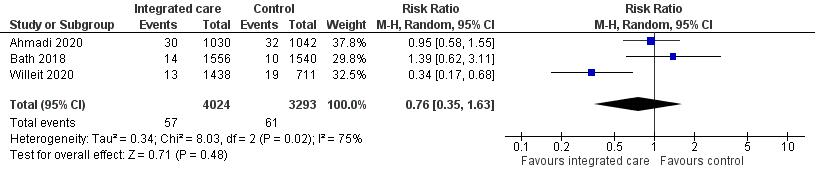


**Figure S12: Comparison of integrated care with control for vascular death**

Table S10: Analysis of recurrent risk of cardiovascular disease (myocardial infarction)

| **Study details** | **Design/Patients** | **Intervention** | **Outcome** | **Results** | **Comments** |
| --- | --- | --- | --- | --- | --- |
| Bath et al., 2018 (TARDIS) (2) | International (UK, Denmark, Georgia, NZ) prospective randomised open-label blinded end-point superiority clinical trial  Guideline (control) n=1540, Intervention n=1556 | Intensive treatment group given combined aspirin (load 300mg, maintenance 50-150mg daily, typically 75mg), clopidogrel (load 300mg, maintenance 75mg daily), and dipyridamole (200mg twice daily modified release, given orally; or 100mg three or four times daily). | MI at 90 days | MI at Day 90, n (%):  Intervention 11/1556 (0.7%)  Control 12/1540 (0.8%) | No difference in MI between groups |
| Schwartzbach et al., 2023 (SANO) (22) | Prospective, open-label, cluster-randomised controlled trial  Control n=1283, intervention n=1203 | 1-year patient-centred integrated care intervention including regular follow-up, lifestyle, goal setting and motivational interviewing (SANO) | MI at 12 months | MI at 12 months, n (%)  Intervention: 4/1203 (0.3)  Control: 7/1283 (0.5)  OR, 0.61 (95% CI: 0.18-2.08) | No difference in MI between groups |
| Willeit et al., 2020 (STROKE-CARD) (24) | Pragmatic open-label two-centre randomised controlled trial with blinded outcome assessment of STROKE-CARD disease management programme and standard care  Control n=711, intervention n=1438 | STROKE-CARD care is a disease management programme by a multidisciplinary stroke team that comprises a standardised 3-month visit and access to a web-based patient portal targeting risk factor management, post-stroke complications, comorbidities and cardiovascular warning signs, rehabilitation demands, and patient education, counselling, and self-empowerment. | MI at 12 months | MI at 12 months, n (%): Intervention: 9/1438 (0.6)  Control: 12/711 (1.7)  HR, 0.32, 95% CI: 0.13-0.80, p=0.014 | Risk of MI was significantly lower in the STROKE-CARD group |

Table S11: Analysis of recurrent risk of cardiovascular disease (vascular death)

| **Study details** | **Design/Patients** | **Intervention** | **Outcome** | **Results** | **Comments** |
| --- | --- | --- | --- | --- | --- |
| Ahmadi et al., 2020 (INSPiRE-YMS) (1) | Prospective, randomised, open­label, blinded endpoint, event-­driven trial  Control n=1042, intervention n=1030 | Screening for complications, QoL, depression | Vascular death (mean follow-up 3.6 years) | Vascular death, n (%): Intervention 30/1030 (2.9%)  Control 32/1042 (3.1%)  HR 0.94 (0.57-1.54) | No difference in vascular death rates between groups |
| Bath et al., 2018 (TARDIS) (2) | International (UK, Denmark, Georgia, NZ) prospective randomised open-label blinded end-point superiority clinical trial  Guideline (control) n=1540, Intervention n=1556 | Intensive treatment group given combined aspirin (load 300mg, maintenance 50-150mg daily, typically 75mg), clopidogrel (load 300mg, maintenance 75mg daily), and dipyridamole (200mg twice daily modified release, given orally; or 100mg three or four times daily). | Vascular death at Day 90 | Vascular death at Day 90, n (%):  Intervention 14/1556 (0.9%)  Control 10/1540 (0.7% | No difference in vascular death rates between groups |
| Willeit et al., 2020 (STROKE-CARD) (24) | Pragmatic open-label two-centre randomised controlled trial with blinded outcome assessment of STROKE-CARD disease management programme and standard care  Control n=711, intervention n=1438 | STROKE-CARD care is a disease management programme by a multidisciplinary stroke team that comprises a standardised 3-month visit and access to a web-based patient portal targeting risk factor management, post-stroke complications, comorbidities and cardiovascular warning signs, rehabilitation demands, and patient education, counselling, and self-empowerment. | Vascular death at 12 months | Vascular death at 12 months, n (%):  Intervention: 13/1438 (0.9)  Control: 19/711 (2.7)  HR, 0.32, 95% CI: 0.16-0.66, p=0.002 | Risk of vascular death was significantly lower in the STROKE-CARD group |

# References

1. Ahmadi M, Laumeier I, Ihl T, Steinicke M, Ferse C, Endres M, et al. A support programme for secondary prevention in patients with transient ischaemic attack and minor stroke (INSPiRE-TMS): an open-label, randomised controlled trial. The lancet Neurology. 2020;19(1):49‐60.

2. Bath PM, Woodhouse LJ, Appleton JP, Beridze M, Christensen H, Dineen RA, et al. Triple versus guideline antiplatelet therapy to prevent recurrence after acute ischaemic stroke or transient ischaemic attack: the TARDIS RCT. Health Technol Assess. 2018;22(48):1-76.

3. Bergh E, Askim T, Rønning OM, Šaltyteė Benth J, Fjærtoft H, Thommessen B. Does implementation of a standardized pathway of stroke care affect functional outcome after stroke? International journal of stroke : official journal of the International Stroke Society. 2023;18(5):578-85.

4. Bernhardt J, Langhorne P, Lindley RI, Thrift AG, Ellery F, Collier J, et al. Efficacy and safety of very early mobilisation within 24 h of stroke onset (AVERT): a randomised controlled trial. Lancet. 2015;386(9988):46-55.

5. Cuccurullo SJ, Fleming TK, Zinonos S, Cosgrove NM, Cabrera J, Kostis JB, et al. Stroke Recovery Program with Modified Cardiac Rehabilitation Improves Mortality, Functional & Cardiovascular Performance. Journal of stroke and cerebrovascular diseases : the official journal of National Stroke Association. 2022;31(5):106322.

6. Buckley BJR, Harrison SL, Fazio-Eynullayeva E, Underhill P, Lane DA, Thijssen DHJ, et al. Exercise-Based Cardiac Rehabilitation Associates with Lower Major Adverse Cardiovascular Events in People with Stroke. Cerebrovasc Dis. 2022;51(4):488-92.

7. Bushnell CD, Kucharska-Newton AM, Jones SB, Psioda MA, Johnson AM, Daras LC, et al. Hospital Readmissions and Mortality Among Fee-for-Service Medicare Patients With Minor Stroke or Transient Ischemic Attack: findings From the COMPASS Cluster-Randomized Pragmatic Trial. Journal of the American Heart Association. 2021;10(23):e023394.

8. Chang K-C, Hung J-W, Lee H-C, Yen C-L, Wu C-Y, Yang C-L, et al. Rehabilitation Reduced Readmission and Mortality Risks in Patients With Stroke or Transient Ischemic Attack: A Population-based Study. Medical care. 2018;56(4):290-8.

9. Chen CM, Yang YH, Chang CH, Chen PC. Effects of Transferring to the Rehabilitation Ward on Long-Term Mortality Rate of First-Time Stroke Survivors: A Population-Based Study. Archives of physical medicine and rehabilitation. 2017;98(12):2399-407.

10. Cuccurullo SJ, Fleming TK, Kostis WJ, Greiss C, Gizzi MS, Eckert A, et al. Impact of a Stroke Recovery Program Integrating Modified Cardiac Rehabilitation on All-Cause Mortality, Cardiovascular Performance and Functional Performance. American Journal of Physical Medicine & Rehabilitation. 2019;98(11):953-63.

11. de Belvis AG, Lohmeyer FM, Barbara A, Giubbini G, Angioletti C, Frisullo G, et al. Ischemic stroke: clinical pathway impact. INTERNATIONAL JOURNAL OF HEALTH CARE QUALITY ASSURANCE. 2019;32(3):588-98.

12. Deutschbein J, Grittner U, Schneider A, Schenk L. Community care coordination for stroke survivors: results of a complex intervention study. BMC health services research. 2020;20(1):1143.

13. Duncan PW, Bushnell CD, Jones SB, Psioda MA, Gesell SB, D'Agostino RB, et al. Randomized Pragmatic Trial of Stroke Transitional Care: The COMPASS Study. Circulation Cardiovascular quality and outcomes. 2020;13(6):e006285.

14. Fu V, Weatherall M, McPherson K, Taylor W, McRae A, Thomson T, et al. Taking Charge after Stroke: A randomized controlled trial of a person-centered, self-directed rehabilitation intervention. International journal of stroke : official journal of the International Stroke Society. 2020;15(9):954-64.

15. Liu N, Cadilhac DA, Andrew NE, Zeng L, Li Z, Li J, et al. Randomized controlled trial of early rehabilitation after intracerebral hemorrhage stroke: difference in outcomes within 6 months of stroke. Stroke (00392499). 2014;45(12):3502-7.

16. Luengo-Fernandez R, Li L, Silver L, Gutnikov S, Beddows NC, Rothwell PM. Long-Term Impact of Urgent Secondary Prevention After Transient Ischemic Attack and Minor Stroke: Ten-Year Follow-Up of the EXPRESS Study. Stroke. 2022;53(2):488-96.

17. Man S, Zhao X, Uchino K, Hussain MS, Smith EE, Bhatt DL, et al. Comparison of Acute Ischemic Stroke Care and Outcomes Between Comprehensive Stroke Centers and Primary Stroke Centers in the United States. Circulation Cardiovascular quality and outcomes. 2018;11(6):e004512.

18. Mofidi R, Thomas M, Wong PF, Bergin A, Young G. Do Integrated Systems of Stroke Care Improve Symptom to Surgery Times in Patients with Symptomatic Carotid Stenosis? A Single Centre Decision Tree Analysis. Eur J Vasc Endovasc Surg. 2018;56(6):784-92.

19. Pedapati R, Bhatia R, Shakywar M, Gupta A, Vishnubhatla S, Srivastava MVP, et al. Educating Caregivers to Reduce Complications and Improve Outcomes of Stroke Patients (ECCOS)-A Cluster-Randomized Trial. JOURNAL OF STROKE & CEREBROVASCULAR DISEASES. 2021;30(9).

20. Peng LN, Lu WH, Liang CK, Chou MY, Chung CP, Tsai SL, et al. Functional Outcomes, Subsequent Healthcare Utilization, and Mortality of Stroke Postacute Care Patients in Taiwan: A Nationwide Propensity Score-matched Study. Journal of the American Medical Directors Association. 2017;18(11):990.e7-.e12.

21. Saal S, Becker C, Lorenz S, Schubert M, Kuss O, Stang A, et al. Effect of a stroke support service in Germany: a randomized trial. TOPICS IN STROKE REHABILITATION. 2015;22(6):429-36.

22. Schwarzbach CJ, Eichner FA, Rücker V, Hofmann AL, Keller M, Audebert HJ, et al. The structured ambulatory post-stroke care program for outpatient aftercare in patients with ischaemic stroke in Germany (SANO): an open-label, cluster-randomised controlled trial. Lancet Neurol. 2023;22(9):787-99.

23. Swanson JO, Moger TA. Comparisons of readmissions and mortality based on post-discharge ambulatory follow-up services received by stroke patients discharged home: a register-based study. BMC health services research. 2019;19(1):4.

24. Willeit P, Toell T, Boehme C, Krebs S, Mayer L, Lang C, et al. STROKE-CARD care to prevent cardiovascular events and improve quality of life after acute ischaemic stroke or TIA: A randomised clinical trial. EClinicalMedicine. 2020;25:100476.

25. Cheng EM, Cunningham WE, Towfighi A, Sanossian N, Bryg RJ, Anderson TL, et al. Efficacy of a Chronic Care-Based Intervention on Secondary Stroke Prevention Among Vulnerable Stroke Survivors: a Randomized Controlled Trial. Circulation Cardiovascular quality and outcomes. 2018;11(1):e003228.

26. Nguyen-Huynh MN, Klingman JG, Avins AL, Rao VA, Eaton A, Bhopale S, et al. Novel Telestroke Program Improves Thrombolysis for Acute Stroke Across 21 Hospitals of an Integrated Healthcare System. Stroke. 2018;49(1):133-9.

27. Reeves MJ, Fritz MC, Woodward AT, Hughes AK, Coursaris CK, Swierenga SJ, et al. Michigan Stroke Transitions Trial. Circulation Cardiovascular quality and outcomes. 2019;12(7):e005493.

28. Sharma M, Hart RG, Connolly SJ, Bosch J, Shestakovska O, Ng KKH, et al. Stroke Outcomes in the COMPASS Trial. Circulation. 2019;139(9):1134-45.

29. Yan LL, Gong E, Gu W, Turner EL, Gallis JA, Zhou Y, et al. Effectiveness of a primary care-based integrated mobile health intervention for stroke management in rural China (SINEMA): A cluster-randomized controlled trial. PLoS Med. 2021;18(4):e1003582.

30. Abdul Aziz Aznida Furzah, Azlin MNN, Amrizal MN, Saperi S, Mohamed AS. The integrated care pathway for managing post stroke patients (iCaPPS © ) in public primary care Healthcentres in Malaysia: impact on quality adjusted life years (QALYs) and cost effectiveness analysis. BMC geriatrics. 2020;20(1):70.

31. Bragstad LK, Hjelle EG, Zucknick M, Sveen U, Thommessen B, Bronken BA, et al. The effects of a dialogue-based intervention to promote psychosocial well-being after stroke: a randomized controlled trial. Clinical rehabilitation. 2020;34(8):1056‐71.

32. Brouns B, Van Bodegom-Vos L, De Kloet AJ. Tamminga SJ, Volker G, Berger MAM, et al. Effect of a Comprehensive erehabilitation Intervention Alongside Conventional Stroke Rehabilitation on Disability and Health-related Quality of Life: a Pre-post comparison. Journal of Rehabilitation Medicine (Stiftelsen Rehabiliteringsinformation). 2021;53(3):1-12.

33. Brouwer B, Bryant D, Garland SJ. Effectiveness of Client-Centered "Tune-Ups" on Community Reintegration, Mobility, and Quality of Life After Stroke: a Randomized Controlled Trial. Archives of physical medicine and rehabilitation. 2018;99(7):1325‐32.

34. Chiu C-C, Lin H-F, Lin C-H, Chang H-T, Hsien H-H, Hung K-W, et al. Multidisciplinary Care after Acute Care for Stroke: A Prospective Comparison between a Multidisciplinary Post-Acute Care Group and a Standard Group Matched by Propensity Score. International journal of environmental research and public health. 2021;18(14).

35. Chu K, Bu X, Sun Z, Wang Y, Feng W, Xiao, L, et al. Feasibility of a Nurse-Trained, Family Member-Delivered Rehabilitation Model for Disabled Stroke Patients in Rural Chongqing, China. Journal of stroke and cerebrovascular diseases : the official journal of National Stroke Association. 2020;29(12):105382.

36. Cumming TB, Churilov L, Collier J, Donnan G, Ellery F, Dewey H, et al. Early mobilization and quality of life after stroke: Findings from AVERT. Neurology. 2019;93(7):e717-e28.

37. Døhl Ø, Halsteinli V, Askim T, Gunnes M, Ihle-Hansen H, Indredavik B, et al. Factors contributing to post-stroke health care utilization and costs, secondary results from the life after stroke (LAST) study. BMC health services research. 2020;20(1):288.

38. Geng G, He W, Ding L, Klug D, Xiao Y. Impact of transitional care for discharged elderly stroke patients in China: an application of the Integrated Behavioral Model. TOPICS IN STROKE REHABILITATION. 2019;26(8):621-9.

39. He Y, Wang R, Dong S, Long S, Zhang P, Feng L. Nurse-led rapid rehabilitation following mechanical thrombectomy in patients with acute ischemic stroke: A historical control study. Medicine. 2023;102(28):e34232.

40. Hjelle EG, Bragstad LK, Kirkevold M, Zucknick M, Bronken BA, Martinsen R, et al. Effect of a dialogue-based intervention on psychosocial well-being 6 months after stroke in Norway: A randomized controlled trial. Journal of rehabilitation medicine. 2019;51(8):557-65.

41. Jones F, Gage H, Drummond A, Bhalla A, Grant R, Lennon S, et al. Feasibility study of an integrated stroke self-management programme: a cluster-randomised controlled trial. BMJ OPEN. 2016;6(1).

42. Kalav S, Bektas H, Ünal A. Effects of Chronic Care Model‐based interventions on self‐management, quality of life and patient satisfaction in patients with ischemic stroke: A single‐blinded randomized controlled trial. Japan Journal of Nursing Science. 2022;19(1):1-18.

43. Kam Yuet Wong F, Wang SL, Ng SSM, Lee PH, Wong AKC, Li H, et al. Effects of a transitional home-based care program for stroke survivors in Harbin, China: a randomized controlled trial. Age Ageing. 2022;51(2).

44. Khramov W, Kogaeva KP, Arkhipova, LU, Alekseeva, VO, Lukyanova, MI. EFFECTIVENESS OF POST-STROKE SOCIAL REHABILITATION IN PATIENTS WITH MODERATE IMPAIRMENTS. BULLETIN OF RUSSIAN STATE MEDICAL UNIVERSITY. 2021(2):62-8.

45. Langhorne P, Wu O, Rodgers H, Ashburn A, Bernhardt J. A Very Early Rehabilitation Trial after stroke (AVERT): a Phase III, multicentre, randomised controlled trial. Health Technol Assess. 2017;21(54):1-120.

46. Lewthwaite R, Winstein CJ, Lane CJL, Blanton S, Wagenheim BR, Nelsen MA, Dromerick AW, et al. Accelerating Stroke Recovery: Body Structures and Functions, Activities, Participation, and Quality of Life Outcomes From a Large Rehabilitation Trial. Neurorehabilitation & Neural Repair. 2018;32(2):150-65.

47. Winstein CJ, Wolf SL, Dromerick AW, Lane CJ, Nelsen MA, Lewthwaite R, et al. Interdisciplinary Comprehensive Arm Rehabilitation Evaluation (ICARE): a randomized controlled trial protocol. BMC Neurol. 2013;13:5.

48. Liu LM. EFFECTS OF TRANSITIONAL CARE ON FUNCTIONAL EXERCISE: COMPLIANCE AND HEALTH STATUS OF STROKE PATIENTS. ACTA MEDICA MEDITERRANEA. 2018;34(4):959-65.

49. Markle-Reid M, Valaitis R, Bartholomew A, Fisher K, Fleck R, Ploeg J, et al. An integrated hospital-to-home transitional care intervention for older adults with stroke and multimorbidity: A feasibility study. Journal of Comorbidity. 2020;10:1-21.

50. Minshall C, Castle DJ, Thompson DR, Pascoe M, Cameron J, McCabe M, et al. A psychosocial intervention for stroke survivors and carers: 12-month outcomes of a randomized controlled trial. TOPICS IN STROKE REHABILITATION. 2020;27(8):563-76.

51. Mohammadi E, Hassandoost F, Mozhdehipanah H. Evaluation of the "partnership care model" on quality of life and activity of daily living in stroke patients: A randomized clinical trial. Japan journal of nursing science : JJNS. 2022;19(1):e12448.

52. Rodgers H, Howel D, Bhattarai N, Cant R. Drummond A, Ford GA, et al. Evaluation of an Extended Stroke Rehabilitation Service (EXTRAS): A Randomized Controlled Trial and Economic Analysis. STROKE. 2019;50(12):3561-8.

53. Shaw L, Bhattarai N, Cant R, Drummond A, Ford GA, Forster A, et al. An extended stroke rehabilitation service for people who have had a stroke: the EXTRAS RCT. HEALTH TECHNOLOGY ASSESSMENT. 2020;24(24):1-+.

54. Tung YJ, Lin WC, Lee LF, Lin HM, Ho CH, Chou W. Comparison of Cost-Effectiveness between Inpatient and Home-Based Post-Acute Care Models for Stroke Rehabilitation in Taiwan. INTERNATIONAL JOURNAL OF ENVIRONMENTAL RESEARCH AND PUBLIC HEALTH. 2021;18(8).

55. Vluggen TPMM, van Haagstreg, JCM. Tan FE, Verbunt JA, van Heugten CM, Schols, JMG. A. Effectiveness of an integrated multidisciplinary geriatric rehabilitation programme for older persons with stroke: a multicentre randomised controlled trial. BMC geriatrics. 2021;21(1):134.

56. Long Weijan, Zhang J. Patient Caregiver Comprehensive Rehabilitation Nursing Training Joint Continued nursing care for ischemic stroke patients at home Impact on quality of life and activities of daily living. Chinese Nursing Research. 2017;31(20):2456-61.

57. Wong FKY, Yeung SM. Effects of a 4-week transitional care programme for discharged stroke survivors in Hong Kong: a randomised controlled trial. Health & Social Care in the Community. 2015;23(6):619-31.

58. Wu Z, Xu J, Yue C, Li Y, Liang Y. Collaborative Care Model Based Telerehabilitation Exercise Training Program for Acute Stroke Patients in China: A Randomized Controlled Trial. Journal of stroke and cerebrovascular diseases : the official journal of National Stroke Association. 2020;29(12):105328.

59. Xi W, Duan Y, Wang A. Influence of continuing nursing outside hospital on rehabilitation effect and quality of life in patients with stroke. Chinese Nursing Research. 2017;31(29):3760-2.

60. Demir Avci Y, Gözüm S. Effects of Transitional Care Model-Based Interventions for Stroke Patients and Caregivers on Caregivers' Competence and Patient Outcomes: Randomized Controlled Trial. Computers, informatics, nursing : CIN. 2023;41(10):805-14.

61. Chen L, Sit JW-H, Shen X. Quasi-experimental evaluation of a home care model for patients with stroke in China. Disability & Rehabilitation. 2016;38(23):2271-6.

62. Cuccurullo SJ, Fleming TK, Kostis JB, Greiss C, Eckert A, Ray AR, et al. Impact of Modified Cardiac Rehabilitation Within a Stroke Recovery Program on All-Cause Hospital Readmissions. American journal of physical medicine & rehabilitation. 2022;101(1):40-7.

63. Wang J, Wang J, Qiu S, Zhou C, Zhang H, Li Q, et al. Pharmaceutical care program for ischemic stroke patients: a randomized controlled trial. Int J Clin Pharm. 2021;43(5):1412-9.

64. Crocker TF, Brown L, Lam N, Wray F, Knapp P, Forster A. Information provision for stroke survivors and their carers. Cochrane Database Syst Rev. 2021;11:CD001919.

65. Allida S, Cox KL, Hsieh C-F, Lang H, House A, Hackett ML. Pharmacological, psychological, and non-invasive brain stimulation interventions for treating depression after stroke. Cochrane Database of Systematic Reviews. 2020.

66. Brandal A, Eriksson E, Glader, EL, Wester P. Effect of early supported discharge after stroke on patient reported outcome based on the Swedish Riksstroke registry. BMC NEUROLOGY. 2019;19.

67. Cheng C, Liu X, Fan W, Bai X, Liu Z. Comprehensive Rehabilitation Training Decreases Cognitive Impairment, Anxiety, and Depression in Poststroke Patients: A Randomized, Controlled Study. Journal of stroke and cerebrovascular diseases : the official journal of National Stroke Association. 2018;27(10):2613-22.

68. Ellis-Hill C, Thomas S, Gracey F, Lamont-Robinson C, Cant R, et al. HeART of Stroke: randomised controlled, parallel-arm, feasibility study of a community-based arts and health intervention plus usual care compared with usual care to increase psychological well-being in people following a stroke. BMJ open. 2019;9(3):e021098.

69. Feng W, Yu H, Wang J, Xia J. Application effect of the hospital-community integrated service model in home rehabilitation of stroke in disabled elderly: a randomised trial. Annals of palliative medicine. 2021;10(4):4670-7.

70. Gao J, Lin M, Zhao J, Bi S, Ni Z, Shang X. Different interventions for post-ischaemic stroke depression in different time periods: a single-blind randomized controlled trial with stratification by time after stroke. Clinical rehabilitation. 2017;31(1):71-81.

71. Graven C, Brock K, Hill KD, Cotton S, Joubert L. First Year After Stroke: An Integrated Approach Focusing on Participation Goals Aiming to Reduce Depressive Symptoms. Stroke. 2016;47(11):2820-7.

72. Gun Young Yang, Min HS. The Effects of an Integrated Management Program on Physical Function, Cognitive Function, and Depression in Patients with Subacute stroke. Journal of Korean Critical Care Nursing. 2021;14(1):50-62.

73. Jung S-H, Park E, Kim J-H, Park, B-A, Yu J-W, et al. Effects of Self RehAbilitation Video Exercises (SAVE) on Functional Restorations in Patients with Subacute Stroke. HEALTHCARE. 2021;9(5).

74. Lee A-Y, Park S-A, Park H-G, Son K-C. Determining the Effects of a Horticultural Therapy Program for Improving the Upper Limb Function and Balance Ability of Stroke Patients. HORTSCIENCE. 2018;53(1):110-9.

75. Lin RC, Chiang SL, Heitkemper MM, Weng, SM, Lin CF, Yang FC, et al. Effectiveness of Early Rehabilitation Combined With Virtual Reality Training on Muscle Strength, Mood State, and Functional Status in Patients With Acute Stroke: A Randomized Controlled Trial. Worldviews on Evidence-Based Nursing. 2020;17(2):158-67.

76. Lo SHS, Chau JPC, Lau AYL, Choi KC, Shum EWC, Lee VWY, et al. Virtual Multidisciplinary Stroke Care Clinic for Community-Dwelling Stroke Survivors: A Randomized Controlled Trial. Stroke. 2023;54(10):2482-90.

77. MacKay-Lyons M, Gubitz G, Phillips S, Giacomantonio N, Firth W, Thompson K, et al. Program of Rehabilitative Exercise and Education to Avert Vascular Events After Non-Disabling Stroke or Transient Ischemic Attack (PREVENT Trial): a Randomized Controlled Trial. Neurorehabilitation and neural repair. 2022;36(2):119‐30.

78. Rafsten L, Danielsson A, Nordin A, Björkdahl A, Lundgren-Nilsson A, Larsson M, et al. Gothenburg Very Early Supported Discharge study (GOTVED): a randomised controlled trial investigating anxiety and overall disability in the first year after stroke. BMC neurology. 2019;19(1):277.

79. Teuschl Y, Matz K, Firlinger B, Dachenhausen A, Tuomilehto J, Brainin M, et al. Preventive effects of multiple domain interventions on lifestyle and risk factor changes in stroke survivors: Evidence from a two-year randomized trial. Int J Stroke. 2017;12(9):976-84.

80. Yu HL, Cao DX, Liu J. Effect of a novel designed intensive patient care program on cognitive impairment, anxiety, depression as well as relapse free survival in acute ischemic stroke patients: a randomized controlled study. NEUROLOGICAL RESEARCH. 2019;41(9):857-66.

81. Zhang L, Zhang T, Sun Y. A newly designed intensive caregiver education program reduces cognitive impairment, anxiety, and depression in patients with acute ischemic stroke. Braz J Med Biol Res. 2019;52(9):e8533.

82. Greger J, Wojcik R, Westphal E, Aladeen T, Landolf K, Boyce S, et al. Pharmacist intervention and anti-platelet medication monitoring in patients following stroke and transient ischemic attack. JOURNAL OF THE AMERICAN COLLEGE OF CLINICAL PHARMACY. 2021;4(3):311-7.

83. Joubert J, Davis SM, Donnan GA, Levi C, Gonzales G, Joubert L, et al. ICARUSS: An effective model for risk factor management in stroke survivors. INTERNATIONAL JOURNAL OF STROKE. 2020;15(4):438-53.

84. Olaiya MT, Kim J, Nelson MR, Srikanth VK, Bladin CF, Gerraty RP, et al. Effectiveness of a shared team approach between nurses and doctors for improved risk factor management in survivors of stroke: a cluster randomized controlled trial. Eur J Neurol. 2017;24(7):920-8.

85. Rasmussen RS, Østergaard A, Kjær P, Skerris A, Skou C, Christoffersen J, et al. Stroke rehabilitation at home before and after discharge reduced disability and improved quality of life: a randomised controlled trial. Clinical Rehabilitation. 2016;30(3):225-36.

86. Towfighi A, Cheng EM, Ayala-Rivera M, Barry F, McCreath H, Ganz D, et al. Secondary Stroke Prevention, Unitin. Effect of a Coordinated Community and Chronic Care Model Team Intervention vs Usual Care on Systolic Blood Pressure in Patients With Stroke or Transient Ischemic Attack The SUCCEED Randomized Clinical Trial. JAMA NETWORK OPEN. 2021;4(2).

87. Koch S, Tiozzo E, Simonetto M, Loewenstein D, Wright CB, Dong C, et al. Randomized Trial of Combined Aerobic, Resistance, and Cognitive Training to Improve Recovery From Stroke: Feasibility and Safety. Journal of the American Heart Association. 2020;9(10):e015377.

88. Dello S, Lemmens R, Demeestere J, Michiels D, Wellens L, Weltens C, et al. A nurse-led multicomponent intervention supported by advanced electronic health records to improve the acute management of stroke patients: A pre- and post-intervention study. INTERNATIONAL JOURNAL OF NURSING STUDIES ADVANCES. 2021;3.
